# Causal Associations of DNA Methylation and Cardiovascular Disease: A Two-Sample Mendelian Randomization Study

**DOI:** 10.5334/gh.1324

**Published:** 2024-05-14

**Authors:** Hui Gao, Jiahai Li, Qiaoli Ma, Qinghui Zhang, Man Li, Xiaoliang Hu

**Affiliations:** 1 Department of Cardiovascular Medicine, The First People’s Hospital of Shangqiu, Shangqiu 476000, China; 2 Graduate School, Dalian Medical University, Dalian, 116044, China; 3 Department of Cardiovascular Medicine, The First People’s Hospital of Qinzhou, Qinzhou 535000, China; 4 Department of Cardiovascular Medicine, Central Hospital of Zibo, Zibo 255000, China; 5 Department of Hypertension, Henan Provincial People’s Hospital, Zhengzhou 450000, China

**Keywords:** Mendelian randomization, methylation, cardiovascular disease

## Abstract

**Background:**

There is growing evidence that concentrations of DNA methylation are associated with cardiovascular disease; however, it is unclear whether this association reflects a causal relationship.

**Methods:**

We utilized a two-sample Mendelian randomization (MR) approach to investigate whether DNA methylation can affect the risk of developing cardiovascular disease in human life. We primarily performed the inverse variance weighted (IVW) method to analyze the causal effect of DNA methylation on multiple cardiovascular diseases. Additionally, to ensure the robustness of our findings, we conducted several sensitivity analyses using alternative methodologies. These analysis methods included maximum likelihood, MR-Egger regression, weighted median method, and weighted model methods.

**Results:**

Inverse variance weighted estimates suggested that an SD increase in DNA methylation Hannum age acceleration exposure increased the risk of cardiac arrhythmias (OR = 1.03, 95% CI 1.00–1.05, *p* = 0.0290) and atrial fibrillation (OR = 1.03, 95% CI 1.00–1.05, *p* = 0.0022). We also found that an SD increase in DNA methylation PhenoAge acceleration exposure increased the risk of heart failure (OR = 1.01, 95% CI 1.00–1.03, *p* = 0.0362). Exposure to DNA methylation-estimated granulocyte proportions was found to increase the risk of hypertension (OR = 1.00, 95% CI 1.00–1.0001, p = 0.0291). Exposure to DNA methylation-estimated plasminogen activator inhibitor-1 levels was found to increase the risk of heart failure (OR = 1.00, 95% CI 1.00–1.00, *p* = 0.0215).

**Conclusion:**

This study reveals a causal relationship between DNA methylation and CVD. Exposed to high levels of DNA methylation Hannum age acceleration inhabitants with an increased risk of cardiac arrhythmias and atrial fibrillation. DNA methylation PhenoAge acceleration levels exposure levels were positively associated with the increased risk of developing heart failure. This has important implications for the prevention of cardiovascular diseases.

## 1 Introduction

Cardiovascular disease (CVD) is defined as a group of cardiac and vascular diseases, including coronary artery disease (CAD), atrial fibrillation (AF), heart failure (HF), hypertrophic cardiomyopathy, hypertension, non-ischemic cardiomyopathy and so on. In 2020, CVD was responsible for nearly 19 million deaths worldwide, with an increase of 18.7% since 2010 [1]. Cardiovascular disease (CVD) remains the leading cause of mortality globally with an increase in estimated years of life lost, though treatment system for cardiovascular disease is well developed [2]. The cause of CVD is not completely understood, despite substantial great progress in prevention of CVD in recent years [3]. Thus, determining the protective or causative factors in CVD remains critical. Increased evidence suggested that epigenetics plays an important role in the pathogenesis of CVD [4]. Epigenetic modifications can alter the expression of genes without changing their sequences. Deciphering the epigenomic signatures linked to CVD could contribute to better understanding of their mechanisms and to the definition of new therapeutic targets and preventive strategies.

Biochemically, DNA methylation refers to the addition of a methyl group to DNA nucleotides, usually at the cytosine of cytosine-phosphate-guanine (CpG) sites. DNA methylation regulates gene expression through interactions with transcription factors and transcriptional machinery, though the directional relationship between these two quantities is complex [5,6,7]. Global and gene-specific changes in DNA methylation were associated with the onset and severity of essential hypertension, pulmonary arterial hypertension, and atherosclerotic lesions [8]. A recent systematic review reported that differential DNA methylation at specific genes, including inflammation-related genes, is associated with coronary heart disease and atherosclerosis [9].

Most current studies have investigated the relationship between DNA methylation and CVD, but almost all these studies were derived from cross-sectional design. Some studies were hypothesis-based, focusing for example, on lipid-related genes or on the ‘epigenetic clock’ (a measure of biological age). Epigenome-wide studies have been widely applied in the field of myocardial infarction (MI), stroke, and various subcategories of coronary artery disease. Relevant cohort studies have shown that the characterization of gene function related to these DNA methylation sites can help identify the biological link between arsenic exposure and CVD [10]. Considering the CVD were common in middle-aged and elderly people, and DNA methylation was associated with aging, therefore, it is of great value to investigate the specific effect of DNA methylation on CVD. Genetic tools are used as instrumental variables (IVs) in the Mendelian randomization (MR) design to distinguish between correlation and causality in observed data [11]. Mendelian randomization is an alternative approach that can suggest causality of epigenetic associations. The rapid development of genome-wide association studies (GWAS) has led to the increasing application of Mendelian randomization (MR) analysis using the phenotypic-associated SNPs as instrumental variables (IVs) [12]. Besides, the two-sample MR has become the most widely used causal inference method for its advantage of using publicly available databases [13,14]. The MR method allows examining causal association regardless of research costs and ethics. Moreover, the alleles used as IVs were randomly allocated at conception, making the MR method a ‘natural randomised control trial’ [15]. Therefore, MR is sometimes an excellent research tool for causal inference investigation.

Therefore, we performed the two-sample MR analyses to investigate the causal effect of DNA methylation related parameters (DNA methylation-estimated granulocyte proportions, DNA methylation Grim Age acceleration, DNA methylation Hannum age acceleration, DNA methylation-estimated plasminogen activator inhibitor-1 levels, DNA methylation PhenoAge acceleration) on different CVD events (myocardial infarction, pulmonary heart disease, atrial fibrillation, heart failure, hypertrophic cardiomyopathy, cardiomyopathy, coronary heart disease, ischemic heart diseases, valvular heart disease).

## 2 Methods

In our research, we utilized the two-sample Mendelian randomization (MR) approach to investigate the causal relationship between DNA methylation and the likelihood risk of developing CVD. We obtained publicly available summary datasets from two genome-wide association studies (GWAS) as our primary data sources. Our study focused on several types of DNA methylation, including DNA methylation-estimated granulocyte proportions, DNA methylation GrimAge acceleration, DNA methylation Hannum age acceleration, DNA methylation-estimated plasminogen activator inhibitor-1 levels, and DNA methylation PhenoAge acceleration. The interesting outcomes were the occurrence of each CVD. In our study, the CVD included hypertension (cases/controls: 55917/162837; GWASID: finn-b-I9_HYPTENS), pulmonary heart disease (cases/controls: 4564/214228; GWASID: finn-b-I9_PULMHEART), cardiac arrhythmias (cases/controls: 32416/139739; GWASID: finn-b-CARDIAC_ARRHYTM), cardiomyopathy (cases/controls: 3,100/156,711; GWASID: finn-b-I9_CARDMYO), coronary heart disease (cases/controls: 60,801/123,504; GWASID: ieu-a-7), hypertrophic cardiomyopathy (cases/controls: 556/218,236; GWASID: finn-b-I9_HYPERTROCARDMYOP), ischemic heart diseases (cases/controls: 30,952/2187,840; GWASID: finn-b-I9_ISCHHEART), myocardial infarction (cases/controls: 43,676/128,199; GWASID: ieu-a-798), non-ischemic cardiomyopathy (cases/controls: 11,400/175,752; GWASID: finn-b-I9_NONISCHCARDMYOP), valvular heart disease (cases/controls: 38,209/180,583; GWASID: finn-b-I9_VHD_EXNONE), heart failure (cases/controls: 47309/930014; GWASID: ebi-a-GCST009541), atrial fibrillation (cases/controls: 60620/970216; GWASID: ebi-a-GCST006414). They included each dataset as shown in Table 1. To establish instrumental variables (IVs), we carefully selected specific single-nucleotide polymorphisms (SNPs) that exhibited robust associations with the various forms of DNA methylation. The MR framework operates based on three crucial assumptions:

- The genetic IVs were significantly associated with the exposure (DNA methylation).
- The genetic IVs were independent of potential confounding factors.
- The genetic IVs solely influence the outcome (cardiovascular disease) through the exposure (DNA methylation).

**Table 1:** The detailed information on genome-wide summary association studies (GWAs) used in the Mendelian randomization (MR) study.


GWAS ID	EXPOSURES OROUTCOME	CASE	CONTROL	SAMPLE SIZE	POPULATION

ebi-a-GCST90014287	DNA methylation-estimated granulocyte proportions	–	–	34470	European

ebi-a-GCST90014288	DNA methylation GrimAge acceleration	–	–		European

ebi-a-GCST90014289	DNA methylation Hannum age acceleration	–	–	34449	European

ebi-a-GCST90014291	DNA methylation-estimated plasminogen activator inhibitor-1 levels	–	–	34448	European

ebi-a-GCST90014292	DNA methylation PhenoAge acceleration	–	–	34463	European

finn-b-I9_HYPTENS	Hypertension	55917	162837	218754	European

finn-b-I9_PULMHEART	Pulmonary heart disease	4564	214228	218792	European

finn-b-CARDIAC_ARRHYTM	Cardiac arrhythmias	32416	139739	172155	European

finn-b-I9_CARDMYO	Cardiomyopathy	3,100	156,711	159811	European

ieu-a-7	Coronary heart disease	60,801	123,504	184305	European

finn-b-I9_HYPERTROCARDMYOP	Hypertrophic cardiomyopathy	556	218,236	218792	European

finn-b-I9_ISCHHEART	Ischemic heart diseases	30,952	187,840	218792	European

ieu-a-798	Myocardial infarction	43,676	128,199	171875	European

finn-b-I9_NONISCHCARDMYOP	Non-ischemic cardiomyopathy	11,400	175,752	187152	European

finn-b-I9_VHD_EXNONE	Valvular heart disease	38,209	180,583	218792	European

ebi-a-GCST009541	Heart failure	47309	930014	977323	European

ebi-a-GCST006414	Atrial fibrillation	60620	970216	1030836	European


**Figure 1:**
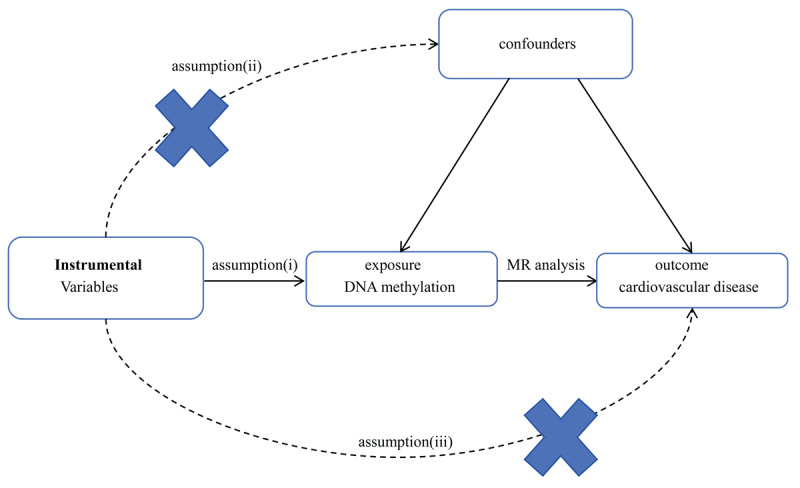
Study design flowchart of the Mendelian randomization study. The Mendelian randomization method is based on three hypotheses: 1. The instrumental variables is closely related to exposure. 2. Instrumental variables is independent of any confounding factor. 3. Instrumental variables affects the results only through exposure but not through other ways.

By adhering to these assumptions, our aim was to investigate the causal relationship between DNA methylation and the risk of CVD. An overview of the research design is presented in Figure 1. All the original studies obtained ethical approval and informed consent. This study was conducted based on the latest (STROBE-MR) guidelines [16].

### 2.1 Summary data resources

#### DNA methylation

For our analysis on DNA methylation exposure, we sourced the summary genetic data from McCartney DL the literature [17]. This dataset was publicly available through the MRC-IEU Open GWAS data. The specific GWAS-IDs used were ebi-a-GCST90014287 for DNA methylation-estimated granulocyte proportions (N = 34,470), ebi-a-GCST90014288 for DNA methylation GrimAge acceleration (N = 34,467), ebi-a-GCST90014289 for DNA methylation Hannum age acceleration (N = 34,449), ebi-a-GCST90014291 for DNA methylation-estimated plasminogen activator inhibitor-1 levels (N = 34,448), and ebi-a-GCST90014292 for DNA methylation PhenoAge acceleration (N = 34,463). The primary objective of this project was to examine the impacts of DNA methylation on various health outcomes.

#### Cardiovascular disease

Our analysis incorporated a total of 12 cardiovascular diseases, with varying case numbers, ranging from 556 (hypertrophic cardiomyopathy) to 60,801 (coronary heart disease). To obtain summary statistics for the associations between the instrumental variables (IVs) and cardiovascular disease, we extracted data from different consortiums. Specifically, we sourced information from the Coronary ARtery DIsease Genome-wide Replication and Meta-analysis plus The Coronary Artery Disease Genetics (CARDIoGRAMplusC4D) consortium for coronary artery disease and myocardial infarction, the Heart Failure Molecular Epidemiology for Therapeutic Targets (HERMES) consortium for heart failure, and the AFGen consortium for atrial fibrillation. Additionally, we obtained GWAS summary statistics for the remaining cardiovascular diseases from the FinnGen-R5 consortium. These diseases included hypertension, ischemic heart disease, non-ischemic cardiomyopathy, cardiomyopathy, hypertrophic cardiomyopathy, valvular heart disease, and pulmonary heart disease.

### 2.2 Selection of instrumental variables

To identify genetic predictors associated with DNA methylation characteristics, we implemented rigorous quality control procedures. Initially, we employed a stringent genome-wide significance threshold of P < 5 × 10^–8^ to identify highly associated single-nucleotide polymorphisms (SNPs) linked to DNA methylation. However, due to a limited number of eligible instrumental variables (IVs), we applied a relatively comprehensive threshold of P < 5 × 10^–6^ to capture a broader set of SNPs for more comprehensive results. To ensure adherence to the assumptions of Mendelian randomization (MR), we conducted a linkage disequilibrium (LD) analysis using data from the European-based 1,000 Genomes Project. SNPs that did not meet the criteria (R^2^ < 0.001, clumping distance = 10,000 kb) were excluded from further analysis. We also excluded palindromic SNPs due to uncertainties regarding their alignment in the same direction for both exposure and outcome in the cardiovascular disease genome-wide association studies. Additionally, SNPs with a minor allele frequency (MAF) below 0.01 were excluded from the raw data. In cases that SNPs associated with the exposure variable were missing in the outcome GWAS dataset, we selected proxy SNPs with high linkage disequilibrium (r^2^ > 0.80) to ensure comprehensive coverage. To assess the instrument strength, we calculated the F statistic using the formula F = R^2^(n–2)/(1–R^2^), where R^2^ represents the proportion of variance explained by the instrumental variables, and n represents the sample size. SNPs with F value < 10 were regarded as weak IVs.

### 2.3 Statistical analysis

To analyze the Mendelian randomization (MR) data, we primarily employed the inverse variance weighted (IVW) method. Additionally, to ensure the robustness of our findings, we conducted several sensitivity analyses using alternative methodologies. These analysis methods included maximum likelihood method, MR-Egger regression method, weighted median method, and weighted model method. In cases where the IVW method yielded statistically significant results (*p* < 0.05), we considered the outcome positive, even if other methods did not reach significance, as long as the direction of the beta values remained consistent. To assess the impact on cardiovascular disease, we estimated odds ratios (OR) with their corresponding 95% confidence intervals (CIs), applying a significance threshold of *p* < 0.05. Heterogeneity was evaluated using Cochran’s Q test for the IVW and MR-Egger estimates. We utilized the MR-Egger regression technique to explore potential pleiotropic bias. To evaluate the stability of our findings, we conducted a systematic ‘leave-one-out’ analysis, sequentially excluding each single-nucleotide polymorphism (SNP) to assess its influence on the overall results. All statistical analyses were performed using the ‘TwoSampleMR’ package (version 0.5.5) within the R software environment (version 4.0.3). These stringent analytical approaches were implemented to ensure the reliability and validity of the study’s outcomes.

## 3 Results

### 3.1 Selection of genetic instruments

In order to investigate the relationship between DNA methylation and the risk of CVD, we performed a Mendelian randomization (MR) analysis involving five DNA methylation traits to match CVD. Strong genetic instruments (p-values < 5 × 10^–6^) were obtained for DNA methylation traits, ensuring their independence (r^2^ < 0.01) by excluding palindromic single nucleotide polymorphisms (SNPs). Specifically, we identified 16 SNPs as proxies for a standard deviation increase in DNA methylation-estimated granulocyte proportions, 103 SNPs as proxies for a standard deviation increase in DNA methylation Hannum age acceleration, 14 SNPs as proxies for a standard deviation increase in DNA methylation-estimated plasminogen activator inhibitor-1 levels, 24 SNPs as proxies for a standard deviation increase in DNA methylation GrimAge acceleration and 98 SNPs as proxies for a standard deviation increase in DNA methylation PhenoAge acceleration, respectively. The F-statistics for the instrumental variables were all significantly above 10, indicating the absence of weak instrument bias (as shown in Table 1).

### 3.2 Causal effect of DNA methylation on cardiovascular disease risk

**Figure 2:**
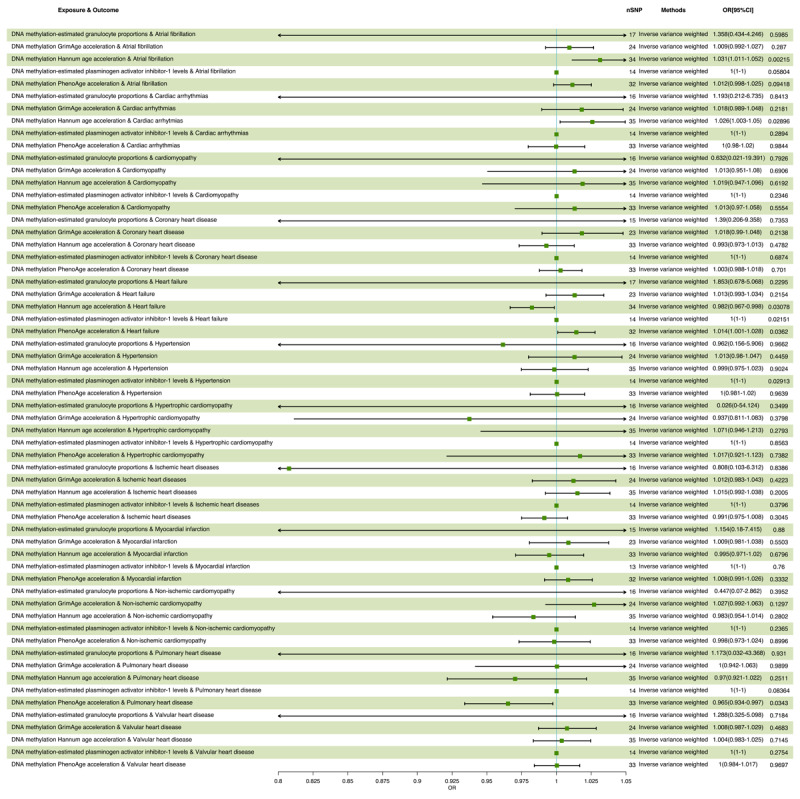
Results of MR analysis of the causal effect of each DNA methylation parameter on CVD.

The statistical results of MR are presented in Table 1. Using an IVW approach, we found that an SD increase in DNA methylation Hannum age acceleration exposure increased the risk of cardiac arrhythmias (OR = 1.026, 95% CI 1.003–1.049, *p* = 0.029) and atrial fibrillation (OR = 1.031, 95% CI 1.011–1.052, *p* = 0.002). The positive association between DNA methylation Hannum age acceleration exposure level and cardiac arrhythmias and atrial fibrillation risk were also observed in the maximum likelihood, MR-Egger, weighted median and weighted mode methods. Interestingly, our research found that DNA methylation Hannum age acceleration exposure was associated with decreased risk of heart failure (OR = 0.982, 95% CI 0.967–0.998, *p* = 0.031). Moreover, we found that an SD increased in DNA methylation PhenoAge acceleration exposure increased the risk of heart failure (OR = 1.014, 95% CI 1.001–1.028, *p* = 0.036). Further positive association between DNA methylation PhenoAge acceleration exposure levels and heart failure risk was also found in the maximum likelihood, MR-Egger, weighted median and weighted mode methods. DNA methylation PhenoAge acceleration was associated with decreased risk of pulmonary heart disease (OR = 0.965, 95% CI 0.934–0.997, *p* = 0.034). Exposure to DNA methylation-estimated granulocyte proportions was found to increase the risk of hypertension (OR = 1.0001, 95% CI 1.0000–1.0001, p = 0.029) (Figure 2).

### 3.3 Sensitivity analyses

Sensitivity analyses were shown in Supplementary Table 1. The estimates of causal effects obtained through the maximum likelihood, MR-Egger regression, weighted median method, and weighted model methods were consistent in terms of both magnitude and direction. This consistency strengthens the reliability of current findings. Our analysis provided no substantial evidence of horizontal pleiotropy in these results, suggesting that these IVs used in this study were not affected by other factors other than the exposure being investigated. Furthermore, the Cochran’s Q statistics, used to assess heterogeneity, did not indicate statistically significant differences among the estimates (p > 0.05), as showed in Supplementary Table 2. This suggested that the underlying genetic variants used as instruments for DNA methylation were not significantly influencing the outcome in different ways. and that the causal relationship between DNA methylation and CVD risk was not driven by single SNP (Supplementary Table 3). Overall, these findings indicated a consistent and reliable relationship between DNA methylation and CVD, with no significant confounding factors or outliers affecting the observed causal effects. The results of heterogeneity analysis and horizontal diversity analysis were shown in Supplementary Table 2 and Supplementary Table 3.

## 4 Discussion

We used MR method to systematically explore potential causal effects of DNA methylation susceptibility on 12 CVDs. We found a causal relationship between DNA methylation Hannum age acceleration and cardiac arrhythmias, atrial fibrillation. Exposed to high levels of DNA methylation Hannum age acceleration inhabitants with an increased risk of cardiac arrhythmias and atrial fibrillation compared to these inhabitants exposed to low concentrations levels of DNA methylation Hannum age acceleration. We also found that DNA methylation PhenoAge acceleration levels exposure levels were positively associated with the increased risk of developing heart failure. These results suggest a causal relationship between certain DNA methylation and CVD.

Previous reviews concluded that DNA methylation of inflammation-related genes has been related to risk for CVD. They propose that DNA methylation of stress-related and inflammation-related candidate genes partially mediates the associations of neighborhood environment and psychological stress with CVD [18,19,20]. Marquez et al. used an unsupervised machine-learning method (MOFA) to identify latent factors that capture biological and technical sources of variability in DNA methylation and gene expression datasets. By integrating these omic data, they identified three factors, almost exclusively explained by DNA methylation, that were independently associated with CVD [21]. In a multi-cohort study, they found that the blood epigenomic signature of CVD is complex and distinct across populations. DNA methylation may help identify individuals at higher risk of developing CVD if high-throughput biological information data is processed based on machine learning methods [22].

Large cohort studies have shown that circulating cell DNA methylation can predict future CVD risk in community populations [23,24,25]. Circulating cell DNA methylation was a novel biomarker for CVD [26,27,28,29]. The Hannum blood cell DNA clock is based on 71 CpGs, while Horvath’s clock, which is based on 353 individual CpG probes, allows the use of various tissues [30]. The DNA methylation Hannum age acceleration has a causal effect on atrial fibrillation, which has been confirmed in our study. One recent observational study showed that epigenetic age acceleration (Horvath, Hannum, DNA methylation [DNAm] PhenoAge, and DNAm GrimAge) were associated with a high incidence of atrial fibrillation [31]. Underlying these methylome changes is the ability of methyltransferase enzymes (DNMT1, DNMT2, DNMT3 A, and DNMT3B) to catalyze the reaction that transfers the methyl group to cytosine or adenosine [32]. Changes in gene methylation observed at different stages of AF development have been indicated as having a significant effect on the development of risk factors [33]. The higher the level of DNA methylation modification of some important genes (NPPA, NPRA, NPR-C, RASSF1 A) may be more likely to induce atrial fibrillation [33]. It has been shown that the development of diseases reorganizes the density of the methylome in the atria [34]. One genome-wide association studies loci with long-range chromatin conformation data identified a gene interaction network dominated by NKX2–5, TBX3, ZFHX3, and SYNPO2 L, which plays an important role in the pathogenesis of atrial fibrillation [35]. Furthermore, alterations in DNA methylation are associated with the onset and progression of cardiac fibrosis [36]. In recent years, fibrosis has been granted a causative role in heart diseases and is now emerging as a major contributor to atrial fibrillation (AF) pathogenesis [37,38]. The effect of DNA methylation on the pathogenesis of atrial fibrillation is regulated through multiple mechanisms, and the specific mechanism of action and targeted therapy based on this need to be further confirmed by future studies.

Our study also found DNA methylation PhenoAge acceleration has a causal relationship with an increased risk of heart failure. Many previous studies have found that DNA methylation modification is closely related to end-stage heart failure [39,40]. This research identified novel epigenetic interference of nuclear respiratory factor 1 via hypermethylation of its downstream promoter targets, further supporting a novel contribution of DNA methylation in the metabolic remodeling of heart failure [39,40]. In addition, the use of cardiomyotoxic drugs is an important risk factor for heart failure. Short-term administration of anthracyclines provokes long-lasting epigenetic modifications (DNA methylation) in cardiomyocytes both in vivo and in vitro, which explain in part the time lapse between the use of chemotherapy and the development of cardiotoxicity and, eventually, heart failure [41]. The pathological basis of heart failure is myocardial remodeling [42,43]. Recent genetic and biochemical analyses suggested that DNA methylation plays an important role in the occurrence and progression of myocardial remodeling. Therefore, regulation of DNA methylation or targeting its upstream signaling pathway may be a very promising treatment for heart failure. These also need to be further confirmed by subsequent research.

Our research has several advantages. First, MR analyses of genetic susceptibility to other factors and cardiovascular disease risk have recently been reported, but no MR studies have analyzed the potential causal relationship between DNA methylation and each CVD. Secondly, through large-scale GWAS analysis, we conducted MR analyses for five DNA methylation and 12 cardiovascular diseases. The MR design reinforced the causal inference by diminishing residual confounding and other biases. Genetic knowledge of DNA methylation exposure and cardiovascular-related diseases has been further expanded. These large-scale geographic information systems provide more precise correlation. This Mendelian randomization analysis utilizes the latest exposure and outcome GIS datasets to comprehensively investigate the potential relationship between DNA methylation and cardiovascular disease, avoiding traditional confounding factors and reverse causation. Repeated analysis was done using a variety of methods to obtain consistent results. Sensitivity analysis and IVS intensity evaluation were used to verify that the results were not biased. We confined the population in the present study to individuals of European ancestry to minimize population structure bias, with the exception for the analysis for coronary artery disease, which might be challenged by bias from ethnicity, based on consortium data where European individuals comprised over 80% of participants. Nevertheless, this population confinement limited the generalizability of our findings to other populations.

## 5 Conclusions

In conclusion, this two-sample MR Study found a causal relationship between DNA methylation and CVD. To be specific, inhabitants exposed to high levels of DNA methylation Hannum age acceleration had an increased risk of cardiac arrhythmias and atrial fibrillation. DNA methylation PhenoAge acceleration levels exposure levels were positively associated with the increased risk of developing heart failure. This may help to identify individuals at high risk of CVD by DNA methylation, beyond the risk factors traditionally associated with CVD. This has important implications for the prevention of cardiovascular diseases.

## Data Accessibility Statement

The data were derived from the following resources available in the public domain: Telomere GWAS.

## Additional File

The additional file for this article can be found as follows:

10.5334/gh.1324.s1 Supplementary Tables.

## Abbreviations

MR: Mendelian randomization; CVD: cardiovascular disease; IVW: inverse variance weighted; GWAS: genome-wide association studies; SNPS: single-nucleotide polymorphisms
