# Supplementary material for: Causal Associations of DNA Methylation and Cardiovascular Disease: A Two-Sample Mendelian Randomization Study
Source: Glob Heart. 2024 May 14;19(1):48. doi: 10.5334/gh.1324 (PMC11100526; doi:10.5334/gh.1324)
Supplement: Supplementary Tables. [file gh-19-1-1324-s1.pdf]

	Outcome	Method	No. of snp	Beta	se	p-value	OR	95%CI	
DNA metl	Atrial fibri	Maximum	17	0.325	0.4548	0.4748	1.3840	0.5676	3.3750
DNA metl	Atrial fibri	MR Egger	17	0.756	1.803	0.681	2.1297	0.0622	72.9577
DNA metl	Atrial fibri	Weighted	17	0.1963	0.6927	0.7769	1.2169	0.3131	4.7303
DNA metl	Atrial fibri	Inverse va	17	0.3062	0.5815	0.5985	1.3583	0.4345	4.2458
DNA metl	Atrial fibri	Simple mo	17	-0.1838	1.428	0.8992	0.8321	0.0507	13.6683
DNA metl	Atrial fibri	Weighted	17	1.281	1.197	0.3004	3.6002	0.3447	37.6044
DNA metl	Atrial fibri	Maximum	24	0.00947	0.00886	0.2849	1.0095	0.9921	1.0272
DNA metl	Atrial fibri	MR Egger	24	-0.0115	0.02534	0.6532	0.9885	0.9406	1.0389
DNA metl	Atrial fibri	Weighted	24	0.01062	0.01178	0.3676	1.0107	0.9876	1.0343
DNA metl	Atrial fibri	Inverse va	24	0.00929	0.00872	0.287	1.0093	0.9922	1.0267
DNA metl	Atrial fibri	Simple mo	24	0.0149	0.02459	0.5505	1.0150	0.9673	1.0651
DNA metl	Atrial fibri	Weighted	24	0.01067	0.02207	0.6332	1.0107	0.9679	1.0554
DNA metl	Atrial fibri	Maximum	34	0.03122	0.00719	1.4E-05	1.0317	1.0173	1.0464
DNA metl	Atrial fibri	MR Egger	34	0.02291	0.02846	0.4268	1.0232	0.9677	1.0819
DNA metl	Atrial fibri	Weighted	34	0.01482	0.01072	0.1671	1.0149	0.9938	1.0365
DNA metl	Atrial fibri	Inverse va	34	0.03087	0.01006	0.00215	1.0314	1.0112	1.0519
DNA metl	Atrial fibri	Simple mo	34	0.00745	0.01886	0.6953	1.0075	0.9709	1.0454
DNA metl	Atrial fibri	Weighted	34	0.01514	0.01651	0.3657	1.0153	0.9829	1.0486
DNA metl	Atrial fibri	Maximum	14	-2E-05	1.3E-05	0.05601	1.0000	0.9999	1.0000
DNA metl	Atrial fibri	MR Egger	14	-3E-05	1.5E-05	0.05602	1.0000	0.9999	1.0000
DNA metl	Atrial fibri	Weighted	14	-3E-05	1.7E-05	0.1244	1.0000	0.9999	1.0000
DNA metl	Atrial fibri	Inverse va	14	-2E-05	1.3E-05	0.05804	1.0000	1.0000	1.0000
DNA metl	Atrial fibri	Simple mo	14	-2E-05	3.3E-05	0.529	1.0000	0.9999	1.0000
DNA metl	Atrial fibri	Weighted	14	-2E-05	3E-05	0.4922	1.0000	0.9999	1.0000
DNA metl	Atrial fibri	Maximum	32	0.01188	0.00547	0.02993	1.0120	1.0012	1.0229
DNA metl	Atrial fibri	MR Egger	32	0.0093	0.01547	0.5523	1.0093	0.9792	1.0404
DNA metl	Atrial fibri	Weighted	32	0.01004	0.00788	0.2029	1.0101	0.9946	1.0258
DNA metl	Atrial fibri	Inverse va	32	0.01146	0.00685	0.09418	1.0115	0.9980	1.0252
DNA metl	Atrial fibri	Simple mo	32	0.01283	0.01514	0.4034	1.0129	0.9833	1.0434
DNA metl	Atrial fibri	Weighted	32	0.01057	0.01318	0.4284	1.0106	0.9849	1.0371
DNA metl	Cardiac ar	Maximum	16	0.1793	0.7155	0.8021	1.1964	0.2943	4.8631
DNA metl	Cardiac ar	MR Egger	16	-2.867	2.38	0.2483	0.0569	0.0005	6.0364
DNA metl	Cardiac ar	Weighted	16	1.208	1.028	0.2403	3.3468	0.4462	25.1003
DNA metl	Cardiac ar	Inverse va	16	0.1768	0.8829	0.8413	1.1934	0.2115	6.7348
DNA metl	Cardiac ar	Simple mo	16	-1.955	2.388	0.4259	0.1416	0.0013	15.2637
DNA metl	Cardiac ar	Weighted	16	2.755	1.824	0.1518	15.7210	0.4404	#######
DNA metl	Cardiac ar	Maximum	24	0.01867	0.01318	0.1566	1.0188	0.9929	1.0455
DNA metl	Cardiac ar	MR Egger	24	0.00729	0.03457	0.8348	1.0073	0.9413	1.0779
DNA metl	Cardiac ar	Weighted	24	0.01897	0.01993	0.3412	1.0192	0.9801	1.0597
DNA metl	Cardiac ar	Inverse va	24	0.01808	0.01468	0.2181	1.0182	0.9894	1.0480
DNA metl	Cardiac ar	Simple mo	24	0.03798	0.03389	0.2739	1.0387	0.9720	1.1100

DNA metl	Cardiac ar	Weighted	24	0.02124	0.02912	0.4731	1.0215	0.9648	1.0815
DNA metl	Cardiac ar	Maximum	35	0.02619	0.01149	0.0226	1.0265	1.0037	1.0499
DNA metl	Cardiac ar	MR Egger	35	0.04175	0.0359	0.2532	1.0426	0.9718	1.1186
DNA metl	Cardiac ar	Weighted	35	0.03349	0.01682	0.04655	1.0341	1.0005	1.0687
DNA metl	Cardiac ar	Inverse va	35	0.02545	0.01165	0.02896	1.0258	1.0026	1.0495
DNA metl	Cardiac ar	Simple mo	35	0.05472	0.03208	0.09719	1.0562	0.9919	1.1248
DNA metl	Cardiac ar	Weighted	35	0.03134	0.02798	0.2705	1.0318	0.9768	1.0900
DNA metl	Cardiac ar	Maximum	14	2.5E-05	2.2E-05	0.2661	1.0000	1.0000	1.0001
DNA metl	Cardiac ar	MR Egger	14	1.5E-05	2.8E-05	0.5951	1.0000	1.0000	1.0001
DNA metl	Cardiac ar	Weighted	14	5.5E-05	3E-05	0.06991	1.0001	1.0000	1.0001
DNA metl	Cardiac ar	Inverse va	14	2.4E-05	2.3E-05	0.2894	1.0000	1.0000	1.0001
DNA metl	Cardiac ar	Simple mo	14	7.9E-05	5.6E-05	0.1827	1.0001	1.0000	1.0002
DNA metl	Cardiac ar	Weighted	14	7.9E-05	5E-05	0.1399	1.0001	1.0000	1.0002
DNA metl	Cardiac ar	Maximum	33	-0.0002	0.00847	0.9801	0.9998	0.9833	1.0165
DNA metl	Cardiac ar	MR Egger	33	-0.0302	0.02352	0.208	0.9702	0.9265	1.0160
DNA metl	Cardiac ar	Weighted	33	0.00222	0.0131	0.8654	1.0022	0.9768	1.0283
DNA metl	Cardiac ar	Inverse va	33	-0.0002	0.01037	0.9844	0.9998	0.9797	1.0203
DNA metl	Cardiac ar	Simple mo	33	0.01657	0.03177	0.6056	1.0167	0.9553	1.0820
DNA metl	Cardiac ar	Weighted	33	0.01293	0.02924	0.6615	1.0130	0.9566	1.0728
DNA metl	Cardiomy	Maximum	16	-0.4422	1.77	0.8027	0.6426	0.0200	20.6352
DNA metl	Cardiomy	MR Egger	16	-5.656	4.839	0.2619	0.0035	0.0000	45.9907
DNA metl	Cardiomy	Weighted	16	-0.9384	2.345	0.689	0.3913	0.0039	38.7759
DNA metl	Cardiomy	Inverse va	16	-0.4593	1.747	0.7926	0.6317	0.0206	19.3912
DNA metl	Cardiomy	Simple mo	16	-1.167	3.816	0.7638	0.3113	0.0002	#######
DNA metl	Cardiomy	Weighted	16	-0.944	3.505	0.7913	0.3891	0.0004	#######
DNA metl	Cardiomy	Maximum	24	0.01305	0.0331	0.6934	1.0131	0.9495	1.0810
DNA metl	Cardiomy	MR Egger	24	-0.0528	0.075	0.4892	0.9486	0.8189	1.0988
DNA metl	Cardiomy	Weighted	24	-0.0057	0.04798	0.905	0.9943	0.9050	1.0923
DNA metl	Cardiomy	Inverse va	24	0.01294	0.03252	0.6906	1.0130	0.9505	1.0797
DNA metl	Cardiomy	Simple mo	24	0.08271	0.09047	0.3701	1.0862	0.9097	1.2970
DNA metl	Cardiomy	Weighted	24	0.02501	0.06362	0.6979	1.0253	0.9051	1.1615
DNA metl	Cardiomy	Maximum	35	0.0197	0.02928	0.5012	1.0199	0.9630	1.0801
DNA metl	Cardiomy	MR Egger	35	0.01694	0.1155	0.8843	1.0171	0.8110	1.2755
DNA metl	Cardiomy	Weighted	35	0.02466	0.04463	0.5806	1.0250	0.9391	1.1187
DNA metl	Cardiomy	Inverse va	35	0.01859	0.0374	0.6192	1.0188	0.9468	1.0962
DNA metl	Cardiomy	Simple mo	35	-0.0148	0.09414	0.8757	0.9853	0.8193	1.1849
DNA metl	Cardiomy	Weighted	35	0.04495	0.07569	0.5565	1.0460	0.9018	1.2132
DNA metl	Cardiomy	Maximum	14	8.3E-05	5.7E-05	0.1457	1.0001	1.0000	1.0002
DNA metl	Cardiomy	MR Egger	14	0.00013	8.4E-05	0.1621	1.0001	1.0000	1.0003
DNA metl	Cardiomy	Weighted	14	4E-05	8.2E-05	0.6246	1.0000	0.9999	1.0002
DNA metl	Cardiomy	Inverse va	14	8.3E-05	7E-05	0.2346	1.0001	0.9999	1.0002
DNA metl	Cardiomy	Simple mo	14	0.00013	0.00015	0.4204	1.0001	0.9998	1.0004

DNA metl	Cardiomy	Weighted	14	0.00013	0.00014	0.393	1.0001	0.9998	1.0004
DNA metl	Cardiomy	Maximum	33	0.01322	0.02103	0.5295	1.0133	0.9724	1.0559
DNA metl	Cardiomy	MR Egger	33	0.08461	0.04975	0.09899	1.0883	0.9872	1.1998
DNA metl	Cardiomy	Weighted	33	0.02886	0.03123	0.3555	1.0293	0.9682	1.0943
DNA metl	Cardiomy	Inverse va	33	0.01304	0.02211	0.5554	1.0131	0.9702	1.0580
DNA metl	Cardiomy	Simple mo	33	0.05182	0.06383	0.4229	1.0532	0.9293	1.1935
DNA metl	Cardiomy	Weighted	33	0.04073	0.05687	0.4791	1.0416	0.9317	1.1644
DNA metl	Coronary	Maximum	15	0.3526	0.7018	0.6153	1.4228	0.3595	5.6301
DNA metl	Coronary	MR Egger	15	1.626	3.304	0.6307	5.0835	0.0078	#######
DNA metl	Coronary	Weighted	15	0.8202	1.056	0.4374	2.2710	0.2866	17.9926
DNA metl	Coronary	Inverse va	15	0.329	0.9731	0.7353	1.3896	0.2063	9.3584
DNA metl	Coronary	Simple mo	15	0.5359	1.931	0.7854	1.7090	0.0388	75.2383
DNA metl	Coronary	Weighted	15	1.127	1.773	0.5352	3.0864	0.0956	99.6915
DNA metl	Coronary	Maximum	23	0.01861	0.01334	0.1628	1.0188	0.9925	1.0458
DNA metl	Coronary	MR Egger	23	0.02551	0.04499	0.5767	1.0258	0.9393	1.1204
DNA metl	Coronary	Weighted	23	0.00375	0.01967	0.8487	1.0038	0.9658	1.0432
DNA metl	Coronary	Inverse va	23	0.01817	0.01461	0.2138	1.0183	0.9896	1.0479
DNA metl	Coronary	Simple mo	23	-0.0118	0.03667	0.75	0.9882	0.9197	1.0619
DNA metl	Coronary	Weighted	23	-0.0136	0.03467	0.6992	0.9865	0.9217	1.0559
DNA metl	Coronary	Maximum	33	-0.0075	0.00979	0.445	0.9925	0.9737	1.0118
DNA metl	Coronary	MR Egger	33	0.02271	0.0274	0.4136	1.0230	0.9695	1.0794
DNA metl	Coronary	Weighted	33	-0.0061	0.01349	0.649	0.9939	0.9679	1.0205
DNA metl	Coronary	Inverse va	33	-0.0072	0.01019	0.4782	0.9928	0.9732	1.0128
DNA metl	Coronary	Simple mo	33	-0.0224	0.02433	0.3646	0.9779	0.9323	1.0256
DNA metl	Coronary	Weighted	33	-0.0075	0.01952	0.7049	0.9926	0.9553	1.0313
DNA metl	Coronary	Maximum	14	-8E-06	1.7E-05	0.6144	1.0000	1.0000	1.0000
DNA metl	Coronary	MR Egger	14	7.7E-06	2.2E-05	0.7316	1.0000	1.0000	1.0001
DNA metl	Coronary	Weighted	14	-6E-06	2.2E-05	0.7976	1.0000	1.0000	1.0000
DNA metl	Coronary	Inverse va	14	-8E-06	2.1E-05	0.6874	1.0000	1.0000	1.0000
DNA metl	Coronary	Simple mo	14	-4E-05	5.3E-05	0.4232	1.0000	0.9999	1.0001
DNA metl	Coronary	Weighted	14	-4E-05	4.4E-05	0.3422	1.0000	0.9999	1.0000
DNA metl	Coronary	Maximum	33	0.00311	0.00733	0.672	1.0031	0.9888	1.0176
DNA metl	Coronary	MR Egger	33	-0.0121	0.01746	0.4929	0.9880	0.9547	1.0223
DNA metl	Coronary	Weighted	33	-0.0112	0.01051	0.2851	0.9888	0.9687	1.0094
DNA metl	Coronary	Inverse va	33	0.00299	0.0078	0.701	1.0030	0.9878	1.0184
DNA metl	Coronary	Simple mo	33	-0.0226	0.01877	0.2378	0.9777	0.9424	1.0143
DNA metl	Coronary	Weighted	33	-0.0154	0.01459	0.2979	0.9847	0.9569	1.0132
DNA metl	Heart failu	Maximum	17	0.6432	0.5225	0.2184	1.9026	0.6832	5.2978
DNA metl	Heart failu	MR Egger	17	0.8294	1.543	0.5987	2.2919	0.1114	47.1663
DNA metl	Heart failu	Weighted	17	0.3301	0.7127	0.6432	1.3911	0.3441	5.6237
DNA metl	Heart failu	Inverse va	17	0.6168	0.5133	0.2295	1.8530	0.6776	5.0676
DNA metl	Heart failu	Simple mo	17	0.4675	1.354	0.7343	1.5960	0.1123	22.6767

DNA metl	Heart failı	Weighted	17	0.4409	1.182	0.7141	1.5541	0.1532	15.7623
DNA metl	Heart failı	Maximum	23	0.01321	0.01066	0.2151	1.0133	0.9923	1.0347
DNA metl	Heart failı	MR Egger	23	0.02277	0.03396	0.5099	1.0230	0.9572	1.0934
DNA metl	Heart failı	Weighted	23	0.00552	0.01483	0.7096	1.0055	0.9767	1.0352
DNA metl	Heart failı	Inverse va	23	0.01298	0.01048	0.2154	1.0131	0.9925	1.0341
DNA metl	Heart failı	Simple mc	23	0.00498	0.02495	0.8436	1.0050	0.9570	1.0554
DNA metl	Heart failı	Weighted	23	0.00305	0.02547	0.9057	1.0031	0.9542	1.0544
DNA metl	Heart failı	Maximum	34	-0.0179	0.00835	0.03184	0.9822	0.9663	0.9984
DNA metl	Heart failı	MR Egger	34	-0.0058	0.02511	0.8178	0.9942	0.9464	1.0443
DNA metl	Heart failı	Weighted	34	-0.0127	0.01196	0.2874	0.9874	0.9645	1.0108
DNA metl	Heart failı	Inverse va	34	-0.0177	0.00821	0.03078	0.9824	0.9667	0.9984
DNA metl	Heart failı	Simple mc	34	-0.0026	0.02501	0.9164	0.9974	0.9496	1.0475
DNA metl	Heart failı	Weighted	34	-0.0096	0.02312	0.6821	0.9905	0.9466	1.0364
DNA metl	Heart failı	Maximum	14	-4E-05	1.7E-05	0.01523	1.0000	0.9999	1.0000
DNA metl	Heart failı	MR Egger	14	-5E-05	2E-05	0.02415	0.9999	0.9999	1.0000
DNA metl	Heart failı	Weighted	14	-5E-05	2.2E-05	0.01598	0.9999	0.9999	1.0000
DNA metl	Heart failı	Inverse va	14	-4E-05	1.7E-05	0.02151	1.0000	0.9999	1.0000
DNA metl	Heart failı	Simple mc	14	-4E-06	4.3E-05	0.9278	1.0000	0.9999	1.0001
DNA metl	Heart failı	Weighted	14	-8E-05	4E-05	0.0551	0.9999	0.9998	1.0000
DNA metl	Heart failı	Maximum	32	0.01443	0.00646	0.02561	1.0145	1.0018	1.0275
DNA metl	Heart failı	MR Egger	32	0.01115	0.01666	0.5084	1.0112	0.9787	1.0448
DNA metl	Heart failı	Weighted	32	0.01937	0.0087	0.02607	1.0196	1.0023	1.0371
DNA metl	Heart failı	Inverse va	32	0.01416	0.00676	0.0362	1.0143	1.0009	1.0278
DNA metl	Heart failı	Simple mc	32	0.02335	0.01849	0.2159	1.0236	0.9872	1.0614
DNA metl	Heart failı	Weighted	32	0.02237	0.01413	0.1235	1.0226	0.9947	1.0513
DNA metl	Hypertens	Maximum	16	-0.0378	0.6173	0.9512	0.9629	0.2872	3.2289
DNA metl	Hypertens	MR Egger	16	-1.581	2.619	0.5557	0.2058	0.0012	34.8914
DNA metl	Hypertens	Weighted	16	0.6547	0.9006	0.4672	1.9246	0.3294	11.2445
DNA metl	Hypertens	Inverse va	16	-0.0392	0.9261	0.9662	0.9615	0.1565	5.9057
DNA metl	Hypertens	Simple mc	16	-3.123	1.982	0.136	0.0440	0.0009	2.1420
DNA metl	Hypertens	Weighted	16	0.4381	1.371	0.7537	1.5498	0.1055	22.7658
DNA metl	Hypertens	Maximum	24	0.01369	0.01142	0.2307	1.0138	0.9913	1.0367
DNA metl	Hypertens	MR Egger	24	0.01291	0.03997	0.7497	1.0130	0.9367	1.0955
DNA metl	Hypertens	Weighted	24	0.00564	0.0172	0.7432	1.0057	0.9723	1.0401
DNA metl	Hypertens	Inverse va	24	0.0129	0.01692	0.4459	1.0130	0.9799	1.0471
DNA metl	Hypertens	Simple mc	24	-0.0151	0.02949	0.6132	0.9850	0.9297	1.0436
DNA metl	Hypertens	Weighted	24	-0.0008	0.02131	0.9704	0.9992	0.9583	1.0418
DNA metl	Hypertens	Maximum	35	-0.0016	0.00988	0.8719	0.9984	0.9793	1.0179
DNA metl	Hypertens	MR Egger	35	-0.0099	0.03791	0.7958	0.9902	0.9193	1.0665
DNA metl	Hypertens	Weighted	35	-0.0028	0.01464	0.846	0.9972	0.9690	1.0262
DNA metl	Hypertens	Inverse va	35	-0.0015	0.01227	0.9024	0.9985	0.9748	1.0228
DNA metl	Hypertens	Simple mc	35	0.00123	0.02854	0.966	1.0012	0.9468	1.0588

DNA metl	Hypertens	Weighted	35	-0.0013	0.02318	0.9571	0.9987	0.9544	1.0452
DNA metl	Hypertens	Maximum	14	5.7E-05	1.9E-05	0.00312	1.0001	1.0000	1.0001
DNA metl	Hypertens	MR Egger	14	4.8E-05	3.1E-05	0.1507	1.0000	1.0000	1.0001
DNA metl	Hypertens	Weighted	14	4.5E-05	2.6E-05	0.09264	1.0000	1.0000	1.0001
DNA metl	Hypertens	Inverse va	14	5.6E-05	2.6E-05	0.02913	1.0001	1.0000	1.0001
DNA metl	Hypertens	Simple mo	14	0.00015	7.4E-05	0.07014	1.0001	1.0000	1.0003
DNA metl	Hypertens	Weighted	14	-5E-05	7.1E-05	0.506	1.0000	0.9998	1.0001
DNA metl	Hypertens	Maximum	33	0.0005	0.00738	0.9459	1.0005	0.9861	1.0151
DNA metl	Hypertens	MR Egger	33	-0.0156	0.02318	0.5067	0.9846	0.9408	1.0303
DNA metl	Hypertens	Weighted	33	-0.005	0.01147	0.6614	0.9950	0.9729	1.0176
DNA metl	Hypertens	Inverse va	33	0.00045	0.00999	0.9639	1.0005	0.9811	1.0202
DNA metl	Hypertens	Simple mo	33	-0.0072	0.02245	0.7503	0.9928	0.9501	1.0375
DNA metl	Hypertens	Weighted	33	-0.0064	0.01854	0.7328	0.9936	0.9582	1.0304
DNA metl	Hypertrop	Maximum	16	-3.539	3.935	0.3684	0.0290	0.0000	64.9488
DNA metl	Hypertrop	MR Egger	16	-17.67	10.82	0.1245	0.0000	0.0000	34.3705
DNA metl	Hypertrop	Weighted	16	-4.095	5.405	0.4487	0.0167	0.0000	#######
DNA metl	Hypertrop	Inverse va	16	-3.639	3.893	0.3499	0.0263	0.0000	54.1241
DNA metl	Hypertrop	Simple mo	16	-2.213	9.434	0.8177	0.1094	0.0000	#######
DNA metl	Hypertrop	Weighted	16	-3.346	7.675	0.6691	0.0352	0.0000	#######
DNA metl	Hypertrop	Maximum	24	-0.0668	0.07361	0.364	0.9354	0.8097	1.0805
DNA metl	Hypertrop	MR Egger	24	-0.1067	0.1724	0.5421	0.8988	0.6411	1.2601
DNA metl	Hypertrop	Weighted	24	-0.0593	0.1045	0.5704	0.9424	0.7679	1.1566
DNA metl	Hypertrop	Inverse va	24	-0.0647	0.07362	0.3798	0.9374	0.8114	1.0829
DNA metl	Hypertrop	Simple mo	24	-0.0877	0.1839	0.6379	0.9160	0.6388	1.3136
DNA metl	Hypertrop	Weighted	24	-0.057	0.1415	0.6906	0.9446	0.7158	1.2465
DNA metl	Hypertrop	Maximum	35	0.07145	0.06463	0.2689	1.0741	0.9463	1.2191
DNA metl	Hypertrop	MR Egger	35	0.08497	0.1935	0.6634	1.0887	0.7451	1.5908
DNA metl	Hypertrop	Weighted	35	0.0035	0.09611	0.9709	1.0035	0.8312	1.2115
DNA metl	Hypertrop	Inverse va	35	0.06875	0.06354	0.2793	1.0712	0.9457	1.2132
DNA metl	Hypertrop	Simple mo	35	-0.0631	0.205	0.76	0.9388	0.6282	1.4031
DNA metl	Hypertrop	Weighted	35	-0.0584	0.1613	0.7195	0.9433	0.6876	1.2940
DNA metl	Hypertrop	Maximum	14	2.3E-05	0.00013	0.8553	1.0000	0.9998	1.0003
DNA metl	Hypertrop	MR Egger	14	1.1E-05	0.00015	0.9411	1.0000	0.9997	1.0003
DNA metl	Hypertrop	Weighted	14	1.5E-05	0.00016	0.9268	1.0000	0.9997	1.0003
DNA metl	Hypertrop	Inverse va	14	2.3E-05	0.00012	0.8563	1.0000	0.9998	1.0003
DNA metl	Hypertrop	Simple mo	14	-8E-05	0.00032	0.8078	0.9999	0.9993	1.0005
DNA metl	Hypertrop	Weighted	14	-8E-05	0.00029	0.7869	0.9999	0.9994	1.0005
DNA metl	Hypertrop	Maximum	33	0.01743	0.04696	0.7104	1.0176	0.9281	1.1157
DNA metl	Hypertrop	MR Egger	33	0.09484	0.117	0.4239	1.0995	0.8742	1.3829
DNA metl	Hypertrop	Weighted	33	0.1037	0.06947	0.1353	1.1093	0.9681	1.2711
DNA metl	Hypertrop	Inverse va	33	0.01688	0.05049	0.7382	1.0170	0.9212	1.1228
DNA metl	Hypertrop	Simple mo	33	0.1396	0.1299	0.2902	1.1498	0.8914	1.4832

DNA metl	Hypertrop	Weighted	33	0.1434	0.1074	0.1915	1.1542	0.9351	1.4246
DNA metl	Ischemic l	Maximum	16	-0.2066	0.7417	0.7806	0.8133	0.1901	3.4803
DNA metl	Ischemic l	MR Egger	16	-0.005	3.007	0.9987	0.9950	0.0027	#######
DNA metl	Ischemic l	Weighted	16	-1.571	1.137	0.167	0.2078	0.0224	1.9300
DNA metl	Ischemic l	Inverse va	16	-0.2136	1.049	0.8386	0.8077	0.1033	6.3119
DNA metl	Ischemic l	Simple mo	16	-2.564	1.979	0.2148	0.0770	0.0016	3.7242
DNA metl	Ischemic l	Weighted	16	-2.453	2.075	0.2555	0.0860	0.0015	5.0229
DNA metl	Ischemic l	Maximum	24	0.01253	0.01357	0.3559	1.0126	0.9860	1.0399
DNA metl	Ischemic l	MR Egger	24	0.03028	0.03555	0.4035	1.0307	0.9614	1.1051
DNA metl	Ischemic l	Weighted	24	0.02495	0.01943	0.1991	1.0253	0.9870	1.0651
DNA metl	Ischemic l	Inverse va	24	0.01216	0.01515	0.4223	1.0122	0.9826	1.0427
DNA metl	Ischemic l	Simple mo	24	0.00616	0.03346	0.8555	1.0062	0.9423	1.0744
DNA metl	Ischemic l	Weighted	24	0.01773	0.02302	0.4489	1.0179	0.9730	1.0649
DNA metl	Ischemic l	Maximum	35	0.01506	0.01184	0.2035	1.0152	0.9919	1.0390
DNA metl	Ischemic l	MR Egger	35	0.01262	0.0358	0.7266	1.0127	0.9441	1.0863
DNA metl	Ischemic l	Weighted	35	0.02863	0.01671	0.08669	1.0290	0.9959	1.0633
DNA metl	Ischemic l	Inverse va	35	0.01494	0.01167	0.2005	1.0151	0.9921	1.0385
DNA metl	Ischemic l	Simple mo	35	0.02793	0.03489	0.429	1.0283	0.9604	1.1011
DNA metl	Ischemic l	Weighted	35	0.03497	0.03114	0.2693	1.0356	0.9743	1.1008
DNA metl	Ischemic l	Maximum	14	2.3E-05	2.3E-05	0.3231	1.0000	1.0000	1.0001
DNA metl	Ischemic l	MR Egger	14	-2E-06	2.8E-05	0.9436	1.0000	0.9999	1.0001
DNA metl	Ischemic l	Weighted	14	2E-05	3.1E-05	0.5132	1.0000	1.0000	1.0001
DNA metl	Ischemic l	Inverse va	14	2.2E-05	2.5E-05	0.3796	1.0000	1.0000	1.0001
DNA metl	Ischemic l	Simple mo	14	4.9E-05	8E-05	0.5506	1.0000	0.9999	1.0002
DNA metl	Ischemic l	Weighted	14	4.9E-05	8.2E-05	0.5576	1.0000	0.9999	1.0002
DNA metl	Ischemic l	Maximum	33	-0.0089	0.00856	0.3009	0.9912	0.9747	1.0080
DNA metl	Ischemic l	MR Egger	33	-0.0309	0.01958	0.1246	0.9696	0.9331	1.0075
DNA metl	Ischemic l	Weighted	33	-0.0104	0.01138	0.3624	0.9897	0.9679	1.0120
DNA metl	Ischemic l	Inverse va	33	-0.0087	0.00849	0.3045	0.9913	0.9750	1.0080
DNA metl	Ischemic l	Simple mo	33	-0.0053	0.02246	0.8147	0.9947	0.9519	1.0395
DNA metl	Ischemic l	Weighted	33	-0.0095	0.01915	0.6233	0.9905	0.9541	1.0284
DNA metl	Myocardia	Maximum	15	0.1536	0.7692	0.8417	1.1660	0.2582	5.2658
DNA metl	Myocardia	MR Egger	15	2.66	3.133	0.4112	14.2963	0.0308	#######
DNA metl	Myocardia	Weighted	15	0.9916	1.149	0.3881	2.6955	0.2835	25.6268
DNA metl	Myocardia	Inverse va	15	0.1433	0.9491	0.88	1.1541	0.1796	7.4152
DNA metl	Myocardia	Simple mo	15	1.449	2.007	0.4822	4.2589	0.0833	#######
DNA metl	Myocardia	Weighted	15	1.318	1.653	0.4386	3.7359	0.1463	95.3811
DNA metl	Myocardia	Maximum	23	0.00881	0.01463	0.5474	1.0088	0.9803	1.0382
DNA metl	Myocardia	MR Egger	23	0.02615	0.04354	0.5546	1.0265	0.9425	1.1179
DNA metl	Myocardia	Weighted	23	-0.0012	0.02015	0.9542	0.9988	0.9602	1.0391
DNA metl	Myocardia	Inverse va	23	0.00859	0.01438	0.5503	1.0086	0.9806	1.0375
DNA metl	Myocardia	Simple mo	23	-0.004	0.03748	0.915	0.9960	0.9254	1.0719

DNA metl	Myocardi	Weighted	23	-0.0067	0.03617	0.8545	0.9933	0.9253	1.0663
DNA metl	Myocardi	Maximum	33	-0.0054	0.01096	0.622	0.9946	0.9735	1.0162
DNA metl	Myocardi	MR Egger	33	-0.0018	0.03461	0.9599	0.9982	0.9328	1.0683
DNA metl	Myocardi	Weighted	33	-0.0035	0.01593	0.8269	0.9965	0.9659	1.0281
DNA metl	Myocardi	Inverse va	33	-0.0052	0.01253	0.6796	0.9948	0.9707	1.0196
DNA metl	Myocardi	Simple mo	33	-0.0044	0.02931	0.8817	0.9956	0.9400	1.0545
DNA metl	Myocardi	Weighted	33	-0.0008	0.02426	0.9738	0.9992	0.9528	1.0479
DNA metl	Myocardi	Maximum	13	-7E-06	1.9E-05	0.7268	1.0000	1.0000	1.0000
DNA metl	Myocardi	MR Egger	13	1.2E-05	2.2E-05	0.5894	1.0000	1.0000	1.0001
DNA metl	Myocardi	Weighted	13	-5E-06	2.4E-05	0.8193	1.0000	0.9999	1.0000
DNA metl	Myocardi	Inverse va	13	-7E-06	2.1E-05	0.76	1.0000	1.0000	1.0000
DNA metl	Myocardi	Simple mo	13	-6E-05	5.2E-05	0.2768	0.9999	0.9998	1.0000
DNA metl	Myocardi	Weighted	13	2.3E-05	4.6E-05	0.6235	1.0000	0.9999	1.0001
DNA metl	Myocardi	Maximum	32	0.00833	0.00822	0.3108	1.0084	0.9923	1.0247
DNA metl	Myocardi	MR Egger	32	-0.0081	0.01976	0.6838	0.9919	0.9542	1.0311
DNA metl	Myocardi	Weighted	32	0.00473	0.01226	0.6998	1.0047	0.9809	1.0292
DNA metl	Myocardi	Inverse va	32	0.00841	0.0087	0.3332	1.0084	0.9914	1.0258
DNA metl	Myocardi	Simple mo	32	0.00617	0.02209	0.7819	1.0062	0.9636	1.0507
DNA metl	Myocardi	Weighted	32	0.00617	0.01855	0.7417	1.0062	0.9703	1.0434
DNA metl	Non-ische	Maximum	16	-0.7855	0.96	0.4132	0.4559	0.0695	2.9925
DNA metl	Non-ische	MR Egger	16	0.3284	2.624	0.9022	1.3887	0.0081	#######
DNA metl	Non-ische	Weighted	16	-0.4243	1.325	0.7489	0.6542	0.0487	8.7820
DNA metl	Non-ische	Inverse va	16	-0.8057	0.9476	0.3952	0.4468	0.0697	2.8622
DNA metl	Non-ische	Simple mo	16	-0.4116	2.522	0.8725	0.6626	0.0047	92.8997
DNA metl	Non-ische	Weighted	16	-0.0041	2.32	0.9986	0.9959	0.0106	93.9855
DNA metl	Non-ische	Maximum	24	0.02693	0.01807	0.1362	1.0273	0.9915	1.0643
DNA metl	Non-ische	MR Egger	24	-0.016	0.04065	0.6983	0.9842	0.9088	1.0658
DNA metl	Non-ische	Weighted	24	0.02784	0.02413	0.2485	1.0282	0.9807	1.0780
DNA metl	Non-ische	Inverse va	24	0.02671	0.01763	0.1297	1.0271	0.9922	1.0632
DNA metl	Non-ische	Simple mo	24	0.051	0.04525	0.2713	1.0523	0.9630	1.1499
DNA metl	Non-ische	Weighted	24	-0.0071	0.03601	0.8458	0.9929	0.9253	1.0656
DNA metl	Non-ische	Maximum	35	-0.017	0.01569	0.278	0.9831	0.9534	1.0138
DNA metl	Non-ische	MR Egger	35	0.02638	0.047	0.5784	1.0267	0.9364	1.1258
DNA metl	Non-ische	Weighted	35	-0.0286	0.02227	0.1987	0.9718	0.9303	1.0151
DNA metl	Non-ische	Inverse va	35	-0.0167	0.01546	0.2802	0.9834	0.9541	1.0137
DNA metl	Non-ische	Simple mo	35	-0.0382	0.04138	0.3628	0.9625	0.8876	1.0439
DNA metl	Non-ische	Weighted	35	-0.0272	0.03701	0.467	0.9731	0.9051	1.0464
DNA metl	Non-ische	Maximum	14	3.6E-05	3.1E-05	0.2339	1.0000	1.0000	1.0001
DNA metl	Non-ische	MR Egger	14	4.1E-05	3.8E-05	0.3022	1.0000	1.0000	1.0001
DNA metl	Non-ische	Weighted	14	-3E-06	4.5E-05	0.9438	1.0000	0.9999	1.0001
DNA metl	Non-ische	Inverse va	14	3.6E-05	3E-05	0.2365	1.0000	1.0000	1.0001
DNA metl	Non-ische	Simple mo	14	4.4E-05	8.7E-05	0.6184	1.0000	0.9999	1.0002

DNA metl	Non-ische	Weighted	14	4.4E-05	8.1E-05	0.5961	1.0000	0.9999	1.0002
DNA metl	Non-ische	Maximum	33	-0.0017	0.01163	0.881	0.9983	0.9758	1.0213
DNA metl	Non-ische	MR Egger	33	0.00779	0.03061	0.8007	1.0078	0.9491	1.0701
DNA metl	Non-ische	Weighted	33	-0.0002	0.01597	0.9887	0.9998	0.9690	1.0316
DNA metl	Non-ische	Inverse va	33	-0.0017	0.01309	0.8996	0.9984	0.9731	1.0243
DNA metl	Non-ische	Simple mo	33	0.01806	0.03155	0.571	1.0182	0.9572	1.0832
DNA metl	Non-ische	Weighted	33	0.0201	0.02841	0.4845	1.0203	0.9650	1.0787
DNA metl	Pulmonar	Maximum	16	0.1676	1.452	0.9081	1.1825	0.0687	20.3589
DNA metl	Pulmonar	MR Egger	16	-2.544	5.224	0.6338	0.0786	0.0000	#######
DNA metl	Pulmonar	Weighted	16	-1.172	2.181	0.591	0.3097	0.0043	22.2593
DNA metl	Pulmonar	Inverse va	16	0.1594	1.842	0.931	1.1728	0.0317	43.3679
DNA metl	Pulmonar	Simple mo	16	2.084	4.112	0.6196	8.0366	0.0025	#######
DNA metl	Pulmonar	Weighted	16	-3.787	4.05	0.3645	0.0227	0.0000	63.4975
DNA metl	Pulmonar	Maximum	24	0.00038	0.02698	0.9887	1.0004	0.9489	1.0547
DNA metl	Pulmonar	MR Egger	24	-0.0701	0.07084	0.333	0.9323	0.8114	1.0711
DNA metl	Pulmonar	Weighted	24	-0.001	0.03677	0.9778	0.9990	0.9295	1.0736
DNA metl	Pulmonar	Inverse va	24	0.00039	0.03087	0.9899	1.0004	0.9417	1.0628
DNA metl	Pulmonar	Simple mo	24	9.9E-05	0.07142	0.9989	1.0001	0.8695	1.1504
DNA metl	Pulmonar	Weighted	24	-0.0213	0.05618	0.7083	0.9789	0.8769	1.0929
DNA metl	Pulmonar	Maximum	35	-0.0313	0.0235	0.1826	0.9692	0.9255	1.0148
DNA metl	Pulmonar	MR Egger	35	-0.1213	0.07941	0.1361	0.8858	0.7581	1.0349
DNA metl	Pulmonar	Weighted	35	-0.0477	0.03701	0.197	0.9534	0.8867	1.0251
DNA metl	Pulmonar	Inverse va	35	-0.0302	0.0263	0.2511	0.9703	0.9215	1.0216
DNA metl	Pulmonar	Simple mo	35	-0.018	0.07417	0.8093	0.9821	0.8492	1.1358
DNA metl	Pulmonar	Weighted	35	-0.051	0.06196	0.416	0.9503	0.8416	1.0730
DNA metl	Pulmonar	Maximum	14	7.7E-05	4.5E-05	0.0888	1.0001	1.0000	1.0002
DNA metl	Pulmonar	MR Egger	14	6.2E-05	5.4E-05	0.2679	1.0001	1.0000	1.0002
DNA metl	Pulmonar	Weighted	14	8E-05	5.7E-05	0.1576	1.0001	1.0000	1.0002
DNA metl	Pulmonar	Inverse va	14	7.8E-05	4.5E-05	0.08364	1.0001	1.0000	1.0002
DNA metl	Pulmonar	Simple mo	14	8.8E-05	0.00014	0.5477	1.0001	0.9998	1.0004
DNA metl	Pulmonar	Weighted	14	8.8E-05	0.00014	0.5489	1.0001	0.9998	1.0004
DNA metl	Pulmonar	Maximum	33	-0.0353	0.01703	0.03832	0.9653	0.9337	0.9981
DNA metl	Pulmonar	MR Egger	33	-0.0528	0.03868	0.182	0.9486	0.8793	1.0233
DNA metl	Pulmonar	Weighted	33	-0.0307	0.02367	0.1952	0.9698	0.9258	1.0159
DNA metl	Pulmonar	Inverse va	33	-0.0355	0.01676	0.0343	0.9652	0.9340	0.9974
DNA metl	Pulmonar	Simple mo	33	0.00778	0.04464	0.8627	1.0078	0.9234	1.1000
DNA metl	Pulmonar	Weighted	33	-0.0553	0.03852	0.1608	0.9462	0.8774	1.0204
DNA metl	Valvular h	Maximum	16	0.2658	0.5804	0.647	1.3045	0.4182	4.0689
DNA metl	Valvular h	MR Egger	16	-0.9134	1.983	0.6521	0.4012	0.0082	19.5560
DNA metl	Valvular h	Weighted	16	0.6079	0.8121	0.4541	1.8366	0.3739	9.0215
DNA metl	Valvular h	Inverse va	16	0.2531	0.7019	0.7184	1.2880	0.3254	5.0979
DNA metl	Valvular h	Simple mo	16	-0.4018	1.49	0.791	0.6691	0.0361	12.4112

DNA metl	Valvular h	Weighted	16	0.3209	1.214	0.7952	1.3784	0.1276	14.8848
DNA metl	Valvular h	Maximum	24	0.00785	0.01063	0.4601	1.0079	0.9871	1.0291
DNA metl	Valvular h	MR Egger	24	-0.0084	0.02424	0.7326	0.9916	0.9456	1.0399
DNA metl	Valvular h	Weighted	24	0.01116	0.01557	0.4737	1.0112	0.9808	1.0426
DNA metl	Valvular h	Inverse va	24	0.00761	0.0105	0.4683	1.0076	0.9871	1.0286
DNA metl	Valvular h	Simple mo	24	0.03436	0.02737	0.2219	1.0350	0.9809	1.0920
DNA metl	Valvular h	Weighted	24	0.02082	0.02171	0.3476	1.0210	0.9785	1.0654
DNA metl	Valvular h	Maximum	35	0.00399	0.00939	0.6708	1.0040	0.9857	1.0226
DNA metl	Valvular h	MR Egger	35	0.00447	0.03232	0.8909	1.0045	0.9428	1.0702
DNA metl	Valvular h	Weighted	35	0.01734	0.01323	0.1901	1.0175	0.9914	1.0442
DNA metl	Valvular h	Inverse va	35	0.00383	0.01047	0.7145	1.0038	0.9834	1.0246
DNA metl	Valvular h	Simple mo	35	0.01611	0.02267	0.4823	1.0162	0.9721	1.0624
DNA metl	Valvular h	Weighted	35	0.0219	0.01875	0.2508	1.0221	0.9853	1.0604
DNA metl	Valvular h	Maximum	14	2.7E-05	1.8E-05	0.1366	1.0000	1.0000	1.0001
DNA metl	Valvular h	MR Egger	14	3E-05	3E-05	0.3371	1.0000	1.0000	1.0001
DNA metl	Valvular h	Weighted	14	3.1E-05	2.6E-05	0.2292	1.0000	1.0000	1.0001
DNA metl	Valvular h	Inverse va	14	2.6E-05	2.4E-05	0.2754	1.0000	1.0000	1.0001
DNA metl	Valvular h	Simple mo	14	1.6E-06	5.2E-05	0.9765	1.0000	0.9999	1.0001
DNA metl	Valvular h	Weighted	14	1.6E-06	4.6E-05	0.9734	1.0000	0.9999	1.0001
DNA metl	Valvular h	Maximum	33	0.00033	0.00686	0.962	1.0003	0.9870	1.0139
DNA metl	Valvular h	MR Egger	33	0.02535	0.01887	0.189	1.0257	0.9884	1.0643
DNA metl	Valvular h	Weighted	33	-0.0032	0.01077	0.7655	0.9968	0.9760	1.0181
DNA metl	Valvular h	Inverse va	33	0.00032	0.00833	0.9697	1.0003	0.9841	1.0168
DNA metl	Valvular h	Simple mo	33	-0.0073	0.02175	0.7404	0.9928	0.9513	1.0360
DNA metl	Valvular h	Weighted	33	-0.0016	0.0176	0.9298	0.9984	0.9646	1.0335

exposure	id.exposu	outcome	id.outcon	method	Q	Q_df	Q_pval
DNA met	ebi-a-GCS	Atrial fibr	ebi-a-GCS	MR Egger	28.1325	15	0.02075
DNA met	ebi-a-GCS	Atrial fibr	ebi-a-GCS	Inverse va	28.6543	16	0.02637
DNA met	ebi-a-GCS	Heart failu	ebi-a-GCS	MR Egger	14.8813	15	0.46
DNA met	ebi-a-GCS	Heart failu	ebi-a-GCS	Inverse va	14.9027	16	0.53178
DNA met	ebi-a-GCS	Cardiac ar	finn-b-CA	MR Egger	18.9308	14	0.16761
DNA met	ebi-a-GCS	Cardiac ar	finn-b-CA	Inverse va	22.4292	15	0.09703
DNA met	ebi-a-GCS	Cardiomy	finn-b-I9_	MR Egger	11.6788	14	0.63208
DNA met	ebi-a-GCS	Cardiomy	finn-b-I9_	Inverse va	13.6408	15	0.55293
DNA met	ebi-a-GCS	Hypertrop	finn-b-I9_	MR Egger	12.7674	14	0.54491
DNA met	ebi-a-GCS	Hypertrop	finn-b-I9_	Inverse va	15.7614	15	0.39808
DNA met	ebi-a-GCS	Hypertens	finn-b-I9_	MR Egger	33.724	14	0.00226
DNA met	ebi-a-GCS	Hypertens	finn-b-I9_	Inverse va	35.4102	15	0.00215
DNA met	ebi-a-GCS	Ischemic l	finn-b-I9_	MR Egger	32.6644	14	0.00322
DNA met	ebi-a-GCS	Ischemic l	finn-b-I9_	Inverse va	32.6656	15	0.00522
DNA met	ebi-a-GCS	Non-ische	finn-b-I9_	MR Egger	10.2481	14	0.74383
DNA met	ebi-a-GCS	Non-ische	finn-b-I9_	Inverse va	10.5603	15	0.7831
DNA met	ebi-a-GCS	Pulmonary	finn-b-I9_	MR Egger	17.5777	14	0.2267
DNA met	ebi-a-GCS	Pulmonary	finn-b-I9_	Inverse va	18.5542	15	0.23465
DNA met	ebi-a-GCS	Valvular h	finn-b-I9_	MR Egger	23.1926	14	0.05721
DNA met	ebi-a-GCS	Valvular h	finn-b-I9_	Inverse va	24.4603	15	0.05768
DNA met	ebi-a-GCS	Coronary	ieu-a-7	MR Egger	28.8259	14	0.01103
DNA met	ebi-a-GCS	Coronary	ieu-a-7	Inverse va	29.0668	15	0.01577
DNA met	ebi-a-GCS	Myocardia	ieu-a-798	MR Egger	23.5125	14	0.05243
DNA met	ebi-a-GCS	Myocardia	ieu-a-798	Inverse va	23.9845	15	0.06536
DNA met	ebi-a-GCS	Atrial fibr	ebi-a-GCS	MR Egger	17.7045	24	0.8171
DNA met	ebi-a-GCS	Atrial fibr	ebi-a-GCS	Inverse va	18.1115	25	0.83762
DNA met	ebi-a-GCS	Heart failu	ebi-a-GCS	MR Egger	17.3748	21	0.68815
DNA met	ebi-a-GCS	Heart failu	ebi-a-GCS	Inverse va	17.4666	22	0.73711
DNA met	ebi-a-GCS	Cardiac ar	finn-b-CA	MR Egger	19.5897	22	0.60869
DNA met	ebi-a-GCS	Cardiac ar	finn-b-CA	Inverse va	20.0914	23	0.63639
DNA met	ebi-a-GCS	Cardiomy	finn-b-I9_	MR Egger	18.7055	22	0.66344
DNA met	ebi-a-GCS	Cardiomy	finn-b-I9_	Inverse va	20.1503	23	0.63283
DNA met	ebi-a-GCS	Hypertrop	finn-b-I9_	MR Egger	24.2749	22	0.33299
DNA met	ebi-a-GCS	Hypertrop	finn-b-I9_	Inverse va	24.2881	23	0.388
DNA met	ebi-a-GCS	Hypertens	finn-b-I9_	MR Egger	50.4381	22	0.00051
DNA met	ebi-a-GCS	Hypertens	finn-b-I9_	Inverse va	50.6471	23	0.00076
DNA met	ebi-a-GCS	Ischemic l	finn-b-I9_	MR Egger	29.9667	22	0.11928
DNA met	ebi-a-GCS	Ischemic l	finn-b-I9_	Inverse va	30.3994	23	0.13828
DNA met	ebi-a-GCS	Non-ische	finn-b-I9_	MR Egger	14.1815	22	0.89491
DNA met	ebi-a-GCS	Non-ische	finn-b-I9_	Inverse va	16.6018	23	0.82832
DNA met	ebi-a-GCS	Pulmonary	finn-b-I9_	MR Egger	26.6324	22	0.22552

DNA met	ebi-a-GCS	Pulmonar	finn-b-I9_	Inverse va	28.3324	23	0.20347
DNA met	ebi-a-GCS	Valvular l	finn-b-I9_	MR Egger	16.8705	22	0.77047
DNA met	ebi-a-GCS	Valvular l	finn-b-I9_	Inverse va	17.4961	23	0.78422
DNA met	ebi-a-GCS	Coronary	ieu-a-7	MR Egger	28.0113	23	0.21536
DNA met	ebi-a-GCS	Coronary	ieu-a-7	Inverse va	28.0982	24	0.25592
DNA met	ebi-a-GCS	Myocardia	ieu-a-798	MR Egger	19.7086	23	0.65942
DNA met	ebi-a-GCS	Myocardia	ieu-a-798	Inverse va	20.094	24	0.69142
DNA met	ebi-a-GCS	Atrial fibr	ebi-a-GCS	MR Egger	81.8946	36	2E-05
DNA met	ebi-a-GCS	Atrial fibr	ebi-a-GCS	Inverse va	82.4272	37	2.6E-05
DNA met	ebi-a-GCS	Heart failu	ebi-a-GCS	MR Egger	30.656	32	0.53453
DNA met	ebi-a-GCS	Heart failu	ebi-a-GCS	Inverse va	30.9072	33	0.57167
DNA met	ebi-a-GCS	Cardiac ar	finn-b-CA	MR Egger	37.655	37	0.43913
DNA met	ebi-a-GCS	Cardiac ar	finn-b-CA	Inverse va	37.9044	38	0.47385
DNA met	ebi-a-GCS	Cardiomy	finn-b-I9_	MR Egger	63.995	37	0.00383
DNA met	ebi-a-GCS	Cardiomy	finn-b-I9_	Inverse va	64.0371	38	0.00517
DNA met	ebi-a-GCS	Hypertrop	finn-b-I9_	MR Egger	39.9955	37	0.33858
DNA met	ebi-a-GCS	Hypertrop	finn-b-I9_	Inverse va	40.3028	38	0.36874
DNA met	ebi-a-GCS	Hypertens	finn-b-I9_	MR Egger	62.8867	37	0.005
DNA met	ebi-a-GCS	Hypertens	finn-b-I9_	Inverse va	62.891	38	0.00675
DNA met	ebi-a-GCS	Ischemic l	finn-b-I9_	MR Egger	36.448	37	0.49474
DNA met	ebi-a-GCS	Ischemic l	finn-b-I9_	Inverse va	36.4785	38	0.53986
DNA met	ebi-a-GCS	Non-ische	finn-b-I9_	MR Egger	33.5288	37	0.63257
DNA met	ebi-a-GCS	Non-ische	finn-b-I9_	Inverse va	34.1712	38	0.64716
DNA met	ebi-a-GCS	Pulmonar	finn-b-I9_	MR Egger	43.944	37	0.20099
DNA met	ebi-a-GCS	Pulmonar	finn-b-I9_	Inverse va	45.8532	38	0.17861
DNA met	ebi-a-GCS	Valvular l	finn-b-I9_	MR Egger	47.3612	37	0.11833
DNA met	ebi-a-GCS	Valvular l	finn-b-I9_	Inverse va	47.6477	38	0.13569
DNA met	ebi-a-GCS	Coronary	ieu-a-7	MR Egger	42.8688	35	0.16943
DNA met	ebi-a-GCS	Coronary	ieu-a-7	Inverse va	45.16	36	0.14082
DNA met	ebi-a-GCS	Myocardia	ieu-a-798	MR Egger	51.5809	35	0.03504
DNA met	ebi-a-GCS	Myocardia	ieu-a-798	Inverse va	51.9633	36	0.0414
DNA met	ebi-a-GCS	Atrial fibr	ebi-a-GCS	MR Egger	14.9428	15	0.45555
DNA met	ebi-a-GCS	Atrial fibr	ebi-a-GCS	Inverse va	14.9441	16	0.52873
DNA met	ebi-a-GCS	Heart failu	ebi-a-GCS	MR Egger	12.8234	12	0.38201
DNA met	ebi-a-GCS	Heart failu	ebi-a-GCS	Inverse va	14.2191	13	0.3586
DNA met	ebi-a-GCS	Cardiac ar	finn-b-CA	MR Egger	21.8852	15	0.11085
DNA met	ebi-a-GCS	Cardiac ar	finn-b-CA	Inverse va	22.3335	16	0.13275
DNA met	ebi-a-GCS	Cardiomy	finn-b-I9_	MR Egger	20.6641	15	0.14791
DNA met	ebi-a-GCS	Cardiomy	finn-b-I9_	Inverse va	23.8451	16	0.09294
DNA met	ebi-a-GCS	Hypertrop	finn-b-I9_	MR Egger	9.56822	15	0.84599
DNA met	ebi-a-GCS	Hypertrop	finn-b-I9_	Inverse va	9.5993	16	0.8867
DNA met	ebi-a-GCS	Hypertens	finn-b-I9_	MR Egger	24.9288	15	0.05091

DNA met	ebi-a-GCS	Hypertens	finn-b-I9_	Inverse va	25.8256	16	0.05653
DNA met	ebi-a-GCS	Ischemic l	finn-b-I9_	MR Egger	13.625	15	0.55414
DNA met	ebi-a-GCS	Ischemic l	finn-b-I9_	Inverse va	15.6231	16	0.47956
DNA met	ebi-a-GCS	Non-ische	finn-b-I9_	MR Egger	13.4516	15	0.56746
DNA met	ebi-a-GCS	Non-ische	finn-b-I9_	Inverse va	13.6379	16	0.62567
DNA met	ebi-a-GCS	Pulmonar	finn-b-I9_	MR Egger	10.3925	15	0.79435
DNA met	ebi-a-GCS	Pulmonar	finn-b-I9_	Inverse va	10.3965	16	0.84512
DNA met	ebi-a-GCS	Valvular l	finn-b-I9_	MR Egger	23.945	15	0.06603
DNA met	ebi-a-GCS	Valvular l	finn-b-I9_	Inverse va	23.9839	16	0.08986
DNA met	ebi-a-GCS	Coronary	ieu-a-7	MR Egger	18.2421	15	0.25015
DNA met	ebi-a-GCS	Coronary	ieu-a-7	Inverse va	20.2406	16	0.20958
DNA met	ebi-a-GCS	Myocardia	ieu-a-798	MR Egger	16.192	14	0.30179
DNA met	ebi-a-GCS	Myocardia	ieu-a-798	Inverse va	17.2623	15	0.30343
DNA met	ebi-a-GCS	Atrial fibr	ebi-a-GCS	MR Egger	56.6568	32	0.00461
DNA met	ebi-a-GCS	Atrial fibr	ebi-a-GCS	Inverse va	57.7534	33	0.00487
DNA met	ebi-a-GCS	Heart failu	ebi-a-GCS	MR Egger	35.2873	30	0.23234
DNA met	ebi-a-GCS	Heart failu	ebi-a-GCS	Inverse va	35.3335	31	0.27077
DNA met	ebi-a-GCS	Cardiac ar	finn-b-CA	MR Egger	46.6245	31	0.03548
DNA met	ebi-a-GCS	Cardiac ar	finn-b-CA	Inverse va	48.3085	32	0.03222
DNA met	ebi-a-GCS	Cardiomy	finn-b-I9_	MR Egger	35.1451	31	0.27809
DNA met	ebi-a-GCS	Cardiomy	finn-b-I9_	Inverse va	36.496	32	0.26757
DNA met	ebi-a-GCS	Hypertrop	finn-b-I9_	MR Egger	39.676	31	0.13651
DNA met	ebi-a-GCS	Hypertrop	finn-b-I9_	Inverse va	40.1565	32	0.15251
DNA met	ebi-a-GCS	Hypertens	finn-b-I9_	MR Egger	59.2139	31	0.00167
DNA met	ebi-a-GCS	Hypertens	finn-b-I9_	Inverse va	59.3055	32	0.00234
DNA met	ebi-a-GCS	Ischemic l	finn-b-I9_	MR Egger	15.5498	31	0.99056
DNA met	ebi-a-GCS	Ischemic l	finn-b-I9_	Inverse va	17.0833	32	0.98559
DNA met	ebi-a-GCS	Non-ische	finn-b-I9_	MR Egger	45.0361	31	0.04948
DNA met	ebi-a-GCS	Non-ische	finn-b-I9_	Inverse va	45.0371	32	0.06295
DNA met	ebi-a-GCS	Pulmonar	finn-b-I9_	MR Egger	25.1903	31	0.75914
DNA met	ebi-a-GCS	Pulmonar	finn-b-I9_	Inverse va	25.7749	32	0.77348
DNA met	ebi-a-GCS	Valvular l	finn-b-I9_	MR Egger	43.2222	31	0.07112
DNA met	ebi-a-GCS	Valvular l	finn-b-I9_	Inverse va	46.2451	32	0.04949
DNA met	ebi-a-GCS	Coronary	ieu-a-7	MR Egger	40.7295	33	0.16687
DNA met	ebi-a-GCS	Coronary	ieu-a-7	Inverse va	41.0293	34	0.18955
DNA met	ebi-a-GCS	Myocardia	ieu-a-798	MR Egger	38.682	32	0.19341
DNA met	ebi-a-GCS	Myocardia	ieu-a-798	Inverse va	39.3012	33	0.20838

exposure	id.exposu	outcome	id.outcon	egger_int	se	pval
DNA met	ebi-a-GC!	Atrial fibr	ebi-a-GC!	-0.00457	0.00867	0.60559
DNA met	ebi-a-GC!	Heart fail	ebi-a-GC!	-0.00111	0.00758	0.88576
DNA met	ebi-a-GC!	Cardiac ar	finn-b-CA	0.01842	0.01145	0.13004
DNA met	ebi-a-GC!	Cardiomy	finn-b-I9_	0.0348	0.02484	0.18307
DNA met	ebi-a-GC!	Hypertrop	finn-b-I9_	0.09594	0.05545	0.10555
DNA met	ebi-a-GC!	Hypertens	finn-b-I9_	0.01089	0.01301	0.41683
DNA met	ebi-a-GC!	Ischemic l	finn-b-I9_	0.00035	0.01551	0.98217
DNA met	ebi-a-GC!	Non-ische	finn-b-I9_	-0.00752	0.01347	0.58518
DNA met	ebi-a-GC!	Pulmonar	finn-b-I9_	0.01982	0.02247	0.39272
DNA met	ebi-a-GC!	Valvular l	finn-b-I9_	0.00902	0.01031	0.39645
DNA met	ebi-a-GC!	Coronary	ieu-a-7	-0.00501	0.01465	0.73739
DNA met	ebi-a-GC!	Myocardi	ieu-a-798	-0.00773	0.01458	0.60433
DNA met	ebi-a-GC!	Atrial fibr	ebi-a-GC!	0.00326	0.00512	0.52955
DNA met	ebi-a-GC!	Heart fail	ebi-a-GC!	-0.00212	0.00701	0.76494
DNA met	ebi-a-GC!	Cardiac ar	finn-b-CA	0.00464	0.00655	0.48617
DNA met	ebi-a-GC!	Cardiomy	finn-b-I9_	0.01983	0.0165	0.24214
DNA met	ebi-a-GC!	Hypertrop	finn-b-I9_	0.00419	0.03834	0.91395
DNA met	ebi-a-GC!	Hypertens	finn-b-I9_	0.00255	0.00844	0.76557
DNA met	ebi-a-GC!	Ischemic l	finn-b-I9_	-0.00444	0.00788	0.5787
DNA met	ebi-a-GC!	Non-ische	finn-b-I9_	0.01391	0.00894	0.13404
DNA met	ebi-a-GC!	Pulmonar	finn-b-I9_	0.01738	0.01466	0.24864
DNA met	ebi-a-GC!	Valvular l	finn-b-I9_	0.00421	0.00533	0.4374
DNA met	ebi-a-GC!	Coronary	ieu-a-7	-0.0023	0.00861	0.79177
DNA met	ebi-a-GC!	Myocardi	ieu-a-798	-0.00538	0.00866	0.54081
DNA met	ebi-a-GC!	Atrial fibr	ebi-a-GC!	0.00294	0.00607	0.63142
DNA met	ebi-a-GC!	Heart fail	ebi-a-GC!	-0.00272	0.00543	0.61963
DNA met	ebi-a-GC!	Cardiac ar	finn-b-CA	-0.00353	0.00714	0.62351
DNA met	ebi-a-GC!	Cardiomy	finn-b-I9_	-0.00366	0.02347	0.87689
DNA met	ebi-a-GC!	Hypertrop	finn-b-I9_	-0.02207	0.04138	0.59705
DNA met	ebi-a-GC!	Hypertens	finn-b-I9_	-0.00039	0.00785	0.96016
DNA met	ebi-a-GC!	Ischemic l	finn-b-I9_	0.00128	0.0073	0.86226
DNA met	ebi-a-GC!	Non-ische	finn-b-I9_	-0.00775	0.00967	0.42798
DNA met	ebi-a-GC!	Pulmonar	finn-b-I9_	0.0199	0.01569	0.21276
DNA met	ebi-a-GC!	Valvular l	finn-b-I9_	-0.00308	0.00651	0.6389
DNA met	ebi-a-GC!	Coronary	ieu-a-7	-0.0085	0.00621	0.18012
DNA met	ebi-a-GC!	Myocardi	ieu-a-798	-0.00387	0.00761	0.61366
DNA met	ebi-a-GC!	Atrial fibr	ebi-a-GC!	0.00015	0.00395	0.9709
DNA met	ebi-a-GC!	Heart fail	ebi-a-GC!	0.00661	0.00578	0.2754
DNA met	ebi-a-GC!	Cardiac ar	finn-b-CA	0.00432	0.00779	0.58754
DNA met	ebi-a-GC!	Cardiomy	finn-b-I9_	-0.02902	0.0191	0.14941
DNA met	ebi-a-GC!	Hypertrop	finn-b-I9_	-0.0064	0.03628	0.86243

DNA met	ebi-a-GCS	Hypertens	finn-b-I9_	0.00522	0.0071	0.4739
DNA met	ebi-a-GCS	Ischemic l	finn-b-I9_	0.0094	0.00665	0.17791
DNA met	ebi-a-GCS	Non-ische	finn-b-I9_	-0.00381	0.00883	0.67208
DNA met	ebi-a-GCS	Pulmonar	finn-b-I9_	-0.00083	0.01314	0.95067
DNA met	ebi-a-GCS	Valvular l	finn-b-I9_	0.00104	0.00663	0.87809
DNA met	ebi-a-GCS	Coronary	ieu-a-7	-0.00767	0.00598	0.21933
DNA met	ebi-a-GCS	Myocardi	ieu-a-798	-0.00625	0.0065	0.35239
DNA met	ebi-a-GCS	Atrial fibr	ebi-a-GCS	0.00333	0.00423	0.43706
DNA met	ebi-a-GCS	Heart fail	ebi-a-GCS	0.00093	0.00467	0.84421
DNA met	ebi-a-GCS	Cardiac ar	finn-b-CA	0.00679	0.00642	0.29816
DNA met	ebi-a-GCS	Cardiomy	finn-b-I9_	-0.01534	0.01405	0.28342
DNA met	ebi-a-GCS	Hypertrop	finn-b-I9_	-0.02039	0.03328	0.54455
DNA met	ebi-a-GCS	Hypertens	finn-b-I9_	0.00135	0.00617	0.82809
DNA met	ebi-a-GCS	Ischemic l	finn-b-I9_	0.0067	0.00541	0.22489
DNA met	ebi-a-GCS	Non-ische	finn-b-I9_	-0.00023	0.00863	0.97911
DNA met	ebi-a-GCS	Pulmonar	finn-b-I9_	0.00816	0.01067	0.45031
DNA met	ebi-a-GCS	Valvular l	finn-b-I9_	-0.00741	0.00503	0.15099
DNA met	ebi-a-GCS	Coronary	ieu-a-7	0.00247	0.005	0.62538
DNA met	ebi-a-GCS	Myocardi	ieu-a-798	0.00399	0.00558	0.47936

SNP	CHR	POS	Exposure	samplesiz	Outcome	samplesiz	effect alle	other alle	Beta
rs115353(	5	7.2E+07	DNA metl	34470	Cardiac ar	172155	T	C	0.0064
rs121888(	5	4.5E+07	DNA metl	34470	Cardiac ar	172155	C	A	-0.0101
rs131336	4	3.8E+07	DNA metl	34470	Cardiac ar	172155	C	T	-0.004
rs132400	13	4.3E+07	DNA metl	34470	Cardiac ar	172155	T	C	0.004
rs147021	3	6.7E+07	DNA metl	34470	Cardiac ar	172155	T	C	-0.0102
rs218923	4	1.1E+08	DNA metl	34470	Cardiac ar	172155	G	T	-0.0037
rs358360(	2	1.1E+08	DNA metl	34470	Cardiac ar	172155	G	A	0.0045
rs549280	4	7.5E+07	DNA metl	34470	Cardiac ar	172155	A	G	-0.0041
rs559982	11	7.4E+07	DNA metl	34470	Cardiac ar	172155	G	A	0.0078
rs606977	20	5.5E+07	DNA metl	34470	Cardiac ar	172155	T	C	0.0035
rs609287	9	9.1E+07	DNA metl	34470	Cardiac ar	172155	C	T	0.0049
rs697937	7	2.9E+07	DNA metl	34470	Cardiac ar	172155	A	G	0.0036
rs726831	8	1.1E+08	DNA metl	34470	Cardiac ar	172155	G	A	-0.0056
rs777009	6	4.1E+07	DNA metl	34470	Cardiac ar	172155	A	G	-0.017
rs785728	2	4.4E+07	DNA metl	34470	Cardiac ar	172155	C	T	0.0071
rs807045	17	3.8E+07	DNA metl	34470	Cardiac ar	172155	T	C	0.0061
rs108494	12	6493351	DNA metl	34463	Cardiac ar	172155	G	A	-0.2346
rs111901	10	1E+08	DNA metl	34463	Cardiac ar	172155	A	C	0.2484
rs112106	3	3.8E+07	DNA metl	34463	Cardiac ar	172155	A	G	1.483
rs112452(	2	1.3E+08	DNA metl	34463	Cardiac ar	172155	A	G	0.7517
rs112533	10	759559	DNA metl	34463	Cardiac ar	172155	T	C	0.2846
rs114234	6	1.8E+07	DNA metl	34463	Cardiac ar	172155	C	T	-0.8235
rs116853	17	5.5E+07	DNA metl	34463	Cardiac ar	172155	A	G	0.5517
rs128188	12	8.2E+07	DNA metl	34463	Cardiac ar	172155	T	C	0.2944
rs148794	1	8895237	DNA metl	34463	Cardiac ar	172155	A	G	-0.2109
rs156629	17	7.1E+07	DNA metl	34463	Cardiac ar	172155	C	T	-0.179
rs167045	11	7.7E+07	DNA metl	34463	Cardiac ar	172155	G	A	0.2007
rs199005	7	4.5E+07	DNA metl	34463	Cardiac ar	172155	A	G	0.2573
rs230098	10	1E+08	DNA metl	34463	Cardiac ar	172155	G	A	0.1833
rs264726(	4	1.1E+08	DNA metl	34463	Cardiac ar	172155	G	A	0.176
rs375938	17	1.5E+07	DNA metl	34463	Cardiac ar	172155	A	G	-0.1904
rs382995	17	3378876	DNA metl	34463	Cardiac ar	172155	T	C	-0.3796
rs467051	2	3.6E+07	DNA metl	34463	Cardiac ar	172155	A	C	0.1927
rs533852	3	1E+08	DNA metl	34463	Cardiac ar	172155	T	C	-0.1741
rs575118(	22	2.3E+07	DNA metl	34463	Cardiac ar	172155	T	C	-0.3915
rs644067(	3	1.5E+08	DNA metl	34463	Cardiac ar	172155	A	G	-0.2251
rs653111	2	1.7E+07	DNA metl	34463	Cardiac ar	172155	C	T	0.2542
rs678553	1	2.4E+08	DNA metl	34463	Cardiac ar	172155	C	T	-0.3265
rs716433	15	7.8E+07	DNA metl	34463	Cardiac ar	172155	C	T	-0.2031
rs727655(	2	1828908	DNA metl	34463	Cardiac ar	172155	T	C	-0.9593
rs730280	11	1.2E+08	DNA metl	34463	Cardiac ar	172155	A	G	-0.4329

rs752223	1	6E+07	DNA meth	34463	Cardiac ar	172155	A	G	-0.5602
rs7677639	4	1.9E+08	DNA meth	34463	Cardiac ar	172155	C	T	-0.2755
rs7913917	10	3.1E+07	DNA meth	34463	Cardiac ar	172155	T	C	0.1874
rs7982088	1	1.6E+08	DNA meth	34463	Cardiac ar	172155	C	T	0.9005
rs927121	20	5.1E+07	DNA meth	34463	Cardiac ar	172155	C	T	-0.2149
rs9386256	6	1.5E+08	DNA meth	34463	Cardiac ar	172155	T	G	-0.372
rs9409787	9	9.7E+07	DNA meth	34463	Cardiac ar	172155	A	G	0.2691
rs9870681	3	7.2E+07	DNA meth	34463	Cardiac ar	172155	T	C	0.2221
rs1005277	10	3.8E+07	DNA meth	34449	Cardiac ar	172155	C	A	-0.3006
rs1078628	10	9.8E+07	DNA meth	34449	Cardiac ar	172155	G	A	0.36
rs1119013	10	1E+08	DNA meth	34449	Cardiac ar	172155	T	C	0.147
rs1146061	3	3.2E+07	DNA meth	34449	Cardiac ar	172155	T	C	0.3554
rs1151617	12	1.1E+07	DNA meth	34449	Cardiac ar	172155	A	G	0.1773
rs1200332	14	3.5E+07	DNA meth	34449	Cardiac ar	172155	C	T	-0.179
rs1219681	6	1.5E+08	DNA meth	34449	Cardiac ar	172155	T	C	0.4614
rs1227180	11	7.8E+07	DNA meth	34449	Cardiac ar	172155	A	G	0.364
rs1241775	11	6.6E+07	DNA meth	34449	Cardiac ar	172155	C	T	0.2093
rs1484362	2	1.1E+08	DNA meth	34449	Cardiac ar	172155	T	C	0.49
rs1598856	4	1E+08	DNA meth	34449	Cardiac ar	172155	G	A	-0.1858
rs1695845	17	9445045	DNA meth	34449	Cardiac ar	172155	C	T	0.1533
rs1849209	18	4.2E+07	DNA meth	34449	Cardiac ar	172155	T	G	-0.1647
rs1984968	14	9.6E+07	DNA meth	34449	Cardiac ar	172155	G	A	-0.2047
rs2014202	1	2.1E+08	DNA meth	34449	Cardiac ar	172155	A	G	0.1696
rs2195114	2	1.5E+08	DNA meth	34449	Cardiac ar	172155	C	T	0.1614
rs2647254	4	1.1E+08	DNA meth	34449	Cardiac ar	172155	A	G	-0.1436
rs290805	7	1.3E+08	DNA meth	34449	Cardiac ar	172155	T	C	-0.2268
rs3093956	6	3.1E+07	DNA meth	34449	Cardiac ar	172155	C	T	0.2428
rs4141989	11	1E+08	DNA meth	34449	Cardiac ar	172155	C	T	-0.8116
rs4679900	3	1.6E+08	DNA meth	34449	Cardiac ar	172155	T	C	0.1538
rs4770473	13	2.4E+07	DNA meth	34449	Cardiac ar	172155	T	C	0.159
rs4838595	10	5E+07	DNA meth	34449	Cardiac ar	172155	T	C	-0.2576
rs6054829	20	7203816	DNA meth	34449	Cardiac ar	172155	G	A	0.276
rs6238555	6	1.4E+07	DNA meth	34449	Cardiac ar	172155	T	C	0.279
rs6440670	3	1.5E+08	DNA meth	34449	Cardiac ar	172155	A	G	-0.1915
rs7017774	8	1340507	DNA meth	34449	Cardiac ar	172155	T	C	0.146
rs7705526	5	1285974	DNA meth	34449	Cardiac ar	172155	A	C	0.1811
rs7729949	7	4.9E+07	DNA meth	34449	Cardiac ar	172155	C	T	0.7584
rs7871226	12	5.5E+07	DNA meth	34449	Cardiac ar	172155	A	C	-0.231
rs7965945	12	2.5E+07	DNA meth	34449	Cardiac ar	172155	C	T	0.2095
rs8005306	14	2.1E+07	DNA meth	34449	Cardiac ar	172155	A	C	0.322
rs8019582	10	1.3E+08	DNA meth	34449	Cardiac ar	172155	C	T	-0.2695
rs878175	6	1.4E+08	DNA meth	34449	Cardiac ar	172155	C	T	0.1903

rs9813840	3	1.9E+08	DNA meth	34449	Cardiac ar	172155	C	T	-0.4373
rs1160862	12	5.3E+07	DNA meth	34448	Cardiac ar	172155	T	C	-590.41
rs1436732	8	1E+08	DNA meth	34448	Cardiac ar	172155	G	A	-368.48
rs1486942	16	9E+07	DNA meth	34448	Cardiac ar	172155	T	C	339.43
rs2046729	12	9.6E+07	DNA meth	34448	Cardiac ar	172155	T	C	0.3916
rs2498857	9	1.1E+08	DNA meth	34448	Cardiac ar	172155	C	T	0.3822
rs2851474	7	2288916	DNA meth	34448	Cardiac ar	172155	C	T	1604.03
rs5617386	22	5.1E+07	DNA meth	34448	Cardiac ar	172155	A	G	421.813
rs6206710	16	9E+07	DNA meth	34448	Cardiac ar	172155	T	C	443.729
rs7278637	21	4.3E+07	DNA meth	34448	Cardiac ar	172155	G	T	-0.9444
rs7492167	1	1.9E+07	DNA meth	34448	Cardiac ar	172155	A	G	634.602
rs7578093	4	1.9E+08	DNA meth	34448	Cardiac ar	172155	A	G	-355.66
rs7614650	7	1.4E+08	DNA meth	34448	Cardiac ar	172155	A	G	-439.48
rs7821260	22	5.1E+07	DNA meth	34448	Cardiac ar	172155	T	C	347.26
rs7888438	4	1.4E+08	DNA meth	34448	Cardiac ar	172155	T	C	-373.26
rs1152271	5	1.1E+08	DNA meth	34467	Cardiac ar	172155	T	C	-0.5517
rs1211671	1	1E+08	DNA meth	34467	Cardiac ar	172155	T	C	-0.266
rs1341264	2	1.1E+08	DNA meth	34467	Cardiac ar	172155	G	A	-0.3915
rs1389887	5	1.6E+08	DNA meth	34467	Cardiac ar	172155	A	G	0.59
rs1485456	6	1.5E+08	DNA meth	34467	Cardiac ar	172155	T	C	1.0581
rs1693623	10	8E+07	DNA meth	34467	Cardiac ar	172155	C	T	-0.1908
rs1709414	10	1E+08	DNA meth	34467	Cardiac ar	172155	G	A	0.18
rs1727988	15	8E+07	DNA meth	34467	Cardiac ar	172155	A	G	-0.1737
rs1800440	2	3.8E+07	DNA meth	34467	Cardiac ar	172155	C	T	0.1834
rs1837188	2	2E+08	DNA meth	34467	Cardiac ar	172155	C	T	0.1641
rs2010054	3	1.3E+08	DNA meth	34467	Cardiac ar	172155	G	A	0.1456
rs3817367	3	1.6E+08	DNA meth	34467	Cardiac ar	172155	G	T	-0.2347
rs4065321	17	3.8E+07	DNA meth	34467	Cardiac ar	172155	T	C	-0.1703
rs6709296	2	1.6E+08	DNA meth	34467	Cardiac ar	172155	T	G	0.151
rs6886532	5	1.5E+08	DNA meth	34467	Cardiac ar	172155	T	C	-0.3752
rs7265977	13	1E+08	DNA meth	34467	Cardiac ar	172155	T	C	0.2962
rs7618337	11	1.2E+07	DNA meth	34467	Cardiac ar	172155	T	C	0.2802
rs773853	19	1.7E+07	DNA meth	34467	Cardiac ar	172155	G	A	-0.174
rs7748865	4	1.9E+08	DNA meth	34467	Cardiac ar	172155	C	T	0.2283
rs790051	2	2.3E+08	DNA meth	34467	Cardiac ar	172155	A	G	-0.1676
rs7919238	10	1.8E+07	DNA meth	34467	Cardiac ar	172155	C	T	0.1787
rs887466	6	3.1E+07	DNA meth	34467	Cardiac ar	172155	A	G	-0.1928
rs9386796	6	1.1E+08	DNA meth	34467	Cardiac ar	172155	T	C	0.1983
rs9790675	4	1.1E+08	DNA meth	34467	Cardiac ar	172155	G	A	-0.2246
rs1005277	10	3.8E+07	DNA meth	34449	Cardiomy	159811	C	A	-0.3006
rs1078628	10	9.8E+07	DNA meth	34449	Cardiomy	159811	G	A	0.36
rs1119013	10	1E+08	DNA meth	34449	Cardiomy	159811	T	C	0.147

rs1146061	3	3.2E+07	DNA meth	34449	Cardiomy	159811	T	C	0.3554
rs1151617	12	1.1E+07	DNA meth	34449	Cardiomy	159811	A	G	0.1773
rs1200332	14	3.5E+07	DNA meth	34449	Cardiomy	159811	C	T	-0.179
rs1219681	6	1.5E+08	DNA meth	34449	Cardiomy	159811	T	C	0.4614
rs1227180	11	7.8E+07	DNA meth	34449	Cardiomy	159811	A	G	0.364
rs1241775	11	6.6E+07	DNA meth	34449	Cardiomy	159811	C	T	0.2093
rs1484362	2	1.1E+08	DNA meth	34449	Cardiomy	159811	T	C	0.49
rs1598856	4	1E+08	DNA meth	34449	Cardiomy	159811	G	A	-0.1858
rs1695845	17	9445045	DNA meth	34449	Cardiomy	159811	C	T	0.1533
rs1849209	18	4.2E+07	DNA meth	34449	Cardiomy	159811	T	G	-0.1647
rs1984968	14	9.6E+07	DNA meth	34449	Cardiomy	159811	G	A	-0.2047
rs2014202	1	2.1E+08	DNA meth	34449	Cardiomy	159811	A	G	0.1696
rs2195114	2	1.5E+08	DNA meth	34449	Cardiomy	159811	C	T	0.1614
rs2647254	4	1.1E+08	DNA meth	34449	Cardiomy	159811	A	G	-0.1436
rs290805	7	1.3E+08	DNA meth	34449	Cardiomy	159811	T	C	-0.2268
rs3093956	6	3.1E+07	DNA meth	34449	Cardiomy	159811	C	T	0.2428
rs4141989	11	1E+08	DNA meth	34449	Cardiomy	159811	C	T	-0.8116
rs4679900	3	1.6E+08	DNA meth	34449	Cardiomy	159811	T	C	0.1538
rs4770473	13	2.4E+07	DNA meth	34449	Cardiomy	159811	T	C	0.159
rs4838595	10	5E+07	DNA meth	34449	Cardiomy	159811	T	C	-0.2576
rs6054829	20	7203816	DNA meth	34449	Cardiomy	159811	G	A	0.276
rs6238555	6	1.4E+07	DNA meth	34449	Cardiomy	159811	T	C	0.279
rs6440670	3	1.5E+08	DNA meth	34449	Cardiomy	159811	A	G	-0.1915
rs7017774	8	1340507	DNA meth	34449	Cardiomy	159811	T	C	0.146
rs7705526	5	1285974	DNA meth	34449	Cardiomy	159811	A	C	0.1811
rs7729949	7	4.9E+07	DNA meth	34449	Cardiomy	159811	C	T	0.7584
rs7871226	12	5.5E+07	DNA meth	34449	Cardiomy	159811	A	C	-0.231
rs7965945	12	2.5E+07	DNA meth	34449	Cardiomy	159811	C	T	0.2095
rs8005306	14	2.1E+07	DNA meth	34449	Cardiomy	159811	A	C	0.322
rs8019582	10	1.3E+08	DNA meth	34449	Cardiomy	159811	C	T	-0.2695
rs878175	6	1.4E+08	DNA meth	34449	Cardiomy	159811	C	T	0.1903
rs9813840	3	1.9E+08	DNA meth	34449	Cardiomy	159811	C	T	-0.4373
rs1160862	12	5.3E+07	DNA meth	34448	Cardiomy	159811	T	C	-590.41
rs1436732	8	1E+08	DNA meth	34448	Cardiomy	159811	G	A	-368.48
rs1486942	16	9E+07	DNA meth	34448	Cardiomy	159811	T	C	339.43
rs2046729	12	9.6E+07	DNA meth	34448	Cardiomy	159811	T	C	0.3916
rs2498857	9	1.1E+08	DNA meth	34448	Cardiomy	159811	C	T	0.3822
rs2851474	7	2288916	DNA meth	34448	Cardiomy	159811	C	T	1604.03
rs5617386	22	5.1E+07	DNA meth	34448	Cardiomy	159811	A	G	421.813
rs6206710	16	9E+07	DNA meth	34448	Cardiomy	159811	T	C	443.729
rs7278637	21	4.3E+07	DNA meth	34448	Cardiomy	159811	G	T	-0.9444
rs7492167	1	1.9E+07	DNA meth	34448	Cardiomy	159811	A	G	634.602

rs7578093	4	1.9E+08	DNA meth	34448	Cardiomy	159811	A	G	-355.66
rs7614650	7	1.4E+08	DNA meth	34448	Cardiomy	159811	A	G	-439.48
rs7821260	22	5.1E+07	DNA meth	34448	Cardiomy	159811	T	C	347.26
rs7888438	4	1.4E+08	DNA meth	34448	Cardiomy	159811	T	C	-373.26
rs1084944	12	6493351	DNA meth	34463	Cardiomy	159811	G	A	-0.2346
rs1119012	10	1E+08	DNA meth	34463	Cardiomy	159811	A	C	0.2484
rs1121065	3	3.8E+07	DNA meth	34463	Cardiomy	159811	A	G	1.483
rs1124526	2	1.3E+08	DNA meth	34463	Cardiomy	159811	A	G	0.7517
rs1125333	10	759559	DNA meth	34463	Cardiomy	159811	T	C	0.2846
rs1142345	6	1.8E+07	DNA meth	34463	Cardiomy	159811	C	T	-0.8235
rs1168537	17	5.5E+07	DNA meth	34463	Cardiomy	159811	A	G	0.5517
rs1281881	12	8.2E+07	DNA meth	34463	Cardiomy	159811	T	C	0.2944
rs1487949	1	8895237	DNA meth	34463	Cardiomy	159811	A	G	-0.2109
rs1566295	17	7.1E+07	DNA meth	34463	Cardiomy	159811	C	T	-0.179
rs1670455	11	7.7E+07	DNA meth	34463	Cardiomy	159811	G	A	0.2007
rs1990053	7	4.5E+07	DNA meth	34463	Cardiomy	159811	A	G	0.2573
rs2300984	10	1E+08	DNA meth	34463	Cardiomy	159811	G	A	0.1833
rs2647266	4	1.1E+08	DNA meth	34463	Cardiomy	159811	G	A	0.176
rs375938	17	1.5E+07	DNA meth	34463	Cardiomy	159811	A	G	-0.1904
rs3829957	17	3378876	DNA meth	34463	Cardiomy	159811	T	C	-0.3796
rs4670518	2	3.6E+07	DNA meth	34463	Cardiomy	159811	A	C	0.1927
rs533852	3	1E+08	DNA meth	34463	Cardiomy	159811	T	C	-0.1741
rs5751180	22	2.3E+07	DNA meth	34463	Cardiomy	159811	T	C	-0.3915
rs6440670	3	1.5E+08	DNA meth	34463	Cardiomy	159811	A	G	-0.2251
rs6531114	2	1.7E+07	DNA meth	34463	Cardiomy	159811	C	T	0.2542
rs678553	1	2.4E+08	DNA meth	34463	Cardiomy	159811	C	T	-0.3265
rs7164338	15	7.8E+07	DNA meth	34463	Cardiomy	159811	C	T	-0.2031
rs7276556	2	1828908	DNA meth	34463	Cardiomy	159811	T	C	-0.9593
rs7302807	11	1.2E+08	DNA meth	34463	Cardiomy	159811	A	G	-0.4329
rs752223	1	6E+07	DNA meth	34463	Cardiomy	159811	A	G	-0.5602
rs7677639	4	1.9E+08	DNA meth	34463	Cardiomy	159811	C	T	-0.2755
rs7913917	10	3.1E+07	DNA meth	34463	Cardiomy	159811	T	C	0.1874
rs7982088	1	1.6E+08	DNA meth	34463	Cardiomy	159811	C	T	0.9005
rs927121	20	5.1E+07	DNA meth	34463	Cardiomy	159811	C	T	-0.2149
rs9386256	6	1.5E+08	DNA meth	34463	Cardiomy	159811	T	G	-0.372
rs9409787	9	9.7E+07	DNA meth	34463	Cardiomy	159811	A	G	0.2691
rs9870681	3	7.2E+07	DNA meth	34463	Cardiomy	159811	T	C	0.2221
rs1152271	5	1.1E+08	DNA meth	34467	Cardiomy	159811	T	C	-0.5517
rs1211671	1	1E+08	DNA meth	34467	Cardiomy	159811	T	C	-0.266
rs1341264	2	1.1E+08	DNA meth	34467	Cardiomy	159811	G	A	-0.3915
rs1389887	5	1.6E+08	DNA meth	34467	Cardiomy	159811	A	G	0.59
rs1485456	6	1.5E+08	DNA meth	34467	Cardiomy	159811	T	C	1.0581

rs1693623	10	8E+07	DNA meth	34467	Cardiomy	159811	C	T	-0.1908
rs1709414	10	1E+08	DNA meth	34467	Cardiomy	159811	G	A	0.18
rs1727988	15	8E+07	DNA meth	34467	Cardiomy	159811	A	G	-0.1737
rs1800440	2	3.8E+07	DNA meth	34467	Cardiomy	159811	C	T	0.1834
rs1837188	2	2E+08	DNA meth	34467	Cardiomy	159811	C	T	0.1641
rs2010054	3	1.3E+08	DNA meth	34467	Cardiomy	159811	G	A	0.1456
rs3817367	3	1.6E+08	DNA meth	34467	Cardiomy	159811	G	T	-0.2347
rs4065321	17	3.8E+07	DNA meth	34467	Cardiomy	159811	T	C	-0.1703
rs6709296	2	1.6E+08	DNA meth	34467	Cardiomy	159811	T	G	0.151
rs6886532	5	1.5E+08	DNA meth	34467	Cardiomy	159811	T	C	-0.3752
rs7265971	13	1E+08	DNA meth	34467	Cardiomy	159811	T	C	0.2962
rs7618331	11	1.2E+07	DNA meth	34467	Cardiomy	159811	T	C	0.2802
rs773853	19	1.7E+07	DNA meth	34467	Cardiomy	159811	G	A	-0.174
rs7748865	4	1.9E+08	DNA meth	34467	Cardiomy	159811	C	T	0.2283
rs790051	2	2.3E+08	DNA meth	34467	Cardiomy	159811	A	G	-0.1676
rs7919238	10	1.8E+07	DNA meth	34467	Cardiomy	159811	C	T	0.1787
rs887466	6	3.1E+07	DNA meth	34467	Cardiomy	159811	A	G	-0.1928
rs9386796	6	1.1E+08	DNA meth	34467	Cardiomy	159811	T	C	0.1983
rs9790675	4	1.1E+08	DNA meth	34467	Cardiomy	159811	G	A	-0.2246
rs1153530	5	7.2E+07	DNA meth	34470	Cardiomy	159811	T	C	0.0064
rs1218886	5	4.5E+07	DNA meth	34470	Cardiomy	159811	C	A	-0.0101
rs1313364	4	3.8E+07	DNA meth	34470	Cardiomy	159811	C	T	-0.004
rs1324003	13	4.3E+07	DNA meth	34470	Cardiomy	159811	T	C	0.004
rs1470217	3	6.7E+07	DNA meth	34470	Cardiomy	159811	T	C	-0.0102
rs2189234	4	1.1E+08	DNA meth	34470	Cardiomy	159811	G	T	-0.0037
rs3583606	2	1.1E+08	DNA meth	34470	Cardiomy	159811	G	A	0.0045
rs549280	4	7.5E+07	DNA meth	34470	Cardiomy	159811	A	G	-0.0041
rs5599822	11	7.4E+07	DNA meth	34470	Cardiomy	159811	G	A	0.0078
rs6069774	20	5.5E+07	DNA meth	34470	Cardiomy	159811	T	C	0.0035
rs6092873	9	9.1E+07	DNA meth	34470	Cardiomy	159811	C	T	0.0049
rs6979374	7	2.9E+07	DNA meth	34470	Cardiomy	159811	A	G	0.0036
rs7268313	8	1.1E+08	DNA meth	34470	Cardiomy	159811	G	A	-0.0056
rs7770095	6	4.1E+07	DNA meth	34470	Cardiomy	159811	A	G	-0.017
rs7857288	2	4.4E+07	DNA meth	34470	Cardiomy	159811	C	T	0.0071
rs8070454	17	3.8E+07	DNA meth	34470	Cardiomy	159811	T	C	0.0061
rs1160862	12	5.3E+07	DNA meth	34448	Hypertrop	218792	T	C	-590.41
rs1436732	8	1E+08	DNA meth	34448	Hypertrop	218792	G	A	-368.48
rs1486942	16	9E+07	DNA meth	34448	Hypertrop	218792	T	C	339.43
rs2046729	12	9.6E+07	DNA meth	34448	Hypertrop	218792	T	C	0.3916
rs2498857	9	1.1E+08	DNA meth	34448	Hypertrop	218792	C	T	0.3822
rs2851474	7	2288916	DNA meth	34448	Hypertrop	218792	C	T	1604.03
rs5617386	22	5.1E+07	DNA meth	34448	Hypertrop	218792	A	G	421.813

rs620671(	16	9E+07	DNA meth	34448	Hypertrop	218792	T	C	443.729
rs727863	21	4.3E+07	DNA meth	34448	Hypertrop	218792	G	T	-0.9444
rs749216	1	1.9E+07	DNA meth	34448	Hypertrop	218792	A	G	634.602
rs757809	4	1.9E+08	DNA meth	34448	Hypertrop	218792	A	G	-355.66
rs761465(	7	1.4E+08	DNA meth	34448	Hypertrop	218792	A	G	-439.48
rs782126(	22	5.1E+07	DNA meth	34448	Hypertrop	218792	T	C	347.26
rs788843	4	1.4E+08	DNA meth	34448	Hypertrop	218792	T	C	-373.26
rs115353(	5	7.2E+07	DNA meth	34470	Hypertrop	218792	T	C	0.0064
rs121888(	5	4.5E+07	DNA meth	34470	Hypertrop	218792	C	A	-0.0101
rs131336	4	3.8E+07	DNA meth	34470	Hypertrop	218792	C	T	-0.004
rs132400	13	4.3E+07	DNA meth	34470	Hypertrop	218792	T	C	0.004
rs147021	3	6.7E+07	DNA meth	34470	Hypertrop	218792	T	C	-0.0102
rs218923	4	1.1E+08	DNA meth	34470	Hypertrop	218792	G	T	-0.0037
rs358360(	2	1.1E+08	DNA meth	34470	Hypertrop	218792	G	A	0.0045
rs549280	4	7.5E+07	DNA meth	34470	Hypertrop	218792	A	G	-0.0041
rs559982	11	7.4E+07	DNA meth	34470	Hypertrop	218792	G	A	0.0078
rs606977	20	5.5E+07	DNA meth	34470	Hypertrop	218792	T	C	0.0035
rs609287	9	9.1E+07	DNA meth	34470	Hypertrop	218792	C	T	0.0049
rs697937	7	2.9E+07	DNA meth	34470	Hypertrop	218792	A	G	0.0036
rs726831	8	1.1E+08	DNA meth	34470	Hypertrop	218792	G	A	-0.0056
rs777009	6	4.1E+07	DNA meth	34470	Hypertrop	218792	A	G	-0.017
rs785728	2	4.4E+07	DNA meth	34470	Hypertrop	218792	C	T	0.0071
rs807045	17	3.8E+07	DNA meth	34470	Hypertrop	218792	T	C	0.0061
rs100527	10	3.8E+07	DNA meth	34449	Hypertrop	218792	C	A	-0.3006
rs107862	10	9.8E+07	DNA meth	34449	Hypertrop	218792	G	A	0.36
rs111901	10	1E+08	DNA meth	34449	Hypertrop	218792	T	C	0.147
rs114606	3	3.2E+07	DNA meth	34449	Hypertrop	218792	T	C	0.3554
rs115161	12	1.1E+07	DNA meth	34449	Hypertrop	218792	A	G	0.1773
rs120033	14	3.5E+07	DNA meth	34449	Hypertrop	218792	C	T	-0.179
rs121968	6	1.5E+08	DNA meth	34449	Hypertrop	218792	T	C	0.4614
rs122718(	11	7.8E+07	DNA meth	34449	Hypertrop	218792	A	G	0.364
rs124177	11	6.6E+07	DNA meth	34449	Hypertrop	218792	C	T	0.2093
rs148436	2	1.1E+08	DNA meth	34449	Hypertrop	218792	T	C	0.49
rs159885(	4	1E+08	DNA meth	34449	Hypertrop	218792	G	A	-0.1858
rs169584	17	9445045	DNA meth	34449	Hypertrop	218792	C	T	0.1533
rs184920	18	4.2E+07	DNA meth	34449	Hypertrop	218792	T	G	-0.1647
rs198496	14	9.6E+07	DNA meth	34449	Hypertrop	218792	G	A	-0.2047
rs201420	1	2.1E+08	DNA meth	34449	Hypertrop	218792	A	G	0.1696
rs219511	2	1.5E+08	DNA meth	34449	Hypertrop	218792	C	T	0.1614
rs264725	4	1.1E+08	DNA meth	34449	Hypertrop	218792	A	G	-0.1436
rs290805	7	1.3E+08	DNA meth	34449	Hypertrop	218792	T	C	-0.2268
rs309395(	6	3.1E+07	DNA meth	34449	Hypertrop	218792	C	T	0.2428

rs4141989	11	1E+08	DNA meth	34449	Hypertrop	218792	C	T	-0.8116
rs4679900	3	1.6E+08	DNA meth	34449	Hypertrop	218792	T	C	0.1538
rs4770473	13	2.4E+07	DNA meth	34449	Hypertrop	218792	T	C	0.159
rs4838595	10	5E+07	DNA meth	34449	Hypertrop	218792	T	C	-0.2576
rs6054829	20	7203816	DNA meth	34449	Hypertrop	218792	G	A	0.276
rs6238555	6	1.4E+07	DNA meth	34449	Hypertrop	218792	T	C	0.279
rs6440670	3	1.5E+08	DNA meth	34449	Hypertrop	218792	A	G	-0.1915
rs7017774	8	1340507	DNA meth	34449	Hypertrop	218792	T	C	0.146
rs7705526	5	1285974	DNA meth	34449	Hypertrop	218792	A	C	0.1811
rs7729949	7	4.9E+07	DNA meth	34449	Hypertrop	218792	C	T	0.7584
rs7871226	12	5.5E+07	DNA meth	34449	Hypertrop	218792	A	C	-0.231
rs7965945	12	2.5E+07	DNA meth	34449	Hypertrop	218792	C	T	0.2095
rs8005306	14	2.1E+07	DNA meth	34449	Hypertrop	218792	A	C	0.322
rs8019582	10	1.3E+08	DNA meth	34449	Hypertrop	218792	C	T	-0.2695
rs878175	6	1.4E+08	DNA meth	34449	Hypertrop	218792	C	T	0.1903
rs9813840	3	1.9E+08	DNA meth	34449	Hypertrop	218792	C	T	-0.4373
rs1084944	12	6493351	DNA meth	34463	Hypertrop	218792	G	A	-0.2346
rs1119012	10	1E+08	DNA meth	34463	Hypertrop	218792	A	C	0.2484
rs1121065	3	3.8E+07	DNA meth	34463	Hypertrop	218792	A	G	1.483
rs1124526	2	1.3E+08	DNA meth	34463	Hypertrop	218792	A	G	0.7517
rs1125333	10	759559	DNA meth	34463	Hypertrop	218792	T	C	0.2846
rs1142345	6	1.8E+07	DNA meth	34463	Hypertrop	218792	C	T	-0.8235
rs1168537	17	5.5E+07	DNA meth	34463	Hypertrop	218792	A	G	0.5517
rs1281881	12	8.2E+07	DNA meth	34463	Hypertrop	218792	T	C	0.2944
rs1487949	1	8895237	DNA meth	34463	Hypertrop	218792	A	G	-0.2109
rs1566295	17	7.1E+07	DNA meth	34463	Hypertrop	218792	C	T	-0.179
rs1670455	11	7.7E+07	DNA meth	34463	Hypertrop	218792	G	A	0.2007
rs1990053	7	4.5E+07	DNA meth	34463	Hypertrop	218792	A	G	0.2573
rs2300984	10	1E+08	DNA meth	34463	Hypertrop	218792	G	A	0.1833
rs2647266	4	1.1E+08	DNA meth	34463	Hypertrop	218792	G	A	0.176
rs375938	17	1.5E+07	DNA meth	34463	Hypertrop	218792	A	G	-0.1904
rs3829957	17	3378876	DNA meth	34463	Hypertrop	218792	T	C	-0.3796
rs4670518	2	3.6E+07	DNA meth	34463	Hypertrop	218792	A	C	0.1927
rs533852	3	1E+08	DNA meth	34463	Hypertrop	218792	T	C	-0.1741
rs5751180	22	2.3E+07	DNA meth	34463	Hypertrop	218792	T	C	-0.3915
rs6440670	3	1.5E+08	DNA meth	34463	Hypertrop	218792	A	G	-0.2251
rs6531114	2	1.7E+07	DNA meth	34463	Hypertrop	218792	C	T	0.2542
rs678553	1	2.4E+08	DNA meth	34463	Hypertrop	218792	C	T	-0.3265
rs7164338	15	7.8E+07	DNA meth	34463	Hypertrop	218792	C	T	-0.2031
rs7276556	2	1828908	DNA meth	34463	Hypertrop	218792	T	C	-0.9593
rs7302807	11	1.2E+08	DNA meth	34463	Hypertrop	218792	A	G	-0.4329
rs752223	1	6E+07	DNA meth	34463	Hypertrop	218792	A	G	-0.5602

rs767763‹	4	1.9E+08	DNA meth	34463	Hypertrop	218792	C	T	-0.2755
rs791391‹	10	3.1E+07	DNA meth	34463	Hypertrop	218792	T	C	0.1874
rs798208‹	1	1.6E+08	DNA meth	34463	Hypertrop	218792	C	T	0.9005
rs927121	20	5.1E+07	DNA meth	34463	Hypertrop	218792	C	T	-0.2149
rs938625‹	6	1.5E+08	DNA meth	34463	Hypertrop	218792	T	G	-0.372
rs940978‹	9	9.7E+07	DNA meth	34463	Hypertrop	218792	A	G	0.2691
rs987068‹	3	7.2E+07	DNA meth	34463	Hypertrop	218792	T	C	0.2221
rs115227‹	5	1.1E+08	DNA meth	34467	Hypertrop	218792	T	C	-0.5517
rs121167‹	1	1E+08	DNA meth	34467	Hypertrop	218792	T	C	-0.266
rs134126‹	2	1.1E+08	DNA meth	34467	Hypertrop	218792	G	A	-0.3915
rs138988‹	5	1.6E+08	DNA meth	34467	Hypertrop	218792	A	G	0.59
rs148545‹	6	1.5E+08	DNA meth	34467	Hypertrop	218792	T	C	1.0581
rs169362‹	10	8E+07	DNA meth	34467	Hypertrop	218792	C	T	-0.1908
rs170941‹	10	1E+08	DNA meth	34467	Hypertrop	218792	G	A	0.18
rs172798‹	15	8E+07	DNA meth	34467	Hypertrop	218792	A	G	-0.1737
rs180044‹	2	3.8E+07	DNA meth	34467	Hypertrop	218792	C	T	0.1834
rs183718‹	2	2E+08	DNA meth	34467	Hypertrop	218792	C	T	0.1641
rs201005‹	3	1.3E+08	DNA meth	34467	Hypertrop	218792	G	A	0.1456
rs381736‹	3	1.6E+08	DNA meth	34467	Hypertrop	218792	G	T	-0.2347
rs406532‹	17	3.8E+07	DNA meth	34467	Hypertrop	218792	T	C	-0.1703
rs670929‹	2	1.6E+08	DNA meth	34467	Hypertrop	218792	T	G	0.151
rs688653‹	5	1.5E+08	DNA meth	34467	Hypertrop	218792	T	C	-0.3752
rs726597‹	13	1E+08	DNA meth	34467	Hypertrop	218792	T	C	0.2962
rs761833‹	11	1.2E+07	DNA meth	34467	Hypertrop	218792	T	C	0.2802
rs773853	19	1.7E+07	DNA meth	34467	Hypertrop	218792	G	A	-0.174
rs774886‹	4	1.9E+08	DNA meth	34467	Hypertrop	218792	C	T	0.2283
rs790051	2	2.3E+08	DNA meth	34467	Hypertrop	218792	A	G	-0.1676
rs791923‹	10	1.8E+07	DNA meth	34467	Hypertrop	218792	C	T	0.1787
rs887466	6	3.1E+07	DNA meth	34467	Hypertrop	218792	A	G	-0.1928
rs938679‹	6	1.1E+08	DNA meth	34467	Hypertrop	218792	T	C	0.1983
rs979067‹	4	1.1E+08	DNA meth	34467	Hypertrop	218792	G	A	-0.2246
rs108494‹	12	6493351	DNA meth	34463	Hypertens	218754	G	A	-0.2346
rs111901‹	10	1E+08	DNA meth	34463	Hypertens	218754	A	C	0.2484
rs112106‹	3	3.8E+07	DNA meth	34463	Hypertens	218754	A	G	1.483
rs112452‹	2	1.3E+08	DNA meth	34463	Hypertens	218754	A	G	0.7517
rs112533‹	10	759559	DNA meth	34463	Hypertens	218754	T	C	0.2846
rs114234‹	6	1.8E+07	DNA meth	34463	Hypertens	218754	C	T	-0.8235
rs116853‹	17	5.5E+07	DNA meth	34463	Hypertens	218754	A	G	0.5517
rs128188‹	12	8.2E+07	DNA meth	34463	Hypertens	218754	T	C	0.2944
rs148794‹	1	8895237	DNA meth	34463	Hypertens	218754	A	G	-0.2109
rs156629‹	17	7.1E+07	DNA meth	34463	Hypertens	218754	C	T	-0.179
rs167045‹	11	7.7E+07	DNA meth	34463	Hypertens	218754	G	A	0.2007

rs199005	7	4.5E+07	DNA meth	34463	Hypertens	218754	A	G	0.2573
rs230098	10	1E+08	DNA meth	34463	Hypertens	218754	G	A	0.1833
rs264726	4	1.1E+08	DNA meth	34463	Hypertens	218754	G	A	0.176
rs375938	17	1.5E+07	DNA meth	34463	Hypertens	218754	A	G	-0.1904
rs382995	17	3378876	DNA meth	34463	Hypertens	218754	T	C	-0.3796
rs467051	2	3.6E+07	DNA meth	34463	Hypertens	218754	A	C	0.1927
rs533852	3	1E+08	DNA meth	34463	Hypertens	218754	T	C	-0.1741
rs575118	22	2.3E+07	DNA meth	34463	Hypertens	218754	T	C	-0.3915
rs644067	3	1.5E+08	DNA meth	34463	Hypertens	218754	A	G	-0.2251
rs653111	2	1.7E+07	DNA meth	34463	Hypertens	218754	C	T	0.2542
rs678553	1	2.4E+08	DNA meth	34463	Hypertens	218754	C	T	-0.3265
rs716433	15	7.8E+07	DNA meth	34463	Hypertens	218754	C	T	-0.2031
rs727655	2	1828908	DNA meth	34463	Hypertens	218754	T	C	-0.9593
rs730280	11	1.2E+08	DNA meth	34463	Hypertens	218754	A	G	-0.4329
rs752223	1	6E+07	DNA meth	34463	Hypertens	218754	A	G	-0.5602
rs767763	4	1.9E+08	DNA meth	34463	Hypertens	218754	C	T	-0.2755
rs791391	10	3.1E+07	DNA meth	34463	Hypertens	218754	T	C	0.1874
rs798208	1	1.6E+08	DNA meth	34463	Hypertens	218754	C	T	0.9005
rs927121	20	5.1E+07	DNA meth	34463	Hypertens	218754	C	T	-0.2149
rs938625	6	1.5E+08	DNA meth	34463	Hypertens	218754	T	G	-0.372
rs940978	9	9.7E+07	DNA meth	34463	Hypertens	218754	A	G	0.2691
rs987068	3	7.2E+07	DNA meth	34463	Hypertens	218754	T	C	0.2221
rs115353	5	7.2E+07	DNA meth	34470	Hypertens	218754	T	C	0.0064
rs121888	5	4.5E+07	DNA meth	34470	Hypertens	218754	C	A	-0.0101
rs131336	4	3.8E+07	DNA meth	34470	Hypertens	218754	C	T	-0.004
rs132400	13	4.3E+07	DNA meth	34470	Hypertens	218754	T	C	0.004
rs147021	3	6.7E+07	DNA meth	34470	Hypertens	218754	T	C	-0.0102
rs218923	4	1.1E+08	DNA meth	34470	Hypertens	218754	G	T	-0.0037
rs358360	2	1.1E+08	DNA meth	34470	Hypertens	218754	G	A	0.0045
rs549280	4	7.5E+07	DNA meth	34470	Hypertens	218754	A	G	-0.0041
rs559982	11	7.4E+07	DNA meth	34470	Hypertens	218754	G	A	0.0078
rs606977	20	5.5E+07	DNA meth	34470	Hypertens	218754	T	C	0.0035
rs609287	9	9.1E+07	DNA meth	34470	Hypertens	218754	C	T	0.0049
rs697937	7	2.9E+07	DNA meth	34470	Hypertens	218754	A	G	0.0036
rs726831	8	1.1E+08	DNA meth	34470	Hypertens	218754	G	A	-0.0056
rs777009	6	4.1E+07	DNA meth	34470	Hypertens	218754	A	G	-0.017
rs785728	2	4.4E+07	DNA meth	34470	Hypertens	218754	C	T	0.0071
rs807045	17	3.8E+07	DNA meth	34470	Hypertens	218754	T	C	0.0061
rs100527	10	3.8E+07	DNA meth	34449	Hypertens	218754	C	A	-0.3006
rs107862	10	9.8E+07	DNA meth	34449	Hypertens	218754	G	A	0.36
rs111901	10	1E+08	DNA meth	34449	Hypertens	218754	T	C	0.147
rs114606	3	3.2E+07	DNA meth	34449	Hypertens	218754	T	C	0.3554

rs115161	12	1.1E+07	DNA meth	34449	Hypertens	218754	A	G	0.1773
rs120033	14	3.5E+07	DNA meth	34449	Hypertens	218754	C	T	-0.179
rs121968	6	1.5E+08	DNA meth	34449	Hypertens	218754	T	C	0.4614
rs122718	11	7.8E+07	DNA meth	34449	Hypertens	218754	A	G	0.364
rs124177	11	6.6E+07	DNA meth	34449	Hypertens	218754	C	T	0.2093
rs148436	2	1.1E+08	DNA meth	34449	Hypertens	218754	T	C	0.49
rs159885	4	1E+08	DNA meth	34449	Hypertens	218754	G	A	-0.1858
rs169584	17	9445045	DNA meth	34449	Hypertens	218754	C	T	0.1533
rs184920	18	4.2E+07	DNA meth	34449	Hypertens	218754	T	G	-0.1647
rs198496	14	9.6E+07	DNA meth	34449	Hypertens	218754	G	A	-0.2047
rs201420	1	2.1E+08	DNA meth	34449	Hypertens	218754	A	G	0.1696
rs219511	2	1.5E+08	DNA meth	34449	Hypertens	218754	C	T	0.1614
rs264725	4	1.1E+08	DNA meth	34449	Hypertens	218754	A	G	-0.1436
rs290805	7	1.3E+08	DNA meth	34449	Hypertens	218754	T	C	-0.2268
rs309395	6	3.1E+07	DNA meth	34449	Hypertens	218754	C	T	0.2428
rs414198	11	1E+08	DNA meth	34449	Hypertens	218754	C	T	-0.8116
rs467990	3	1.6E+08	DNA meth	34449	Hypertens	218754	T	C	0.1538
rs477047	13	2.4E+07	DNA meth	34449	Hypertens	218754	T	C	0.159
rs483859	10	5E+07	DNA meth	34449	Hypertens	218754	T	C	-0.2576
rs605482	20	7203816	DNA meth	34449	Hypertens	218754	G	A	0.276
rs623855	6	1.4E+07	DNA meth	34449	Hypertens	218754	T	C	0.279
rs644067	3	1.5E+08	DNA meth	34449	Hypertens	218754	A	G	-0.1915
rs701777	8	1340507	DNA meth	34449	Hypertens	218754	T	C	0.146
rs770552	5	1285974	DNA meth	34449	Hypertens	218754	A	C	0.1811
rs772994	7	4.9E+07	DNA meth	34449	Hypertens	218754	C	T	0.7584
rs787122	12	5.5E+07	DNA meth	34449	Hypertens	218754	A	C	-0.231
rs796594	12	2.5E+07	DNA meth	34449	Hypertens	218754	C	T	0.2095
rs800530	14	2.1E+07	DNA meth	34449	Hypertens	218754	A	C	0.322
rs801958	10	1.3E+08	DNA meth	34449	Hypertens	218754	C	T	-0.2695
rs878175	6	1.4E+08	DNA meth	34449	Hypertens	218754	C	T	0.1903
rs981384	3	1.9E+08	DNA meth	34449	Hypertens	218754	C	T	-0.4373
rs115227	5	1.1E+08	DNA meth	34467	Hypertens	218754	T	C	-0.5517
rs121167	1	1E+08	DNA meth	34467	Hypertens	218754	T	C	-0.266
rs134126	2	1.1E+08	DNA meth	34467	Hypertens	218754	G	A	-0.3915
rs138988	5	1.6E+08	DNA meth	34467	Hypertens	218754	A	G	0.59
rs148545	6	1.5E+08	DNA meth	34467	Hypertens	218754	T	C	1.0581
rs169362	10	8E+07	DNA meth	34467	Hypertens	218754	C	T	-0.1908
rs170941	10	1E+08	DNA meth	34467	Hypertens	218754	G	A	0.18
rs172798	15	8E+07	DNA meth	34467	Hypertens	218754	A	G	-0.1737
rs180044	2	3.8E+07	DNA meth	34467	Hypertens	218754	C	T	0.1834
rs183718	2	2E+08	DNA meth	34467	Hypertens	218754	C	T	0.1641
rs201005	3	1.3E+08	DNA meth	34467	Hypertens	218754	G	A	0.1456

rs381736[illegible]	3	1.6E+08	DNA meth	34467	Hypertens	218754	G	T	-0.2347
rs406532[illegible]	17	3.8E+07	DNA meth	34467	Hypertens	218754	T	C	-0.1703
rs670929[illegible]	2	1.6E+08	DNA meth	34467	Hypertens	218754	T	G	0.151
rs688653[illegible]	5	1.5E+08	DNA meth	34467	Hypertens	218754	T	C	-0.3752
rs726597[illegible]	13	1E+08	DNA meth	34467	Hypertens	218754	T	C	0.2962
rs761833[illegible]	11	1.2E+07	DNA meth	34467	Hypertens	218754	T	C	0.2802
rs773853	19	1.7E+07	DNA meth	34467	Hypertens	218754	G	A	-0.174
rs774886[illegible]	4	1.9E+08	DNA meth	34467	Hypertens	218754	C	T	0.2283
rs790051	2	2.3E+08	DNA meth	34467	Hypertens	218754	A	G	-0.1676
rs791923[illegible]	10	1.8E+07	DNA meth	34467	Hypertens	218754	C	T	0.1787
rs887466	6	3.1E+07	DNA meth	34467	Hypertens	218754	A	G	-0.1928
rs938679[illegible]	6	1.1E+08	DNA meth	34467	Hypertens	218754	T	C	0.1983
rs979067[illegible]	4	1.1E+08	DNA meth	34467	Hypertens	218754	G	A	-0.2246
rs116086[illegible]	12	5.3E+07	DNA meth	34448	Hypertens	218754	T	C	-590.41
rs143673[illegible]	8	1E+08	DNA meth	34448	Hypertens	218754	G	A	-368.48
rs148694[illegible]	16	9E+07	DNA meth	34448	Hypertens	218754	T	C	339.43
rs204672[illegible]	12	9.6E+07	DNA meth	34448	Hypertens	218754	T	C	0.3916
rs249885[illegible]	9	1.1E+08	DNA meth	34448	Hypertens	218754	C	T	0.3822
rs285147[illegible]	7	2288916	DNA meth	34448	Hypertens	218754	C	T	1604.03
rs561738[illegible]	22	5.1E+07	DNA meth	34448	Hypertens	218754	A	G	421.813
rs620671[illegible]	16	9E+07	DNA meth	34448	Hypertens	218754	T	C	443.729
rs727863[illegible]	21	4.3E+07	DNA meth	34448	Hypertens	218754	G	T	-0.9444
rs749216[illegible]	1	1.9E+07	DNA meth	34448	Hypertens	218754	A	G	634.602
rs757809[illegible]	4	1.9E+08	DNA meth	34448	Hypertens	218754	A	G	-355.66
rs761465[illegible]	7	1.4E+08	DNA meth	34448	Hypertens	218754	A	G	-439.48
rs782126[illegible]	22	5.1E+07	DNA meth	34448	Hypertens	218754	T	C	347.26
rs788843[illegible]	4	1.4E+08	DNA meth	34448	Hypertens	218754	T	C	-373.26
rs100527[illegible]	10	3.8E+07	DNA meth	34449	Ischemic I	218792	C	A	-0.3006
rs107862[illegible]	10	9.8E+07	DNA meth	34449	Ischemic I	218792	G	A	0.36
rs111901[illegible]	10	1E+08	DNA meth	34449	Ischemic I	218792	T	C	0.147
rs114606[illegible]	3	3.2E+07	DNA meth	34449	Ischemic I	218792	T	C	0.3554
rs115161[illegible]	12	1.1E+07	DNA meth	34449	Ischemic I	218792	A	G	0.1773
rs120033[illegible]	14	3.5E+07	DNA meth	34449	Ischemic I	218792	C	T	-0.179
rs121968[illegible]	6	1.5E+08	DNA meth	34449	Ischemic I	218792	T	C	0.4614
rs122718[illegible]	11	7.8E+07	DNA meth	34449	Ischemic I	218792	A	G	0.364
rs124177[illegible]	11	6.6E+07	DNA meth	34449	Ischemic I	218792	C	T	0.2093
rs148436[illegible]	2	1.1E+08	DNA meth	34449	Ischemic I	218792	T	C	0.49
rs159885[illegible]	4	1E+08	DNA meth	34449	Ischemic I	218792	G	A	-0.1858
rs169584[illegible]	17	9445045	DNA meth	34449	Ischemic I	218792	C	T	0.1533
rs184920[illegible]	18	4.2E+07	DNA meth	34449	Ischemic I	218792	T	G	-0.1647
rs198496[illegible]	14	9.6E+07	DNA meth	34449	Ischemic I	218792	G	A	-0.2047
rs201420[illegible]	1	2.1E+08	DNA meth	34449	Ischemic I	218792	A	G	0.1696

rs2195114	2	1.5E+08	DNA meth	34449	Ischemic h	218792	C	T	0.1614
rs2647254	4	1.1E+08	DNA meth	34449	Ischemic h	218792	A	G	-0.1436
rs290805	7	1.3E+08	DNA meth	34449	Ischemic h	218792	T	C	-0.2268
rs3093956	6	3.1E+07	DNA meth	34449	Ischemic h	218792	C	T	0.2428
rs4141989	11	1E+08	DNA meth	34449	Ischemic h	218792	C	T	-0.8116
rs4679900	3	1.6E+08	DNA meth	34449	Ischemic h	218792	T	C	0.1538
rs4770473	13	2.4E+07	DNA meth	34449	Ischemic h	218792	T	C	0.159
rs4838595	10	5E+07	DNA meth	34449	Ischemic h	218792	T	C	-0.2576
rs6054829	20	7203816	DNA meth	34449	Ischemic h	218792	G	A	0.276
rs6238555	6	1.4E+07	DNA meth	34449	Ischemic h	218792	T	C	0.279
rs6440670	3	1.5E+08	DNA meth	34449	Ischemic h	218792	A	G	-0.1915
rs7017774	8	1340507	DNA meth	34449	Ischemic h	218792	T	C	0.146
rs7705526	5	1285974	DNA meth	34449	Ischemic h	218792	A	C	0.1811
rs7729949	7	4.9E+07	DNA meth	34449	Ischemic h	218792	C	T	0.7584
rs7871226	12	5.5E+07	DNA meth	34449	Ischemic h	218792	A	C	-0.231
rs7965945	12	2.5E+07	DNA meth	34449	Ischemic h	218792	C	T	0.2095
rs8005306	14	2.1E+07	DNA meth	34449	Ischemic h	218792	A	C	0.322
rs8019582	10	1.3E+08	DNA meth	34449	Ischemic h	218792	C	T	-0.2695
rs878175	6	1.4E+08	DNA meth	34449	Ischemic h	218792	C	T	0.1903
rs9813840	3	1.9E+08	DNA meth	34449	Ischemic h	218792	C	T	-0.4373
rs1084944	12	6493351	DNA meth	34463	Ischemic h	218792	G	A	-0.2346
rs1119012	10	1E+08	DNA meth	34463	Ischemic h	218792	A	C	0.2484
rs1121065	3	3.8E+07	DNA meth	34463	Ischemic h	218792	A	G	1.483
rs1124526	2	1.3E+08	DNA meth	34463	Ischemic h	218792	A	G	0.7517
rs1125333	10	759559	DNA meth	34463	Ischemic h	218792	T	C	0.2846
rs1142345	6	1.8E+07	DNA meth	34463	Ischemic h	218792	C	T	-0.8235
rs1168537	17	5.5E+07	DNA meth	34463	Ischemic h	218792	A	G	0.5517
rs1281881	12	8.2E+07	DNA meth	34463	Ischemic h	218792	T	C	0.2944
rs1487949	1	8895237	DNA meth	34463	Ischemic h	218792	A	G	-0.2109
rs1566295	17	7.1E+07	DNA meth	34463	Ischemic h	218792	C	T	-0.179
rs1670455	11	7.7E+07	DNA meth	34463	Ischemic h	218792	G	A	0.2007
rs1990053	7	4.5E+07	DNA meth	34463	Ischemic h	218792	A	G	0.2573
rs2300984	10	1E+08	DNA meth	34463	Ischemic h	218792	G	A	0.1833
rs2647266	4	1.1E+08	DNA meth	34463	Ischemic h	218792	G	A	0.176
rs375938	17	1.5E+07	DNA meth	34463	Ischemic h	218792	A	G	-0.1904
rs3829957	17	3378876	DNA meth	34463	Ischemic h	218792	T	C	-0.3796
rs4670518	2	3.6E+07	DNA meth	34463	Ischemic h	218792	A	C	0.1927
rs533852	3	1E+08	DNA meth	34463	Ischemic h	218792	T	C	-0.1741
rs5751180	22	2.3E+07	DNA meth	34463	Ischemic h	218792	T	C	-0.3915
rs6440670	3	1.5E+08	DNA meth	34463	Ischemic h	218792	A	G	-0.2251
rs6531114	2	1.7E+07	DNA meth	34463	Ischemic h	218792	C	T	0.2542
rs678553	1	2.4E+08	DNA meth	34463	Ischemic h	218792	C	T	-0.3265

rs7164338	15	7.8E+07	DNA meth	34463	Ischemic I	218792	C	T	-0.2031
rs7276556	2	1828908	DNA meth	34463	Ischemic I	218792	T	C	-0.9593
rs7302807	11	1.2E+08	DNA meth	34463	Ischemic I	218792	A	G	-0.4329
rs752223	1	6E+07	DNA meth	34463	Ischemic I	218792	A	G	-0.5602
rs7677639	4	1.9E+08	DNA meth	34463	Ischemic I	218792	C	T	-0.2755
rs7913917	10	3.1E+07	DNA meth	34463	Ischemic I	218792	T	C	0.1874
rs7982088	1	1.6E+08	DNA meth	34463	Ischemic I	218792	C	T	0.9005
rs927121	20	5.1E+07	DNA meth	34463	Ischemic I	218792	C	T	-0.2149
rs9386256	6	1.5E+08	DNA meth	34463	Ischemic I	218792	T	G	-0.372
rs9409787	9	9.7E+07	DNA meth	34463	Ischemic I	218792	A	G	0.2691
rs9870681	3	7.2E+07	DNA meth	34463	Ischemic I	218792	T	C	0.2221
rs1160862	12	5.3E+07	DNA meth	34448	Ischemic I	218792	T	C	-590.41
rs1436732	8	1E+08	DNA meth	34448	Ischemic I	218792	G	A	-368.48
rs1486942	16	9E+07	DNA meth	34448	Ischemic I	218792	T	C	339.43
rs2046729	12	9.6E+07	DNA meth	34448	Ischemic I	218792	T	C	0.3916
rs2498857	9	1.1E+08	DNA meth	34448	Ischemic I	218792	C	T	0.3822
rs2851474	7	2288916	DNA meth	34448	Ischemic I	218792	C	T	1604.03
rs5617386	22	5.1E+07	DNA meth	34448	Ischemic I	218792	A	G	421.813
rs6206710	16	9E+07	DNA meth	34448	Ischemic I	218792	T	C	443.729
rs7278637	21	4.3E+07	DNA meth	34448	Ischemic I	218792	G	T	-0.9444
rs7492167	1	1.9E+07	DNA meth	34448	Ischemic I	218792	A	G	634.602
rs7578093	4	1.9E+08	DNA meth	34448	Ischemic I	218792	A	G	-355.66
rs7614650	7	1.4E+08	DNA meth	34448	Ischemic I	218792	A	G	-439.48
rs7821260	22	5.1E+07	DNA meth	34448	Ischemic I	218792	T	C	347.26
rs7888438	4	1.4E+08	DNA meth	34448	Ischemic I	218792	T	C	-373.26
rs1152271	5	1.1E+08	DNA meth	34467	Ischemic I	218792	T	C	-0.5517
rs1211671	1	1E+08	DNA meth	34467	Ischemic I	218792	T	C	-0.266
rs1341264	2	1.1E+08	DNA meth	34467	Ischemic I	218792	G	A	-0.3915
rs1389887	5	1.6E+08	DNA meth	34467	Ischemic I	218792	A	G	0.59
rs1485456	6	1.5E+08	DNA meth	34467	Ischemic I	218792	T	C	1.0581
rs1693623	10	8E+07	DNA meth	34467	Ischemic I	218792	C	T	-0.1908
rs1709414	10	1E+08	DNA meth	34467	Ischemic I	218792	G	A	0.18
rs1727988	15	8E+07	DNA meth	34467	Ischemic I	218792	A	G	-0.1737
rs1800440	2	3.8E+07	DNA meth	34467	Ischemic I	218792	C	T	0.1834
rs1837188	2	2E+08	DNA meth	34467	Ischemic I	218792	C	T	0.1641
rs2010054	3	1.3E+08	DNA meth	34467	Ischemic I	218792	G	A	0.1456
rs3817367	3	1.6E+08	DNA meth	34467	Ischemic I	218792	G	T	-0.2347
rs4065321	17	3.8E+07	DNA meth	34467	Ischemic I	218792	T	C	-0.1703
rs6709296	2	1.6E+08	DNA meth	34467	Ischemic I	218792	T	G	0.151
rs6886532	5	1.5E+08	DNA meth	34467	Ischemic I	218792	T	C	-0.3752
rs7265977	13	1E+08	DNA meth	34467	Ischemic I	218792	T	C	0.2962
rs7618337	11	1.2E+07	DNA meth	34467	Ischemic I	218792	T	C	0.2802

rs773853	19	1.7E+07	DNA meth	34467	Ischemic	218792	G	A	-0.174
rs774886	4	1.9E+08	DNA meth	34467	Ischemic	218792	C	T	0.2283
rs790051	2	2.3E+08	DNA meth	34467	Ischemic	218792	A	G	-0.1676
rs791923	10	1.8E+07	DNA meth	34467	Ischemic	218792	C	T	0.1787
rs887466	6	3.1E+07	DNA meth	34467	Ischemic	218792	A	G	-0.1928
rs938679	6	1.1E+08	DNA meth	34467	Ischemic	218792	T	C	0.1983
rs979067	4	1.1E+08	DNA meth	34467	Ischemic	218792	G	A	-0.2246
rs115353	5	7.2E+07	DNA meth	34470	Ischemic	218792	T	C	0.0064
rs121888	5	4.5E+07	DNA meth	34470	Ischemic	218792	C	A	-0.0101
rs131336	4	3.8E+07	DNA meth	34470	Ischemic	218792	C	T	-0.004
rs132400	13	4.3E+07	DNA meth	34470	Ischemic	218792	T	C	0.004
rs147021	3	6.7E+07	DNA meth	34470	Ischemic	218792	T	C	-0.0102
rs218923	4	1.1E+08	DNA meth	34470	Ischemic	218792	G	T	-0.0037
rs358360	2	1.1E+08	DNA meth	34470	Ischemic	218792	G	A	0.0045
rs549280	4	7.5E+07	DNA meth	34470	Ischemic	218792	A	G	-0.0041
rs559982	11	7.4E+07	DNA meth	34470	Ischemic	218792	G	A	0.0078
rs606977	20	5.5E+07	DNA meth	34470	Ischemic	218792	T	C	0.0035
rs609287	9	9.1E+07	DNA meth	34470	Ischemic	218792	C	T	0.0049
rs697937	7	2.9E+07	DNA meth	34470	Ischemic	218792	A	G	0.0036
rs726831	8	1.1E+08	DNA meth	34470	Ischemic	218792	G	A	-0.0056
rs777009	6	4.1E+07	DNA meth	34470	Ischemic	218792	A	G	-0.017
rs785728	2	4.4E+07	DNA meth	34470	Ischemic	218792	C	T	0.0071
rs807045	17	3.8E+07	DNA meth	34470	Ischemic	218792	T	C	0.0061
rs116086	12	5.3E+07	DNA meth	34448	Non-ische	187152	T	C	-590.41
rs143673	8	1E+08	DNA meth	34448	Non-ische	187152	G	A	-368.48
rs148694	16	9E+07	DNA meth	34448	Non-ische	187152	T	C	339.43
rs204672	12	9.6E+07	DNA meth	34448	Non-ische	187152	T	C	0.3916
rs249885	9	1.1E+08	DNA meth	34448	Non-ische	187152	C	T	0.3822
rs285147	7	2288916	DNA meth	34448	Non-ische	187152	C	T	1604.03
rs561738	22	5.1E+07	DNA meth	34448	Non-ische	187152	A	G	421.813
rs620671	16	9E+07	DNA meth	34448	Non-ische	187152	T	C	443.729
rs727863	21	4.3E+07	DNA meth	34448	Non-ische	187152	G	T	-0.9444
rs749216	1	1.9E+07	DNA meth	34448	Non-ische	187152	A	G	634.602
rs757809	4	1.9E+08	DNA meth	34448	Non-ische	187152	A	G	-355.66
rs761465	7	1.4E+08	DNA meth	34448	Non-ische	187152	A	G	-439.48
rs782126	22	5.1E+07	DNA meth	34448	Non-ische	187152	T	C	347.26
rs788843	4	1.4E+08	DNA meth	34448	Non-ische	187152	T	C	-373.26
rs100527	10	3.8E+07	DNA meth	34449	Non-ische	187152	C	A	-0.3006
rs107862	10	9.8E+07	DNA meth	34449	Non-ische	187152	G	A	0.36
rs111901	10	1E+08	DNA meth	34449	Non-ische	187152	T	C	0.147
rs114606	3	3.2E+07	DNA meth	34449	Non-ische	187152	T	C	0.3554
rs115161	12	1.1E+07	DNA meth	34449	Non-ische	187152	A	G	0.1773

rs120033:	14	3.5E+07	DNA meth	34449	Non-ische	187152	C	T	-0.179
rs121968:	6	1.5E+08	DNA meth	34449	Non-ische	187152	T	C	0.4614
rs122718(	11	7.8E+07	DNA meth	34449	Non-ische	187152	A	G	0.364
rs124177!	11	6.6E+07	DNA meth	34449	Non-ische	187152	C	T	0.2093
rs148436:	2	1.1E+08	DNA meth	34449	Non-ische	187152	T	C	0.49
rs159885(	4	1E+08	DNA meth	34449	Non-ische	187152	G	A	-0.1858
rs169584!	17	9445045	DNA meth	34449	Non-ische	187152	C	T	0.1533
rs184920!	18	4.2E+07	DNA meth	34449	Non-ische	187152	T	G	-0.1647
rs198496!	14	9.6E+07	DNA meth	34449	Non-ische	187152	G	A	-0.2047
rs201420:	1	2.1E+08	DNA meth	34449	Non-ische	187152	A	G	0.1696
rs219511	2	1.5E+08	DNA meth	34449	Non-ische	187152	C	T	0.1614
rs264725	4	1.1E+08	DNA meth	34449	Non-ische	187152	A	G	-0.1436
rs290805	7	1.3E+08	DNA meth	34449	Non-ische	187152	T	C	-0.2268
rs309395(	6	3.1E+07	DNA meth	34449	Non-ische	187152	C	T	0.2428
rs414198!	11	1E+08	DNA meth	34449	Non-ische	187152	C	T	-0.8116
rs467990(	3	1.6E+08	DNA meth	34449	Non-ische	187152	T	C	0.1538
rs477047:	13	2.4E+07	DNA meth	34449	Non-ische	187152	T	C	0.159
rs483859!	10	5E+07	DNA meth	34449	Non-ische	187152	T	C	-0.2576
rs605482!	20	7203816	DNA meth	34449	Non-ische	187152	G	A	0.276
rs623855!	6	1.4E+07	DNA meth	34449	Non-ische	187152	T	C	0.279
rs644067(	3	1.5E+08	DNA meth	34449	Non-ische	187152	A	G	-0.1915
rs701777	8	1340507	DNA meth	34449	Non-ische	187152	T	C	0.146
rs770552(	5	1285974	DNA meth	34449	Non-ische	187152	A	C	0.1811
rs772994!	7	4.9E+07	DNA meth	34449	Non-ische	187152	C	T	0.7584
rs787122(	12	5.5E+07	DNA meth	34449	Non-ische	187152	A	C	-0.231
rs796594!	12	2.5E+07	DNA meth	34449	Non-ische	187152	C	T	0.2095
rs800530(	14	2.1E+07	DNA meth	34449	Non-ische	187152	A	C	0.322
rs801958:	10	1.3E+08	DNA meth	34449	Non-ische	187152	C	T	-0.2695
rs878175	6	1.4E+08	DNA meth	34449	Non-ische	187152	C	T	0.1903
rs981384(	3	1.9E+08	DNA meth	34449	Non-ische	187152	C	T	-0.4373
rs115353(	5	7.2E+07	DNA meth	34470	Non-ische	187152	T	C	0.0064
rs121888(	5	4.5E+07	DNA meth	34470	Non-ische	187152	C	A	-0.0101
rs131336	4	3.8E+07	DNA meth	34470	Non-ische	187152	C	T	-0.004
rs132400:	13	4.3E+07	DNA meth	34470	Non-ische	187152	T	C	0.004
rs147021	3	6.7E+07	DNA meth	34470	Non-ische	187152	T	C	-0.0102
rs218923	4	1.1E+08	DNA meth	34470	Non-ische	187152	G	T	-0.0037
rs358360(	2	1.1E+08	DNA meth	34470	Non-ische	187152	G	A	0.0045
rs549280	4	7.5E+07	DNA meth	34470	Non-ische	187152	A	G	-0.0041
rs559982:	11	7.4E+07	DNA meth	34470	Non-ische	187152	G	A	0.0078
rs606977	20	5.5E+07	DNA meth	34470	Non-ische	187152	T	C	0.0035
rs609287:	9	9.1E+07	DNA meth	34470	Non-ische	187152	C	T	0.0049
rs697937	7	2.9E+07	DNA meth	34470	Non-ische	187152	A	G	0.0036

rs726831	8	1.1E+08	DNA meth	34470	Non-ische	187152	G	A	-0.0056
rs777009	6	4.1E+07	DNA meth	34470	Non-ische	187152	A	G	-0.017
rs785728	2	4.4E+07	DNA meth	34470	Non-ische	187152	C	T	0.0071
rs807045	17	3.8E+07	DNA meth	34470	Non-ische	187152	T	C	0.0061
rs115227	5	1.1E+08	DNA meth	34467	Non-ische	187152	T	C	-0.5517
rs121167	1	1E+08	DNA meth	34467	Non-ische	187152	T	C	-0.266
rs134126	2	1.1E+08	DNA meth	34467	Non-ische	187152	G	A	-0.3915
rs138988	5	1.6E+08	DNA meth	34467	Non-ische	187152	A	G	0.59
rs148545	6	1.5E+08	DNA meth	34467	Non-ische	187152	T	C	1.0581
rs169362	10	8E+07	DNA meth	34467	Non-ische	187152	C	T	-0.1908
rs170941	10	1E+08	DNA meth	34467	Non-ische	187152	G	A	0.18
rs172798	15	8E+07	DNA meth	34467	Non-ische	187152	A	G	-0.1737
rs180044	2	3.8E+07	DNA meth	34467	Non-ische	187152	C	T	0.1834
rs183718	2	2E+08	DNA meth	34467	Non-ische	187152	C	T	0.1641
rs201005	3	1.3E+08	DNA meth	34467	Non-ische	187152	G	A	0.1456
rs381736	3	1.6E+08	DNA meth	34467	Non-ische	187152	G	T	-0.2347
rs406532	17	3.8E+07	DNA meth	34467	Non-ische	187152	T	C	-0.1703
rs670929	2	1.6E+08	DNA meth	34467	Non-ische	187152	T	G	0.151
rs688653	5	1.5E+08	DNA meth	34467	Non-ische	187152	T	C	-0.3752
rs726597	13	1E+08	DNA meth	34467	Non-ische	187152	T	C	0.2962
rs761833	11	1.2E+07	DNA meth	34467	Non-ische	187152	T	C	0.2802
rs773853	19	1.7E+07	DNA meth	34467	Non-ische	187152	G	A	-0.174
rs774886	4	1.9E+08	DNA meth	34467	Non-ische	187152	C	T	0.2283
rs790051	2	2.3E+08	DNA meth	34467	Non-ische	187152	A	G	-0.1676
rs791923	10	1.8E+07	DNA meth	34467	Non-ische	187152	C	T	0.1787
rs887466	6	3.1E+07	DNA meth	34467	Non-ische	187152	A	G	-0.1928
rs938679	6	1.1E+08	DNA meth	34467	Non-ische	187152	T	C	0.1983
rs979067	4	1.1E+08	DNA meth	34467	Non-ische	187152	G	A	-0.2246
rs108494	12	6493351	DNA meth	34463	Non-ische	187152	G	A	-0.2346
rs111901	10	1E+08	DNA meth	34463	Non-ische	187152	A	C	0.2484
rs112106	3	3.8E+07	DNA meth	34463	Non-ische	187152	A	G	1.483
rs112452	2	1.3E+08	DNA meth	34463	Non-ische	187152	A	G	0.7517
rs112533	10	759559	DNA meth	34463	Non-ische	187152	T	C	0.2846
rs114234	6	1.8E+07	DNA meth	34463	Non-ische	187152	C	T	-0.8235
rs116853	17	5.5E+07	DNA meth	34463	Non-ische	187152	A	G	0.5517
rs128188	12	8.2E+07	DNA meth	34463	Non-ische	187152	T	C	0.2944
rs148794	1	8895237	DNA meth	34463	Non-ische	187152	A	G	-0.2109
rs156629	17	7.1E+07	DNA meth	34463	Non-ische	187152	C	T	-0.179
rs167045	11	7.7E+07	DNA meth	34463	Non-ische	187152	G	A	0.2007
rs199005	7	4.5E+07	DNA meth	34463	Non-ische	187152	A	G	0.2573
rs230098	10	1E+08	DNA meth	34463	Non-ische	187152	G	A	0.1833
rs264726	4	1.1E+08	DNA meth	34463	Non-ische	187152	G	A	0.176

rs375938	17	1.5E+07	DNA meth	34463	Non-ische	187152	A	G	-0.1904
rs382995	17	3378876	DNA meth	34463	Non-ische	187152	T	C	-0.3796
rs467051	2	3.6E+07	DNA meth	34463	Non-ische	187152	A	C	0.1927
rs533852	3	1E+08	DNA meth	34463	Non-ische	187152	T	C	-0.1741
rs575118	22	2.3E+07	DNA meth	34463	Non-ische	187152	T	C	-0.3915
rs644067	3	1.5E+08	DNA meth	34463	Non-ische	187152	A	G	-0.2251
rs653111	2	1.7E+07	DNA meth	34463	Non-ische	187152	C	T	0.2542
rs678553	1	2.4E+08	DNA meth	34463	Non-ische	187152	C	T	-0.3265
rs716433	15	7.8E+07	DNA meth	34463	Non-ische	187152	C	T	-0.2031
rs727655	2	1828908	DNA meth	34463	Non-ische	187152	T	C	-0.9593
rs730280	11	1.2E+08	DNA meth	34463	Non-ische	187152	A	G	-0.4329
rs752223	1	6E+07	DNA meth	34463	Non-ische	187152	A	G	-0.5602
rs767763	4	1.9E+08	DNA meth	34463	Non-ische	187152	C	T	-0.2755
rs791391	10	3.1E+07	DNA meth	34463	Non-ische	187152	T	C	0.1874
rs798208	1	1.6E+08	DNA meth	34463	Non-ische	187152	C	T	0.9005
rs927121	20	5.1E+07	DNA meth	34463	Non-ische	187152	C	T	-0.2149
rs938625	6	1.5E+08	DNA meth	34463	Non-ische	187152	T	G	-0.372
rs940978	9	9.7E+07	DNA meth	34463	Non-ische	187152	A	G	0.2691
rs987068	3	7.2E+07	DNA meth	34463	Non-ische	187152	T	C	0.2221
rs100527	10	3.8E+07	DNA meth	34449	Pulmonar	218792	C	A	-0.3006
rs107862	10	9.8E+07	DNA meth	34449	Pulmonar	218792	G	A	0.36
rs111901	10	1E+08	DNA meth	34449	Pulmonar	218792	T	C	0.147
rs114606	3	3.2E+07	DNA meth	34449	Pulmonar	218792	T	C	0.3554
rs115161	12	1.1E+07	DNA meth	34449	Pulmonar	218792	A	G	0.1773
rs120033	14	3.5E+07	DNA meth	34449	Pulmonar	218792	C	T	-0.179
rs121968	6	1.5E+08	DNA meth	34449	Pulmonar	218792	T	C	0.4614
rs122718	11	7.8E+07	DNA meth	34449	Pulmonar	218792	A	G	0.364
rs124177	11	6.6E+07	DNA meth	34449	Pulmonar	218792	C	T	0.2093
rs148436	2	1.1E+08	DNA meth	34449	Pulmonar	218792	T	C	0.49
rs159885	4	1E+08	DNA meth	34449	Pulmonar	218792	G	A	-0.1858
rs169584	17	9445045	DNA meth	34449	Pulmonar	218792	C	T	0.1533
rs184920	18	4.2E+07	DNA meth	34449	Pulmonar	218792	T	G	-0.1647
rs198496	14	9.6E+07	DNA meth	34449	Pulmonar	218792	G	A	-0.2047
rs201420	1	2.1E+08	DNA meth	34449	Pulmonar	218792	A	G	0.1696
rs219511	2	1.5E+08	DNA meth	34449	Pulmonar	218792	C	T	0.1614
rs264725	4	1.1E+08	DNA meth	34449	Pulmonar	218792	A	G	-0.1436
rs290805	7	1.3E+08	DNA meth	34449	Pulmonar	218792	T	C	-0.2268
rs309395	6	3.1E+07	DNA meth	34449	Pulmonar	218792	C	T	0.2428
rs414198	11	1E+08	DNA meth	34449	Pulmonar	218792	C	T	-0.8116
rs467990	3	1.6E+08	DNA meth	34449	Pulmonar	218792	T	C	0.1538
rs477047	13	2.4E+07	DNA meth	34449	Pulmonar	218792	T	C	0.159
rs483859	10	5E+07	DNA meth	34449	Pulmonar	218792	T	C	-0.2576

rs6054829	20	7203816	DNA meth	34449	Pulmonar	218792	G	A	0.276
rs623855	6	1.4E+07	DNA meth	34449	Pulmonar	218792	T	C	0.279
rs644067(	3	1.5E+08	DNA meth	34449	Pulmonar	218792	A	G	-0.1915
rs701777	8	1340507	DNA meth	34449	Pulmonar	218792	T	C	0.146
rs770552(	5	1285974	DNA meth	34449	Pulmonar	218792	A	C	0.1811
rs772994	7	4.9E+07	DNA meth	34449	Pulmonar	218792	C	T	0.7584
rs787122(	12	5.5E+07	DNA meth	34449	Pulmonar	218792	A	C	-0.231
rs796594	12	2.5E+07	DNA meth	34449	Pulmonar	218792	C	T	0.2095
rs800530(	14	2.1E+07	DNA meth	34449	Pulmonar	218792	A	C	0.322
rs801958	10	1.3E+08	DNA meth	34449	Pulmonar	218792	C	T	-0.2695
rs878175	6	1.4E+08	DNA meth	34449	Pulmonar	218792	C	T	0.1903
rs981384(	3	1.9E+08	DNA meth	34449	Pulmonar	218792	C	T	-0.4373
rs108494	12	6493351	DNA meth	34463	Pulmonar	218792	G	A	-0.2346
rs111901	10	1E+08	DNA meth	34463	Pulmonar	218792	A	C	0.2484
rs112106	3	3.8E+07	DNA meth	34463	Pulmonar	218792	A	G	1.483
rs112452(	2	1.3E+08	DNA meth	34463	Pulmonar	218792	A	G	0.7517
rs112533	10	759559	DNA meth	34463	Pulmonar	218792	T	C	0.2846
rs114234	6	1.8E+07	DNA meth	34463	Pulmonar	218792	C	T	-0.8235
rs116853	17	5.5E+07	DNA meth	34463	Pulmonar	218792	A	G	0.5517
rs128188	12	8.2E+07	DNA meth	34463	Pulmonar	218792	T	C	0.2944
rs148794	1	8895237	DNA meth	34463	Pulmonar	218792	A	G	-0.2109
rs156629	17	7.1E+07	DNA meth	34463	Pulmonar	218792	C	T	-0.179
rs167045	11	7.7E+07	DNA meth	34463	Pulmonar	218792	G	A	0.2007
rs199005	7	4.5E+07	DNA meth	34463	Pulmonar	218792	A	G	0.2573
rs230098	10	1E+08	DNA meth	34463	Pulmonar	218792	G	A	0.1833
rs264726(	4	1.1E+08	DNA meth	34463	Pulmonar	218792	G	A	0.176
rs375938	17	1.5E+07	DNA meth	34463	Pulmonar	218792	A	G	-0.1904
rs382995	17	3378876	DNA meth	34463	Pulmonar	218792	T	C	-0.3796
rs467051	2	3.6E+07	DNA meth	34463	Pulmonar	218792	A	C	0.1927
rs533852	3	1E+08	DNA meth	34463	Pulmonar	218792	T	C	-0.1741
rs575118(	22	2.3E+07	DNA meth	34463	Pulmonar	218792	T	C	-0.3915
rs644067(	3	1.5E+08	DNA meth	34463	Pulmonar	218792	A	G	-0.2251
rs653111	2	1.7E+07	DNA meth	34463	Pulmonar	218792	C	T	0.2542
rs678553	1	2.4E+08	DNA meth	34463	Pulmonar	218792	C	T	-0.3265
rs716433	15	7.8E+07	DNA meth	34463	Pulmonar	218792	C	T	-0.2031
rs727655(	2	1828908	DNA meth	34463	Pulmonar	218792	T	C	-0.9593
rs730280	11	1.2E+08	DNA meth	34463	Pulmonar	218792	A	G	-0.4329
rs752223	1	6E+07	DNA meth	34463	Pulmonar	218792	A	G	-0.5602
rs767763	4	1.9E+08	DNA meth	34463	Pulmonar	218792	C	T	-0.2755
rs791391	10	3.1E+07	DNA meth	34463	Pulmonar	218792	T	C	0.1874
rs798208	1	1.6E+08	DNA meth	34463	Pulmonar	218792	C	T	0.9005
rs927121	20	5.1E+07	DNA meth	34463	Pulmonar	218792	C	T	-0.2149

rs938625(	6	1.5E+08	DNA meth	34463	Pulmonar	218792	T	G	-0.372
rs940978:	9	9.7E+07	DNA meth	34463	Pulmonar	218792	A	G	0.2691
rs987068:	3	7.2E+07	DNA meth	34463	Pulmonar	218792	T	C	0.2221
rs115353(	5	7.2E+07	DNA meth	34470	Pulmonar	218792	T	C	0.0064
rs121888(	5	4.5E+07	DNA meth	34470	Pulmonar	218792	C	A	-0.0101
rs131336	4	3.8E+07	DNA meth	34470	Pulmonar	218792	C	T	-0.004
rs132400:	13	4.3E+07	DNA meth	34470	Pulmonar	218792	T	C	0.004
rs147021:	3	6.7E+07	DNA meth	34470	Pulmonar	218792	T	C	-0.0102
rs218923	4	1.1E+08	DNA meth	34470	Pulmonar	218792	G	T	-0.0037
rs358360(	2	1.1E+08	DNA meth	34470	Pulmonar	218792	G	A	0.0045
rs549280	4	7.5E+07	DNA meth	34470	Pulmonar	218792	A	G	-0.0041
rs559982:	11	7.4E+07	DNA meth	34470	Pulmonar	218792	G	A	0.0078
rs606977	20	5.5E+07	DNA meth	34470	Pulmonar	218792	T	C	0.0035
rs609287:	9	9.1E+07	DNA meth	34470	Pulmonar	218792	C	T	0.0049
rs697937	7	2.9E+07	DNA meth	34470	Pulmonar	218792	A	G	0.0036
rs726831:	8	1.1E+08	DNA meth	34470	Pulmonar	218792	G	A	-0.0056
rs777009!	6	4.1E+07	DNA meth	34470	Pulmonar	218792	A	G	-0.017
rs785728!	2	4.4E+07	DNA meth	34470	Pulmonar	218792	C	T	0.0071
rs807045	17	3.8E+07	DNA meth	34470	Pulmonar	218792	T	C	0.0061
rs115227:	5	1.1E+08	DNA meth	34467	Pulmonar	218792	T	C	-0.5517
rs121167:	1	1E+08	DNA meth	34467	Pulmonar	218792	T	C	-0.266
rs134126	2	1.1E+08	DNA meth	34467	Pulmonar	218792	G	A	-0.3915
rs138988:	5	1.6E+08	DNA meth	34467	Pulmonar	218792	A	G	0.59
rs148545(	6	1.5E+08	DNA meth	34467	Pulmonar	218792	T	C	1.0581
rs169362:	10	8E+07	DNA meth	34467	Pulmonar	218792	C	T	-0.1908
rs170941	10	1E+08	DNA meth	34467	Pulmonar	218792	G	A	0.18
rs172798!	15	8E+07	DNA meth	34467	Pulmonar	218792	A	G	-0.1737
rs180044(	2	3.8E+07	DNA meth	34467	Pulmonar	218792	C	T	0.1834
rs183718!	2	2E+08	DNA meth	34467	Pulmonar	218792	C	T	0.1641
rs201005	3	1.3E+08	DNA meth	34467	Pulmonar	218792	G	A	0.1456
rs381736:	3	1.6E+08	DNA meth	34467	Pulmonar	218792	G	T	-0.2347
rs406532:	17	3.8E+07	DNA meth	34467	Pulmonar	218792	T	C	-0.1703
rs670929(	2	1.6E+08	DNA meth	34467	Pulmonar	218792	T	G	0.151
rs688653:	5	1.5E+08	DNA meth	34467	Pulmonar	218792	T	C	-0.3752
rs726597:	13	1E+08	DNA meth	34467	Pulmonar	218792	T	C	0.2962
rs761833:	11	1.2E+07	DNA meth	34467	Pulmonar	218792	T	C	0.2802
rs773853	19	1.7E+07	DNA meth	34467	Pulmonar	218792	G	A	-0.174
rs774886!	4	1.9E+08	DNA meth	34467	Pulmonar	218792	C	T	0.2283
rs790051	2	2.3E+08	DNA meth	34467	Pulmonar	218792	A	G	-0.1676
rs791923!	10	1.8E+07	DNA meth	34467	Pulmonar	218792	C	T	0.1787
rs887466	6	3.1E+07	DNA meth	34467	Pulmonar	218792	A	G	-0.1928
rs938679(	6	1.1E+08	DNA meth	34467	Pulmonar	218792	T	C	0.1983

rs979067!	4	1.1E+08	DNA meth	34467	Pulmonar	218792	G	A	-0.2246
rs116086.	12	5.3E+07	DNA meth	34448	Pulmonar	218792	T	C	-590.41
rs143673.	8	1E+08	DNA meth	34448	Pulmonar	218792	G	A	-368.48
rs148694.	16	9E+07	DNA meth	34448	Pulmonar	218792	T	C	339.43
rs204672!	12	9.6E+07	DNA meth	34448	Pulmonar	218792	T	C	0.3916
rs249885.	9	1.1E+08	DNA meth	34448	Pulmonar	218792	C	T	0.3822
rs285147.	7	2288916	DNA meth	34448	Pulmonar	218792	C	T	1604.03
rs561738(	22	5.1E+07	DNA meth	34448	Pulmonar	218792	A	G	421.813
rs620671(	16	9E+07	DNA meth	34448	Pulmonar	218792	T	C	443.729
rs727863.	21	4.3E+07	DNA meth	34448	Pulmonar	218792	G	T	-0.9444
rs749216.	1	1.9E+07	DNA meth	34448	Pulmonar	218792	A	G	634.602
rs757809.	4	1.9E+08	DNA meth	34448	Pulmonar	218792	A	G	-355.66
rs761465(	7	1.4E+08	DNA meth	34448	Pulmonar	218792	A	G	-439.48
rs782126(	22	5.1E+07	DNA meth	34448	Pulmonar	218792	T	C	347.26
rs788843!	4	1.4E+08	DNA meth	34448	Pulmonar	218792	T	C	-373.26
rs115353(	5	7.2E+07	DNA meth	34470	Valvular h	218792	T	C	0.0064
rs121888(	5	4.5E+07	DNA meth	34470	Valvular h	218792	C	A	-0.0101
rs131336.	4	3.8E+07	DNA meth	34470	Valvular h	218792	C	T	-0.004
rs132400.	13	4.3E+07	DNA meth	34470	Valvular h	218792	T	C	0.004
rs147021.	3	6.7E+07	DNA meth	34470	Valvular h	218792	T	C	-0.0102
rs218923.	4	1.1E+08	DNA meth	34470	Valvular h	218792	G	T	-0.0037
rs358360(	2	1.1E+08	DNA meth	34470	Valvular h	218792	G	A	0.0045
rs549280	4	7.5E+07	DNA meth	34470	Valvular h	218792	A	G	-0.0041
rs559982.	11	7.4E+07	DNA meth	34470	Valvular h	218792	G	A	0.0078
rs606977.	20	5.5E+07	DNA meth	34470	Valvular h	218792	T	C	0.0035
rs609287.	9	9.1E+07	DNA meth	34470	Valvular h	218792	C	T	0.0049
rs697937.	7	2.9E+07	DNA meth	34470	Valvular h	218792	A	G	0.0036
rs726831.	8	1.1E+08	DNA meth	34470	Valvular h	218792	G	A	-0.0056
rs777009!	6	4.1E+07	DNA meth	34470	Valvular h	218792	A	G	-0.017
rs785728!	2	4.4E+07	DNA meth	34470	Valvular h	218792	C	T	0.0071
rs807045.	17	3.8E+07	DNA meth	34470	Valvular h	218792	T	C	0.0061
rs108494.	12	6493351	DNA meth	34463	Valvular h	218792	G	A	-0.2346
rs111901.	10	1E+08	DNA meth	34463	Valvular h	218792	A	C	0.2484
rs112106!	3	3.8E+07	DNA meth	34463	Valvular h	218792	A	G	1.483
rs112452(	2	1.3E+08	DNA meth	34463	Valvular h	218792	A	G	0.7517
rs112533.	10	759559	DNA meth	34463	Valvular h	218792	T	C	0.2846
rs114234!	6	1.8E+07	DNA meth	34463	Valvular h	218792	C	T	-0.8235
rs116853.	17	5.5E+07	DNA meth	34463	Valvular h	218792	A	G	0.5517
rs128188.	12	8.2E+07	DNA meth	34463	Valvular h	218792	T	C	0.2944
rs148794!	1	8895237	DNA meth	34463	Valvular h	218792	A	G	-0.2109
rs156629!	17	7.1E+07	DNA meth	34463	Valvular h	218792	C	T	-0.179
rs167045!	11	7.7E+07	DNA meth	34463	Valvular h	218792	G	A	0.2007

rs1990053	7	4.5E+07	DNA meth	34463	Valvular h	218792	A	G	0.2573
rs2300984	10	1E+08	DNA meth	34463	Valvular h	218792	G	A	0.1833
rs2647266	4	1.1E+08	DNA meth	34463	Valvular h	218792	G	A	0.176
rs375938	17	1.5E+07	DNA meth	34463	Valvular h	218792	A	G	-0.1904
rs3829957	17	3378876	DNA meth	34463	Valvular h	218792	T	C	-0.3796
rs4670518	2	3.6E+07	DNA meth	34463	Valvular h	218792	A	C	0.1927
rs533852	3	1E+08	DNA meth	34463	Valvular h	218792	T	C	-0.1741
rs5751180	22	2.3E+07	DNA meth	34463	Valvular h	218792	T	C	-0.3915
rs6440670	3	1.5E+08	DNA meth	34463	Valvular h	218792	A	G	-0.2251
rs6531114	2	1.7E+07	DNA meth	34463	Valvular h	218792	C	T	0.2542
rs678553	1	2.4E+08	DNA meth	34463	Valvular h	218792	C	T	-0.3265
rs7164338	15	7.8E+07	DNA meth	34463	Valvular h	218792	C	T	-0.2031
rs7276556	2	1828908	DNA meth	34463	Valvular h	218792	T	C	-0.9593
rs7302807	11	1.2E+08	DNA meth	34463	Valvular h	218792	A	G	-0.4329
rs752223	1	6E+07	DNA meth	34463	Valvular h	218792	A	G	-0.5602
rs7677639	4	1.9E+08	DNA meth	34463	Valvular h	218792	C	T	-0.2755
rs7913917	10	3.1E+07	DNA meth	34463	Valvular h	218792	T	C	0.1874
rs7982088	1	1.6E+08	DNA meth	34463	Valvular h	218792	C	T	0.9005
rs927121	20	5.1E+07	DNA meth	34463	Valvular h	218792	C	T	-0.2149
rs9386256	6	1.5E+08	DNA meth	34463	Valvular h	218792	T	G	-0.372
rs9409787	9	9.7E+07	DNA meth	34463	Valvular h	218792	A	G	0.2691
rs9870681	3	7.2E+07	DNA meth	34463	Valvular h	218792	T	C	0.2221
rs1005277	10	3.8E+07	DNA meth	34449	Valvular h	218792	C	A	-0.3006
rs1078628	10	9.8E+07	DNA meth	34449	Valvular h	218792	G	A	0.36
rs1119013	10	1E+08	DNA meth	34449	Valvular h	218792	T	C	0.147
rs1146061	3	3.2E+07	DNA meth	34449	Valvular h	218792	T	C	0.3554
rs1151617	12	1.1E+07	DNA meth	34449	Valvular h	218792	A	G	0.1773
rs1200332	14	3.5E+07	DNA meth	34449	Valvular h	218792	C	T	-0.179
rs1219681	6	1.5E+08	DNA meth	34449	Valvular h	218792	T	C	0.4614
rs1227180	11	7.8E+07	DNA meth	34449	Valvular h	218792	A	G	0.364
rs1241775	11	6.6E+07	DNA meth	34449	Valvular h	218792	C	T	0.2093
rs1484362	2	1.1E+08	DNA meth	34449	Valvular h	218792	T	C	0.49
rs1598856	4	1E+08	DNA meth	34449	Valvular h	218792	G	A	-0.1858
rs1695845	17	9445045	DNA meth	34449	Valvular h	218792	C	T	0.1533
rs1849209	18	4.2E+07	DNA meth	34449	Valvular h	218792	T	G	-0.1647
rs1984968	14	9.6E+07	DNA meth	34449	Valvular h	218792	G	A	-0.2047
rs2014202	1	2.1E+08	DNA meth	34449	Valvular h	218792	A	G	0.1696
rs2195114	2	1.5E+08	DNA meth	34449	Valvular h	218792	C	T	0.1614
rs2647254	4	1.1E+08	DNA meth	34449	Valvular h	218792	A	G	-0.1436
rs290805	7	1.3E+08	DNA meth	34449	Valvular h	218792	T	C	-0.2268
rs3093956	6	3.1E+07	DNA meth	34449	Valvular h	218792	C	T	0.2428
rs4141989	11	1E+08	DNA meth	34449	Valvular h	218792	C	T	-0.8116

rs4679900	3	1.6E+08	DNA meth	34449	Valvular h	218792	T	C	0.1538
rs4770473	13	2.4E+07	DNA meth	34449	Valvular h	218792	T	C	0.159
rs4838595	10	5E+07	DNA meth	34449	Valvular h	218792	T	C	-0.2576
rs6054829	20	7203816	DNA meth	34449	Valvular h	218792	G	A	0.276
rs6238555	6	1.4E+07	DNA meth	34449	Valvular h	218792	T	C	0.279
rs6440670	3	1.5E+08	DNA meth	34449	Valvular h	218792	A	G	-0.1915
rs7017774	8	1340507	DNA meth	34449	Valvular h	218792	T	C	0.146
rs7705526	5	1285974	DNA meth	34449	Valvular h	218792	A	C	0.1811
rs7729949	7	4.9E+07	DNA meth	34449	Valvular h	218792	C	T	0.7584
rs7871226	12	5.5E+07	DNA meth	34449	Valvular h	218792	A	C	-0.231
rs7965945	12	2.5E+07	DNA meth	34449	Valvular h	218792	C	T	0.2095
rs8005306	14	2.1E+07	DNA meth	34449	Valvular h	218792	A	C	0.322
rs8019582	10	1.3E+08	DNA meth	34449	Valvular h	218792	C	T	-0.2695
rs878175	6	1.4E+08	DNA meth	34449	Valvular h	218792	C	T	0.1903
rs9813840	3	1.9E+08	DNA meth	34449	Valvular h	218792	C	T	-0.4373
rs1152271	5	1.1E+08	DNA meth	34467	Valvular h	218792	T	C	-0.5517
rs1211671	1	1E+08	DNA meth	34467	Valvular h	218792	T	C	-0.266
rs1341264	2	1.1E+08	DNA meth	34467	Valvular h	218792	G	A	-0.3915
rs1389887	5	1.6E+08	DNA meth	34467	Valvular h	218792	A	G	0.59
rs1485456	6	1.5E+08	DNA meth	34467	Valvular h	218792	T	C	1.0581
rs1693623	10	8E+07	DNA meth	34467	Valvular h	218792	C	T	-0.1908
rs1709414	10	1E+08	DNA meth	34467	Valvular h	218792	G	A	0.18
rs1727988	15	8E+07	DNA meth	34467	Valvular h	218792	A	G	-0.1737
rs1800440	2	3.8E+07	DNA meth	34467	Valvular h	218792	C	T	0.1834
rs1837188	2	2E+08	DNA meth	34467	Valvular h	218792	C	T	0.1641
rs2010054	3	1.3E+08	DNA meth	34467	Valvular h	218792	G	A	0.1456
rs3817367	3	1.6E+08	DNA meth	34467	Valvular h	218792	G	T	-0.2347
rs4065321	17	3.8E+07	DNA meth	34467	Valvular h	218792	T	C	-0.1703
rs6709296	2	1.6E+08	DNA meth	34467	Valvular h	218792	T	G	0.151
rs6886532	5	1.5E+08	DNA meth	34467	Valvular h	218792	T	C	-0.3752
rs7265977	13	1E+08	DNA meth	34467	Valvular h	218792	T	C	0.2962
rs7618337	11	1.2E+07	DNA meth	34467	Valvular h	218792	T	C	0.2802
rs773853	19	1.7E+07	DNA meth	34467	Valvular h	218792	G	A	-0.174
rs7748865	4	1.9E+08	DNA meth	34467	Valvular h	218792	C	T	0.2283
rs790051	2	2.3E+08	DNA meth	34467	Valvular h	218792	A	G	-0.1676
rs7919238	10	1.8E+07	DNA meth	34467	Valvular h	218792	C	T	0.1787
rs887466	6	3.1E+07	DNA meth	34467	Valvular h	218792	A	G	-0.1928
rs9386796	6	1.1E+08	DNA meth	34467	Valvular h	218792	T	C	0.1983
rs9790675	4	1.1E+08	DNA meth	34467	Valvular h	218792	G	A	-0.2246
rs1160862	12	5.3E+07	DNA meth	34448	Valvular h	218792	T	C	-590.41
rs1436732	8	1E+08	DNA meth	34448	Valvular h	218792	G	A	-368.48
rs1486942	16	9E+07	DNA meth	34448	Valvular h	218792	T	C	339.43

rs204672	12	9.6E+07	DNA meth	34448	Valvular h	218792	T	C	0.3916
rs249885	9	1.1E+08	DNA meth	34448	Valvular h	218792	C	T	0.3822
rs285147	7	2288916	DNA meth	34448	Valvular h	218792	C	T	1604.03
rs561738	22	5.1E+07	DNA meth	34448	Valvular h	218792	A	G	421.813
rs620671	16	9E+07	DNA meth	34448	Valvular h	218792	T	C	443.729
rs727863	21	4.3E+07	DNA meth	34448	Valvular h	218792	G	T	-0.9444
rs749216	1	1.9E+07	DNA meth	34448	Valvular h	218792	A	G	634.602
rs757809	4	1.9E+08	DNA meth	34448	Valvular h	218792	A	G	-355.66
rs761465	7	1.4E+08	DNA meth	34448	Valvular h	218792	A	G	-439.48
rs782126	22	5.1E+07	DNA meth	34448	Valvular h	218792	T	C	347.26
rs788843	4	1.4E+08	DNA meth	34448	Valvular h	218792	T	C	-373.26
rs100527	10	3.8E+07	DNA meth	34449	Heart fail	977323	C	A	-0.3006
rs107862	10	9.8E+07	DNA meth	34449	Heart fail	977323	G	A	0.36
rs111901	10	1E+08	DNA meth	34449	Heart fail	977323	T	C	0.147
rs114606	3	3.2E+07	DNA meth	34449	Heart fail	977323	T	C	0.3554
rs115161	12	1.1E+07	DNA meth	34449	Heart fail	977323	A	G	0.1773
rs120033	14	3.5E+07	DNA meth	34449	Heart fail	977323	C	T	-0.179
rs121968	6	1.5E+08	DNA meth	34449	Heart fail	977323	T	C	0.4614
rs122718	11	7.8E+07	DNA meth	34449	Heart fail	977323	A	G	0.364
rs124177	11	6.6E+07	DNA meth	34449	Heart fail	977323	C	T	0.2093
rs148436	2	1.1E+08	DNA meth	34449	Heart fail	977323	T	C	0.49
rs159885	4	1E+08	DNA meth	34449	Heart fail	977323	G	A	-0.1858
rs169584	17	9445045	DNA meth	34449	Heart fail	977323	C	T	0.1533
rs184920	18	4.2E+07	DNA meth	34449	Heart fail	977323	T	G	-0.1647
rs198496	14	9.6E+07	DNA meth	34449	Heart fail	977323	G	A	-0.2047
rs201420	1	2.1E+08	DNA meth	34449	Heart fail	977323	A	G	0.1696
rs219511	2	1.5E+08	DNA meth	34449	Heart fail	977323	C	T	0.1614
rs264725	4	1.1E+08	DNA meth	34449	Heart fail	977323	A	G	-0.1436
rs290805	7	1.3E+08	DNA meth	34449	Heart fail	977323	T	C	-0.2268
rs309395	6	3.1E+07	DNA meth	34449	Heart fail	977323	C	T	0.2428
rs414198	11	1E+08	DNA meth	34449	Heart fail	977323	C	T	-0.8116
rs467990	3	1.6E+08	DNA meth	34449	Heart fail	977323	T	C	0.1538
rs477047	13	2.4E+07	DNA meth	34449	Heart fail	977323	T	C	0.159
rs483859	10	5E+07	DNA meth	34449	Heart fail	977323	T	C	-0.2576
rs605482	20	7203816	DNA meth	34449	Heart fail	977323	G	A	0.276
rs623855	6	1.4E+07	DNA meth	34449	Heart fail	977323	T	C	0.279
rs644067	3	1.5E+08	DNA meth	34449	Heart fail	977323	A	G	-0.1915
rs701777	8	1340507	DNA meth	34449	Heart fail	977323	T	C	0.146
rs770552	5	1285974	DNA meth	34449	Heart fail	977323	A	C	0.1811
rs787122	12	5.5E+07	DNA meth	34449	Heart fail	977323	A	C	-0.231
rs796594	12	2.5E+07	DNA meth	34449	Heart fail	977323	C	T	0.2095
rs800530	14	2.1E+07	DNA meth	34449	Heart fail	977323	A	C	0.322

rs8019582	10	1.3E+08	DNA meth	34449	Heart failu	977323	C	T	-0.2695
rs878175	6	1.4E+08	DNA meth	34449	Heart failu	977323	C	T	0.1903
rs9813840	3	1.9E+08	DNA meth	34449	Heart failu	977323	C	T	-0.4373
rs1084944	12	6493351	DNA meth	34463	Heart failu	977323	G	A	-0.2346
rs1119012	10	1E+08	DNA meth	34463	Heart failu	977323	A	C	0.2484
rs1124526	2	1.3E+08	DNA meth	34463	Heart failu	977323	A	G	0.7517
rs1125333	10	759559	DNA meth	34463	Heart failu	977323	T	C	0.2846
rs1142345	6	1.8E+07	DNA meth	34463	Heart failu	977323	C	T	-0.8235
rs1168531	17	5.5E+07	DNA meth	34463	Heart failu	977323	A	G	0.5517
rs1281881	12	8.2E+07	DNA meth	34463	Heart failu	977323	T	C	0.2944
rs1487949	1	8895237	DNA meth	34463	Heart failu	977323	A	G	-0.2109
rs1566295	17	7.1E+07	DNA meth	34463	Heart failu	977323	C	T	-0.179
rs1670455	11	7.7E+07	DNA meth	34463	Heart failu	977323	G	A	0.2007
rs1990053	7	4.5E+07	DNA meth	34463	Heart failu	977323	A	G	0.2573
rs2300984	10	1E+08	DNA meth	34463	Heart failu	977323	G	A	0.1833
rs2647266	4	1.1E+08	DNA meth	34463	Heart failu	977323	G	A	0.176
rs375938	17	1.5E+07	DNA meth	34463	Heart failu	977323	A	G	-0.1904
rs3829951	17	3378876	DNA meth	34463	Heart failu	977323	T	C	-0.3796
rs4670518	2	3.6E+07	DNA meth	34463	Heart failu	977323	A	C	0.1927
rs533852	3	1E+08	DNA meth	34463	Heart failu	977323	T	C	-0.1741
rs5751180	22	2.3E+07	DNA meth	34463	Heart failu	977323	T	C	-0.3915
rs6440670	3	1.5E+08	DNA meth	34463	Heart failu	977323	A	G	-0.2251
rs6531114	2	1.7E+07	DNA meth	34463	Heart failu	977323	C	T	0.2542
rs678553	1	2.4E+08	DNA meth	34463	Heart failu	977323	C	T	-0.3265
rs7164338	15	7.8E+07	DNA meth	34463	Heart failu	977323	C	T	-0.2031
rs7276556	2	1828908	DNA meth	34463	Heart failu	977323	T	C	-0.9593
rs7302801	11	1.2E+08	DNA meth	34463	Heart failu	977323	A	G	-0.4329
rs752223	1	6E+07	DNA meth	34463	Heart failu	977323	A	G	-0.5602
rs7677639	4	1.9E+08	DNA meth	34463	Heart failu	977323	C	T	-0.2755
rs7913911	10	3.1E+07	DNA meth	34463	Heart failu	977323	T	C	0.1874
rs7982088	1	1.6E+08	DNA meth	34463	Heart failu	977323	C	T	0.9005
rs927121	20	5.1E+07	DNA meth	34463	Heart failu	977323	C	T	-0.2149
rs9386256	6	1.5E+08	DNA meth	34463	Heart failu	977323	T	G	-0.372
rs9409781	9	9.7E+07	DNA meth	34463	Heart failu	977323	A	G	0.2691
rs9870681	3	7.2E+07	DNA meth	34463	Heart failu	977323	T	C	0.2221
rs1152271	5	1.1E+08	DNA meth	34467	Heart failu	977323	T	C	-0.5517
rs1211671	1	1E+08	DNA meth	34467	Heart failu	977323	T	C	-0.266
rs1341264	2	1.1E+08	DNA meth	34467	Heart failu	977323	G	A	-0.3915
rs1389881	5	1.6E+08	DNA meth	34467	Heart failu	977323	A	G	0.59
rs1693623	10	8E+07	DNA meth	34467	Heart failu	977323	C	T	-0.1908
rs1709414	10	1E+08	DNA meth	34467	Heart failu	977323	G	A	0.18
rs1727988	15	8E+07	DNA meth	34467	Heart failu	977323	A	G	-0.1737

rs180044(	2	3.8E+07	DNA meth	34467	Heart failu	977323	C	T	0.1834
rs183718ε	2	2E+08	DNA meth	34467	Heart failu	977323	C	T	0.1641
rs201005₄	3	1.3E+08	DNA meth	34467	Heart failu	977323	G	A	0.1456
rs381736:	3	1.6E+08	DNA meth	34467	Heart failu	977323	G	T	-0.2347
rs406532:	17	3.8E+07	DNA meth	34467	Heart failu	977323	T	C	-0.1703
rs670929(	2	1.6E+08	DNA meth	34467	Heart failu	977323	T	G	0.151
rs688653:	5	1.5E+08	DNA meth	34467	Heart failu	977323	T	C	-0.3752
rs726597:	13	1E+08	DNA meth	34467	Heart failu	977323	T	C	0.2962
rs761833:	11	1.2E+07	DNA meth	34467	Heart failu	977323	T	C	0.2802
rs773853	19	1.7E+07	DNA meth	34467	Heart failu	977323	G	A	-0.174
rs774886!	4	1.9E+08	DNA meth	34467	Heart failu	977323	C	T	0.2283
rs790051	2	2.3E+08	DNA meth	34467	Heart failu	977323	A	G	-0.1676
rs791923ε	10	1.8E+07	DNA meth	34467	Heart failu	977323	C	T	0.1787
rs887466	6	3.1E+07	DNA meth	34467	Heart failu	977323	A	G	-0.1928
rs938679(	6	1.1E+08	DNA meth	34467	Heart failu	977323	T	C	0.1983
rs979067!	4	1.1E+08	DNA meth	34467	Heart failu	977323	G	A	-0.2246
rs115353(	5	7.2E+07	DNA meth	34470	Heart failu	977323	T	C	0.0064
rs121888(	5	4.5E+07	DNA meth	34470	Heart failu	977323	C	A	-0.0101
rs131336₄	4	3.8E+07	DNA meth	34470	Heart failu	977323	C	T	-0.004
rs132400:	13	4.3E+07	DNA meth	34470	Heart failu	977323	T	C	0.004
rs147021:	3	6.7E+07	DNA meth	34470	Heart failu	977323	T	C	-0.0102
rs218923₄	4	1.1E+08	DNA meth	34470	Heart failu	977323	G	T	-0.0037
rs358360(	2	1.1E+08	DNA meth	34470	Heart failu	977323	G	A	0.0045
rs549280	4	7.5E+07	DNA meth	34470	Heart failu	977323	A	G	-0.0041
rs559982:	11	7.4E+07	DNA meth	34470	Heart failu	977323	G	A	0.0078
rs606977₄	20	5.5E+07	DNA meth	34470	Heart failu	977323	T	C	0.0035
rs609287:	9	9.1E+07	DNA meth	34470	Heart failu	977323	C	T	0.0049
rs697937₄	7	2.9E+07	DNA meth	34470	Heart failu	977323	A	G	0.0036
rs726831:	8	1.1E+08	DNA meth	34470	Heart failu	977323	G	A	-0.0056
rs775585:	6	3.1E+07	DNA meth	34470	Heart failu	977323	G	A	0.0036
rs777009!	6	4.1E+07	DNA meth	34470	Heart failu	977323	A	G	-0.017
rs785728ε	2	4.4E+07	DNA meth	34470	Heart failu	977323	C	T	0.0071
rs807045₄	17	3.8E+07	DNA meth	34470	Heart failu	977323	T	C	0.0061
rs116086:	12	5.3E+07	DNA meth	34448	Heart failu	977323	T	C	-590.41
rs143673:	8	1E+08	DNA meth	34448	Heart failu	977323	G	A	-368.48
rs148694:	16	9E+07	DNA meth	34448	Heart failu	977323	T	C	339.43
rs204672!	12	9.6E+07	DNA meth	34448	Heart failu	977323	T	C	0.3916
rs249885:	9	1.1E+08	DNA meth	34448	Heart failu	977323	C	T	0.3822
rs285147₄	7	2288916	DNA meth	34448	Heart failu	977323	C	T	1604.03
rs561738(	22	5.1E+07	DNA meth	34448	Heart failu	977323	A	G	421.813
rs620671(	16	9E+07	DNA meth	34448	Heart failu	977323	T	C	443.729
rs727863:	21	4.3E+07	DNA meth	34448	Heart failu	977323	G	T	-0.9444

rs749216	1	1.9E+07	DNA meth	34448	Heart failu	977323	A	G	634.602
rs757809	4	1.9E+08	DNA meth	34448	Heart failu	977323	A	G	-355.66
rs761465	7	1.4E+08	DNA meth	34448	Heart failu	977323	A	G	-439.48
rs782126	22	5.1E+07	DNA meth	34448	Heart failu	977323	T	C	347.26
rs788843	4	1.4E+08	DNA meth	34448	Heart failu	977323	T	C	-373.26
rs100527	10	3.8E+07	DNA meth	34449	Atrial fibri	1030836	C	A	-0.3006
rs107862	10	9.8E+07	DNA meth	34449	Atrial fibri	1030836	G	A	0.36
rs111901	10	1E+08	DNA meth	34449	Atrial fibri	1030836	T	C	0.147
rs114606	3	3.2E+07	DNA meth	34449	Atrial fibri	1030836	T	C	0.3554
rs115161	12	1.1E+07	DNA meth	34449	Atrial fibri	1030836	A	G	0.1773
rs120033	14	3.5E+07	DNA meth	34449	Atrial fibri	1030836	C	T	-0.179
rs121968	6	1.5E+08	DNA meth	34449	Atrial fibri	1030836	T	C	0.4614
rs122718	11	7.8E+07	DNA meth	34449	Atrial fibri	1030836	A	G	0.364
rs124177	11	6.6E+07	DNA meth	34449	Atrial fibri	1030836	C	T	0.2093
rs148436	2	1.1E+08	DNA meth	34449	Atrial fibri	1030836	T	C	0.49
rs159885	4	1E+08	DNA meth	34449	Atrial fibri	1030836	G	A	-0.1858
rs169584	17	9445045	DNA meth	34449	Atrial fibri	1030836	C	T	0.1533
rs184920	18	4.2E+07	DNA meth	34449	Atrial fibri	1030836	T	G	-0.1647
rs198496	14	9.6E+07	DNA meth	34449	Atrial fibri	1030836	G	A	-0.2047
rs201420	1	2.1E+08	DNA meth	34449	Atrial fibri	1030836	A	G	0.1696
rs219511	2	1.5E+08	DNA meth	34449	Atrial fibri	1030836	C	T	0.1614
rs264725	4	1.1E+08	DNA meth	34449	Atrial fibri	1030836	A	G	-0.1436
rs309395	6	3.1E+07	DNA meth	34449	Atrial fibri	1030836	C	T	0.2428
rs414198	11	1E+08	DNA meth	34449	Atrial fibri	1030836	C	T	-0.8116
rs467990	3	1.6E+08	DNA meth	34449	Atrial fibri	1030836	T	C	0.1538
rs477047	13	2.4E+07	DNA meth	34449	Atrial fibri	1030836	T	C	0.159
rs483859	10	5E+07	DNA meth	34449	Atrial fibri	1030836	T	C	-0.2576
rs605482	20	7203816	DNA meth	34449	Atrial fibri	1030836	G	A	0.276
rs623855	6	1.4E+07	DNA meth	34449	Atrial fibri	1030836	T	C	0.279
rs644067	3	1.5E+08	DNA meth	34449	Atrial fibri	1030836	A	G	-0.1915
rs701777	8	1340507	DNA meth	34449	Atrial fibri	1030836	T	C	0.146
rs770552	5	1285974	DNA meth	34449	Atrial fibri	1030836	A	C	0.1811
rs772994	7	4.9E+07	DNA meth	34449	Atrial fibri	1030836	C	T	0.7584
rs787122	12	5.5E+07	DNA meth	34449	Atrial fibri	1030836	A	C	-0.231
rs796594	12	2.5E+07	DNA meth	34449	Atrial fibri	1030836	C	T	0.2095
rs800530	14	2.1E+07	DNA meth	34449	Atrial fibri	1030836	A	C	0.322
rs801958	10	1.3E+08	DNA meth	34449	Atrial fibri	1030836	C	T	-0.2695
rs878175	6	1.4E+08	DNA meth	34449	Atrial fibri	1030836	C	T	0.1903
rs981384	3	1.9E+08	DNA meth	34449	Atrial fibri	1030836	C	T	-0.4373
rs115353	5	7.2E+07	DNA meth	34470	Atrial fibri	1030836	T	C	0.0064
rs121888	5	4.5E+07	DNA meth	34470	Atrial fibri	1030836	C	A	-0.0101
rs131336	4	3.8E+07	DNA meth	34470	Atrial fibri	1030836	C	T	-0.004

rs132400	13	4.3E+07	DNA meth	34470	Atrial fibri	1030836	T	C	0.004
rs147021	3	6.7E+07	DNA meth	34470	Atrial fibri	1030836	T	C	-0.0102
rs218923	4	1.1E+08	DNA meth	34470	Atrial fibri	1030836	G	T	-0.0037
rs358360	2	1.1E+08	DNA meth	34470	Atrial fibri	1030836	G	A	0.0045
rs549280	4	7.5E+07	DNA meth	34470	Atrial fibri	1030836	A	G	-0.0041
rs559982	11	7.4E+07	DNA meth	34470	Atrial fibri	1030836	G	A	0.0078
rs606977	20	5.5E+07	DNA meth	34470	Atrial fibri	1030836	T	C	0.0035
rs609287	9	9.1E+07	DNA meth	34470	Atrial fibri	1030836	C	T	0.0049
rs697937	7	2.9E+07	DNA meth	34470	Atrial fibri	1030836	A	G	0.0036
rs726831	8	1.1E+08	DNA meth	34470	Atrial fibri	1030836	G	A	-0.0056
rs775585	6	3.1E+07	DNA meth	34470	Atrial fibri	1030836	G	A	0.0036
rs777009	6	4.1E+07	DNA meth	34470	Atrial fibri	1030836	A	G	-0.017
rs785728	2	4.4E+07	DNA meth	34470	Atrial fibri	1030836	C	T	0.0071
rs807045	17	3.8E+07	DNA meth	34470	Atrial fibri	1030836	T	C	0.0061
rs115227	5	1.1E+08	DNA meth	34467	Atrial fibri	1030836	T	C	-0.5517
rs121167	1	1E+08	DNA meth	34467	Atrial fibri	1030836	T	C	-0.266
rs134126	2	1.1E+08	DNA meth	34467	Atrial fibri	1030836	G	A	-0.3915
rs138988	5	1.6E+08	DNA meth	34467	Atrial fibri	1030836	A	G	0.59
rs148545	6	1.5E+08	DNA meth	34467	Atrial fibri	1030836	T	C	1.0581
rs169362	10	8E+07	DNA meth	34467	Atrial fibri	1030836	C	T	-0.1908
rs170941	10	1E+08	DNA meth	34467	Atrial fibri	1030836	G	A	0.18
rs172798	15	8E+07	DNA meth	34467	Atrial fibri	1030836	A	G	-0.1737
rs180044	2	3.8E+07	DNA meth	34467	Atrial fibri	1030836	C	T	0.1834
rs183718	2	2E+08	DNA meth	34467	Atrial fibri	1030836	C	T	0.1641
rs201005	3	1.3E+08	DNA meth	34467	Atrial fibri	1030836	G	A	0.1456
rs381736	3	1.6E+08	DNA meth	34467	Atrial fibri	1030836	G	T	-0.2347
rs406532	17	3.8E+07	DNA meth	34467	Atrial fibri	1030836	T	C	-0.1703
rs670929	2	1.6E+08	DNA meth	34467	Atrial fibri	1030836	T	G	0.151
rs688653	5	1.5E+08	DNA meth	34467	Atrial fibri	1030836	T	C	-0.3752
rs726597	13	1E+08	DNA meth	34467	Atrial fibri	1030836	T	C	0.2962
rs761833	11	1.2E+07	DNA meth	34467	Atrial fibri	1030836	T	C	0.2802
rs773853	19	1.7E+07	DNA meth	34467	Atrial fibri	1030836	G	A	-0.174
rs774886	4	1.9E+08	DNA meth	34467	Atrial fibri	1030836	C	T	0.2283
rs790051	2	2.3E+08	DNA meth	34467	Atrial fibri	1030836	A	G	-0.1676
rs791923	10	1.8E+07	DNA meth	34467	Atrial fibri	1030836	C	T	0.1787
rs887466	6	3.1E+07	DNA meth	34467	Atrial fibri	1030836	A	G	-0.1928
rs938679	6	1.1E+08	DNA meth	34467	Atrial fibri	1030836	T	C	0.1983
rs979067	4	1.1E+08	DNA meth	34467	Atrial fibri	1030836	G	A	-0.2246
rs115227	5	1.1E+08	DNA meth	34467	Coronary	184305	T	C	-0.5517
rs121167	1	1E+08	DNA meth	34467	Coronary	184305	T	C	-0.266
rs134126	2	1.1E+08	DNA meth	34467	Coronary	184305	G	A	-0.3915
rs138988	5	1.6E+08	DNA meth	34467	Coronary	184305	A	G	0.59

rs148545	6	1.5E+08	DNA meth	34467	Coronary	184305	T	C	1.0581
rs169362	10	8E+07	DNA meth	34467	Coronary	184305	C	T	-0.1908
rs170941	10	1E+08	DNA meth	34467	Coronary	184305	G	A	0.18
rs172798	15	8E+07	DNA meth	34467	Coronary	184305	A	G	-0.1737
rs180044	2	3.8E+07	DNA meth	34467	Coronary	184305	C	T	0.1834
rs183718	2	2E+08	DNA meth	34467	Coronary	184305	C	T	0.1641
rs201005	3	1.3E+08	DNA meth	34467	Coronary	184305	G	A	0.1456
rs381736	3	1.6E+08	DNA meth	34467	Coronary	184305	G	T	-0.2347
rs406532	17	3.8E+07	DNA meth	34467	Coronary	184305	T	C	-0.1703
rs670929	2	1.6E+08	DNA meth	34467	Coronary	184305	T	G	0.151
rs688653	5	1.5E+08	DNA meth	34467	Coronary	184305	T	C	-0.3752
rs726597	13	1E+08	DNA meth	34467	Coronary	184305	T	C	0.2962
rs761833	11	1.2E+07	DNA meth	34467	Coronary	184305	T	C	0.2802
rs773853	19	1.7E+07	DNA meth	34467	Coronary	184305	G	A	-0.174
rs774886	4	1.9E+08	DNA meth	34467	Coronary	184305	C	T	0.2283
rs790051	2	2.3E+08	DNA meth	34467	Coronary	184305	A	G	-0.1676
rs791923	10	1.8E+07	DNA meth	34467	Coronary	184305	C	T	0.1787
rs938679	6	1.1E+08	DNA meth	34467	Coronary	184305	T	C	0.1983
rs979067	4	1.1E+08	DNA meth	34467	Coronary	184305	G	A	-0.2246
rs115353	5	7.2E+07	DNA meth	34470	Coronary	184305	T	C	0.0064
rs121888	5	4.5E+07	DNA meth	34470	Coronary	184305	C	A	-0.0101
rs131336	4	3.8E+07	DNA meth	34470	Coronary	184305	C	T	-0.004
rs132400	13	4.3E+07	DNA meth	34470	Coronary	184305	T	C	0.004
rs147021	3	6.7E+07	DNA meth	34470	Coronary	184305	T	C	-0.0102
rs218923	4	1.1E+08	DNA meth	34470	Coronary	184305	G	T	-0.0037
rs358360	2	1.1E+08	DNA meth	34470	Coronary	184305	G	A	0.0045
rs549280	4	7.5E+07	DNA meth	34470	Coronary	184305	A	G	-0.0041
rs606977	20	5.5E+07	DNA meth	34470	Coronary	184305	T	C	0.0035
rs609287	9	9.1E+07	DNA meth	34470	Coronary	184305	C	T	0.0049
rs697937	7	2.9E+07	DNA meth	34470	Coronary	184305	A	G	0.0036
rs726831	8	1.1E+08	DNA meth	34470	Coronary	184305	G	A	-0.0056
rs777009	6	4.1E+07	DNA meth	34470	Coronary	184305	A	G	-0.017
rs785728	2	4.4E+07	DNA meth	34470	Coronary	184305	C	T	0.0071
rs807045	17	3.8E+07	DNA meth	34470	Coronary	184305	T	C	0.0061
rs100527	10	3.8E+07	DNA meth	34449	Coronary	184305	C	A	-0.3006
rs107862	10	9.8E+07	DNA meth	34449	Coronary	184305	G	A	0.36
rs111901	10	1E+08	DNA meth	34449	Coronary	184305	T	C	0.147
rs114606	3	3.2E+07	DNA meth	34449	Coronary	184305	T	C	0.3554
rs115161	12	1.1E+07	DNA meth	34449	Coronary	184305	A	G	0.1773
rs120033	14	3.5E+07	DNA meth	34449	Coronary	184305	C	T	-0.179
rs121968	6	1.5E+08	DNA meth	34449	Coronary	184305	T	C	0.4614
rs122718	11	7.8E+07	DNA meth	34449	Coronary	184305	A	G	0.364

rs1241775	11	6.6E+07	DNA meth	34449	Coronary	184305	C	T	0.2093
rs1484362	2	1.1E+08	DNA meth	34449	Coronary	184305	T	C	0.49
rs1598856	4	1E+08	DNA meth	34449	Coronary	184305	G	A	-0.1858
rs1695845	17	9445045	DNA meth	34449	Coronary	184305	C	T	0.1533
rs1849209	18	4.2E+07	DNA meth	34449	Coronary	184305	T	G	-0.1647
rs1984968	14	9.6E+07	DNA meth	34449	Coronary	184305	G	A	-0.2047
rs2014202	1	2.1E+08	DNA meth	34449	Coronary	184305	A	G	0.1696
rs2195114	2	1.5E+08	DNA meth	34449	Coronary	184305	C	T	0.1614
rs2647254	4	1.1E+08	DNA meth	34449	Coronary	184305	A	G	-0.1436
rs290805	7	1.3E+08	DNA meth	34449	Coronary	184305	T	C	-0.2268
rs4141989	11	1E+08	DNA meth	34449	Coronary	184305	C	T	-0.8116
rs4679900	3	1.6E+08	DNA meth	34449	Coronary	184305	T	C	0.1538
rs4770473	13	2.4E+07	DNA meth	34449	Coronary	184305	T	C	0.159
rs4838595	10	5E+07	DNA meth	34449	Coronary	184305	T	C	-0.2576
rs6054829	20	7203816	DNA meth	34449	Coronary	184305	G	A	0.276
rs6238555	6	1.4E+07	DNA meth	34449	Coronary	184305	T	C	0.279
rs6440670	3	1.5E+08	DNA meth	34449	Coronary	184305	A	G	-0.1915
rs7017774	8	1340507	DNA meth	34449	Coronary	184305	T	C	0.146
rs7705526	5	1285974	DNA meth	34449	Coronary	184305	A	C	0.1811
rs7871226	12	5.5E+07	DNA meth	34449	Coronary	184305	A	C	-0.231
rs7965945	12	2.5E+07	DNA meth	34449	Coronary	184305	C	T	0.2095
rs8005306	14	2.1E+07	DNA meth	34449	Coronary	184305	A	C	0.322
rs8019582	10	1.3E+08	DNA meth	34449	Coronary	184305	C	T	-0.2695
rs878175	6	1.4E+08	DNA meth	34449	Coronary	184305	C	T	0.1903
rs9813840	3	1.9E+08	DNA meth	34449	Coronary	184305	C	T	-0.4373
rs1152271	5	1.1E+08	DNA meth	34467	Myocardia	171875	T	C	-0.5517
rs1211671	1	1E+08	DNA meth	34467	Myocardia	171875	T	C	-0.266
rs1341264	2	1.1E+08	DNA meth	34467	Myocardia	171875	G	A	-0.3915
rs1389887	5	1.6E+08	DNA meth	34467	Myocardia	171875	A	G	0.59
rs1485456	6	1.5E+08	DNA meth	34467	Myocardia	171875	T	C	1.0581
rs1693623	10	8E+07	DNA meth	34467	Myocardia	171875	C	T	-0.1908
rs1709414	10	1E+08	DNA meth	34467	Myocardia	171875	G	A	0.18
rs1727988	15	8E+07	DNA meth	34467	Myocardia	171875	A	G	-0.1737
rs1800440	2	3.8E+07	DNA meth	34467	Myocardia	171875	C	T	0.1834
rs1837188	2	2E+08	DNA meth	34467	Myocardia	171875	C	T	0.1641
rs2010054	3	1.3E+08	DNA meth	34467	Myocardia	171875	G	A	0.1456
rs3817367	3	1.6E+08	DNA meth	34467	Myocardia	171875	G	T	-0.2347
rs4065321	17	3.8E+07	DNA meth	34467	Myocardia	171875	T	C	-0.1703
rs6709296	2	1.6E+08	DNA meth	34467	Myocardia	171875	T	G	0.151
rs6886532	5	1.5E+08	DNA meth	34467	Myocardia	171875	T	C	-0.3752
rs7265977	13	1E+08	DNA meth	34467	Myocardia	171875	T	C	0.2962
rs7618337	11	1.2E+07	DNA meth	34467	Myocardia	171875	T	C	0.2802

rs773853	19	1.7E+07	DNA meth	34467	Myocardia	171875	G	A	-0.174
rs7748865	4	1.9E+08	DNA meth	34467	Myocardia	171875	C	T	0.2283
rs790051	2	2.3E+08	DNA meth	34467	Myocardia	171875	A	G	-0.1676
rs7919238	10	1.8E+07	DNA meth	34467	Myocardia	171875	C	T	0.1787
rs9386796	6	1.1E+08	DNA meth	34467	Myocardia	171875	T	C	0.1983
rs9790675	4	1.1E+08	DNA meth	34467	Myocardia	171875	G	A	-0.2246
rs1005277	10	3.8E+07	DNA meth	34449	Myocardia	171875	C	A	-0.3006
rs1078628	10	9.8E+07	DNA meth	34449	Myocardia	171875	G	A	0.36
rs1119013	10	1E+08	DNA meth	34449	Myocardia	171875	T	C	0.147
rs1146061	3	3.2E+07	DNA meth	34449	Myocardia	171875	T	C	0.3554
rs1151617	12	1.1E+07	DNA meth	34449	Myocardia	171875	A	G	0.1773
rs1200332	14	3.5E+07	DNA meth	34449	Myocardia	171875	C	T	-0.179
rs1219681	6	1.5E+08	DNA meth	34449	Myocardia	171875	T	C	0.4614
rs1227180	11	7.8E+07	DNA meth	34449	Myocardia	171875	A	G	0.364
rs1241775	11	6.6E+07	DNA meth	34449	Myocardia	171875	C	T	0.2093
rs1484362	2	1.1E+08	DNA meth	34449	Myocardia	171875	T	C	0.49
rs1598856	4	1E+08	DNA meth	34449	Myocardia	171875	G	A	-0.1858
rs1695845	17	9445045	DNA meth	34449	Myocardia	171875	C	T	0.1533
rs1849209	18	4.2E+07	DNA meth	34449	Myocardia	171875	T	G	-0.1647
rs1984968	14	9.6E+07	DNA meth	34449	Myocardia	171875	G	A	-0.2047
rs2014202	1	2.1E+08	DNA meth	34449	Myocardia	171875	A	G	0.1696
rs2195114	2	1.5E+08	DNA meth	34449	Myocardia	171875	C	T	0.1614
rs2647254	4	1.1E+08	DNA meth	34449	Myocardia	171875	A	G	-0.1436
rs290805	7	1.3E+08	DNA meth	34449	Myocardia	171875	T	C	-0.2268
rs4141989	11	1E+08	DNA meth	34449	Myocardia	171875	C	T	-0.8116
rs4679900	3	1.6E+08	DNA meth	34449	Myocardia	171875	T	C	0.1538
rs4770473	13	2.4E+07	DNA meth	34449	Myocardia	171875	T	C	0.159
rs4838595	10	5E+07	DNA meth	34449	Myocardia	171875	T	C	-0.2576
rs6054829	20	7203816	DNA meth	34449	Myocardia	171875	G	A	0.276
rs6238555	6	1.4E+07	DNA meth	34449	Myocardia	171875	T	C	0.279
rs6440670	3	1.5E+08	DNA meth	34449	Myocardia	171875	A	G	-0.1915
rs7017774	8	1340507	DNA meth	34449	Myocardia	171875	T	C	0.146
rs7705526	5	1285974	DNA meth	34449	Myocardia	171875	A	C	0.1811
rs7871226	12	5.5E+07	DNA meth	34449	Myocardia	171875	A	C	-0.231
rs7965945	12	2.5E+07	DNA meth	34449	Myocardia	171875	C	T	0.2095
rs8005306	14	2.1E+07	DNA meth	34449	Myocardia	171875	A	C	0.322
rs8019582	10	1.3E+08	DNA meth	34449	Myocardia	171875	C	T	-0.2695
rs878175	6	1.4E+08	DNA meth	34449	Myocardia	171875	C	T	0.1903
rs9813840	3	1.9E+08	DNA meth	34449	Myocardia	171875	C	T	-0.4373
rs1153530	5	7.2E+07	DNA meth	34470	Myocardia	171875	T	C	0.0064
rs1218886	5	4.5E+07	DNA meth	34470	Myocardia	171875	C	A	-0.0101
rs1313364	4	3.8E+07	DNA meth	34470	Myocardia	171875	C	T	-0.004

rs1324003	13	4.3E+07	DNA meth	34470	Myocardia	171875	T	C	0.004
rs1470217	3	6.7E+07	DNA meth	34470	Myocardia	171875	T	C	-0.0102
rs2189234	4	1.1E+08	DNA meth	34470	Myocardia	171875	G	T	-0.0037
rs3583606	2	1.1E+08	DNA meth	34470	Myocardia	171875	G	A	0.0045
rs549280	4	7.5E+07	DNA meth	34470	Myocardia	171875	A	G	-0.0041
rs6069774	20	5.5E+07	DNA meth	34470	Myocardia	171875	T	C	0.0035
rs6092873	9	9.1E+07	DNA meth	34470	Myocardia	171875	C	T	0.0049
rs6979374	7	2.9E+07	DNA meth	34470	Myocardia	171875	A	G	0.0036
rs7268313	8	1.1E+08	DNA meth	34470	Myocardia	171875	G	A	-0.0056
rs7770095	6	4.1E+07	DNA meth	34470	Myocardia	171875	A	G	-0.017
rs7857288	2	4.4E+07	DNA meth	34470	Myocardia	171875	C	T	0.0071
rs8070454	17	3.8E+07	DNA meth	34470	Myocardia	171875	T	C	0.0061
rs1084944	12	6493351	DNA meth	34463	Atrial fibri	1030836	G	A	-0.2346
rs1119012	10	1E+08	DNA meth	34463	Atrial fibri	1030836	A	C	0.2484
rs1121065	3	3.8E+07	DNA meth	34463	Atrial fibri	1030836	A	G	1.483
rs1124526	2	1.3E+08	DNA meth	34463	Atrial fibri	1030836	A	G	0.7517
rs1125333	10	759559	DNA meth	34463	Atrial fibri	1030836	T	C	0.2846
rs1142345	6	1.8E+07	DNA meth	34463	Atrial fibri	1030836	C	T	-0.8235
rs1168537	17	5.5E+07	DNA meth	34463	Atrial fibri	1030836	A	G	0.5517
rs1281881	12	8.2E+07	DNA meth	34463	Atrial fibri	1030836	T	C	0.2944
rs1487949	1	8895237	DNA meth	34463	Atrial fibri	1030836	A	G	-0.2109
rs1566295	17	7.1E+07	DNA meth	34463	Atrial fibri	1030836	C	T	-0.179
rs1670455	11	7.7E+07	DNA meth	34463	Atrial fibri	1030836	G	A	0.2007
rs1990053	7	4.5E+07	DNA meth	34463	Atrial fibri	1030836	A	G	0.2573
rs2300984	10	1E+08	DNA meth	34463	Atrial fibri	1030836	G	A	0.1833
rs2647266	4	1.1E+08	DNA meth	34463	Atrial fibri	1030836	G	A	0.176
rs375938	17	1.5E+07	DNA meth	34463	Atrial fibri	1030836	A	G	-0.1904
rs3829957	17	3378876	DNA meth	34463	Atrial fibri	1030836	T	C	-0.3796
rs4670518	2	3.6E+07	DNA meth	34463	Atrial fibri	1030836	A	C	0.1927
rs533852	3	1E+08	DNA meth	34463	Atrial fibri	1030836	T	C	-0.1741
rs6440670	3	1.5E+08	DNA meth	34463	Atrial fibri	1030836	A	G	-0.2251
rs6531114	2	1.7E+07	DNA meth	34463	Atrial fibri	1030836	C	T	0.2542
rs678553	1	2.4E+08	DNA meth	34463	Atrial fibri	1030836	C	T	-0.3265
rs7164338	15	7.8E+07	DNA meth	34463	Atrial fibri	1030836	C	T	-0.2031
rs7276556	2	1828908	DNA meth	34463	Atrial fibri	1030836	T	C	-0.9593
rs7302807	11	1.2E+08	DNA meth	34463	Atrial fibri	1030836	A	G	-0.4329
rs752223	1	6E+07	DNA meth	34463	Atrial fibri	1030836	A	G	-0.5602
rs7677639	4	1.9E+08	DNA meth	34463	Atrial fibri	1030836	C	T	-0.2755
rs7913917	10	3.1E+07	DNA meth	34463	Atrial fibri	1030836	T	C	0.1874
rs7982088	1	1.6E+08	DNA meth	34463	Atrial fibri	1030836	C	T	0.9005
rs927121	20	5.1E+07	DNA meth	34463	Atrial fibri	1030836	C	T	-0.2149
rs9386256	6	1.5E+08	DNA meth	34463	Atrial fibri	1030836	T	G	-0.372

rs9409787	9	9.7E+07	DNA meth	34463	Atrial fibri	1030836	A	G	0.2691
rs9870681	3	7.2E+07	DNA meth	34463	Atrial fibri	1030836	T	C	0.2221
rs1160862	12	5.3E+07	DNA meth	34448	Atrial fibri	1030836	T	C	-590.41
rs1436732	8	1E+08	DNA meth	34448	Atrial fibri	1030836	G	A	-368.48
rs1486942	16	9E+07	DNA meth	34448	Atrial fibri	1030836	T	C	339.43
rs2046729	12	9.6E+07	DNA meth	34448	Atrial fibri	1030836	T	C	0.3916
rs2498857	9	1.1E+08	DNA meth	34448	Atrial fibri	1030836	C	T	0.3822
rs2851474	7	2288916	DNA meth	34448	Atrial fibri	1030836	C	T	1604.03
rs5617386	22	5.1E+07	DNA meth	34448	Atrial fibri	1030836	A	G	421.813
rs6206710	16	9E+07	DNA meth	34448	Atrial fibri	1030836	T	C	443.729
rs7278637	21	4.3E+07	DNA meth	34448	Atrial fibri	1030836	G	T	-0.9444
rs7492167	1	1.9E+07	DNA meth	34448	Atrial fibri	1030836	A	G	634.602
rs7578093	4	1.9E+08	DNA meth	34448	Atrial fibri	1030836	A	G	-355.66
rs7614650	7	1.4E+08	DNA meth	34448	Atrial fibri	1030836	A	G	-439.48
rs7821260	22	5.1E+07	DNA meth	34448	Atrial fibri	1030836	T	C	347.26
rs7888438	4	1.4E+08	DNA meth	34448	Atrial fibri	1030836	T	C	-373.26
rs1084944	12	6493351	DNA meth	34463	Coronary	184305	G	A	-0.2346
rs1119012	10	1E+08	DNA meth	34463	Coronary	184305	A	C	0.2484
rs1121065	3	3.8E+07	DNA meth	34463	Coronary	184305	A	G	1.483
rs1124526	2	1.3E+08	DNA meth	34463	Coronary	184305	A	G	0.7517
rs1125333	10	759559	DNA meth	34463	Coronary	184305	T	C	0.2846
rs1142345	6	1.8E+07	DNA meth	34463	Coronary	184305	C	T	-0.8235
rs1168537	17	5.5E+07	DNA meth	34463	Coronary	184305	A	G	0.5517
rs1281881	12	8.2E+07	DNA meth	34463	Coronary	184305	T	C	0.2944
rs1487949	1	8895237	DNA meth	34463	Coronary	184305	A	G	-0.2109
rs1566295	17	7.1E+07	DNA meth	34463	Coronary	184305	C	T	-0.179
rs1670455	11	7.7E+07	DNA meth	34463	Coronary	184305	G	A	0.2007
rs1990053	7	4.5E+07	DNA meth	34463	Coronary	184305	A	G	0.2573
rs2300984	10	1E+08	DNA meth	34463	Coronary	184305	G	A	0.1833
rs2647266	4	1.1E+08	DNA meth	34463	Coronary	184305	G	A	0.176
rs375938	17	1.5E+07	DNA meth	34463	Coronary	184305	A	G	-0.1904
rs3829957	17	3378876	DNA meth	34463	Coronary	184305	T	C	-0.3796
rs4670518	2	3.6E+07	DNA meth	34463	Coronary	184305	A	C	0.1927
rs533852	3	1E+08	DNA meth	34463	Coronary	184305	T	C	-0.1741
rs5751180	22	2.3E+07	DNA meth	34463	Coronary	184305	T	C	-0.3915
rs6440670	3	1.5E+08	DNA meth	34463	Coronary	184305	A	G	-0.2251
rs6531114	2	1.7E+07	DNA meth	34463	Coronary	184305	C	T	0.2542
rs678553	1	2.4E+08	DNA meth	34463	Coronary	184305	C	T	-0.3265
rs7164338	15	7.8E+07	DNA meth	34463	Coronary	184305	C	T	-0.2031
rs7276556	2	1828908	DNA meth	34463	Coronary	184305	T	C	-0.9593
rs7302807	11	1.2E+08	DNA meth	34463	Coronary	184305	A	G	-0.4329
rs752223	1	6E+07	DNA meth	34463	Coronary	184305	A	G	-0.5602

rs7677639	4	1.9E+08	DNA meth	34463	Coronary	184305	C	T	-0.2755
rs7913917	10	3.1E+07	DNA meth	34463	Coronary	184305	T	C	0.1874
rs7982088	1	1.6E+08	DNA meth	34463	Coronary	184305	C	T	0.9005
rs927121	20	5.1E+07	DNA meth	34463	Coronary	184305	C	T	-0.2149
rs9386256	6	1.5E+08	DNA meth	34463	Coronary	184305	T	G	-0.372
rs9409787	9	9.7E+07	DNA meth	34463	Coronary	184305	A	G	0.2691
rs9870681	3	7.2E+07	DNA meth	34463	Coronary	184305	T	C	0.2221
rs1160862	12	5.3E+07	DNA meth	34448	Coronary	184305	T	C	-590.41
rs1436732	8	1E+08	DNA meth	34448	Coronary	184305	G	A	-368.48
rs1486942	16	9E+07	DNA meth	34448	Coronary	184305	T	C	339.43
rs2046729	12	9.6E+07	DNA meth	34448	Coronary	184305	T	C	0.3916
rs2498857	9	1.1E+08	DNA meth	34448	Coronary	184305	C	T	0.3822
rs2851474	7	2288916	DNA meth	34448	Coronary	184305	C	T	1604.03
rs5617386	22	5.1E+07	DNA meth	34448	Coronary	184305	A	G	421.813
rs6206710	16	9E+07	DNA meth	34448	Coronary	184305	T	C	443.729
rs7278637	21	4.3E+07	DNA meth	34448	Coronary	184305	G	T	-0.9444
rs7492167	1	1.9E+07	DNA meth	34448	Coronary	184305	A	G	634.602
rs7578093	4	1.9E+08	DNA meth	34448	Coronary	184305	A	G	-355.66
rs7614650	7	1.4E+08	DNA meth	34448	Coronary	184305	A	G	-439.48
rs7821260	22	5.1E+07	DNA meth	34448	Coronary	184305	T	C	347.26
rs7888438	4	1.4E+08	DNA meth	34448	Coronary	184305	T	C	-373.26
rs1160862	12	5.3E+07	DNA meth	34448	Myocardia	171875	T	C	-590.41
rs1436732	8	1E+08	DNA meth	34448	Myocardia	171875	G	A	-368.48
rs1486942	16	9E+07	DNA meth	34448	Myocardia	171875	T	C	339.43
rs2046729	12	9.6E+07	DNA meth	34448	Myocardia	171875	T	C	0.3916
rs2498857	9	1.1E+08	DNA meth	34448	Myocardia	171875	C	T	0.3822
rs2851474	7	2288916	DNA meth	34448	Myocardia	171875	C	T	1604.03
rs5617386	22	5.1E+07	DNA meth	34448	Myocardia	171875	A	G	421.813
rs7278637	21	4.3E+07	DNA meth	34448	Myocardia	171875	G	T	-0.9444
rs7492167	1	1.9E+07	DNA meth	34448	Myocardia	171875	A	G	634.602
rs7578093	4	1.9E+08	DNA meth	34448	Myocardia	171875	A	G	-355.66
rs7614650	7	1.4E+08	DNA meth	34448	Myocardia	171875	A	G	-439.48
rs7821260	22	5.1E+07	DNA meth	34448	Myocardia	171875	T	C	347.26
rs7888438	4	1.4E+08	DNA meth	34448	Myocardia	171875	T	C	-373.26
rs1084944	12	6493351	DNA meth	34463	Myocardia	171875	G	A	-0.2346
rs1119012	10	1E+08	DNA meth	34463	Myocardia	171875	A	C	0.2484
rs1121065	3	3.8E+07	DNA meth	34463	Myocardia	171875	A	G	1.483
rs1124526	2	1.3E+08	DNA meth	34463	Myocardia	171875	A	G	0.7517
rs1125333	10	759559	DNA meth	34463	Myocardia	171875	T	C	0.2846
rs1142345	6	1.8E+07	DNA meth	34463	Myocardia	171875	C	T	-0.8235
rs1168537	17	5.5E+07	DNA meth	34463	Myocardia	171875	A	G	0.5517
rs1281881	12	8.2E+07	DNA meth	34463	Myocardia	171875	T	C	0.2944

rs1487949	1	8895237	DNA meth	34463	Myocardia	171875	A	G	-0.2109
rs156629	17	7.1E+07	DNA meth	34463	Myocardia	171875	C	T	-0.179
rs167045	11	7.7E+07	DNA meth	34463	Myocardia	171875	G	A	0.2007
rs199005	7	4.5E+07	DNA meth	34463	Myocardia	171875	A	G	0.2573
rs230098	10	1E+08	DNA meth	34463	Myocardia	171875	G	A	0.1833
rs264726	4	1.1E+08	DNA meth	34463	Myocardia	171875	G	A	0.176
rs375938	17	1.5E+07	DNA meth	34463	Myocardia	171875	A	G	-0.1904
rs382995	17	3378876	DNA meth	34463	Myocardia	171875	T	C	-0.3796
rs467051	2	3.6E+07	DNA meth	34463	Myocardia	171875	A	C	0.1927
rs533852	3	1E+08	DNA meth	34463	Myocardia	171875	T	C	-0.1741
rs575118	22	2.3E+07	DNA meth	34463	Myocardia	171875	T	C	-0.3915
rs644067	3	1.5E+08	DNA meth	34463	Myocardia	171875	A	G	-0.2251
rs653111	2	1.7E+07	DNA meth	34463	Myocardia	171875	C	T	0.2542
rs678553	1	2.4E+08	DNA meth	34463	Myocardia	171875	C	T	-0.3265
rs716433	15	7.8E+07	DNA meth	34463	Myocardia	171875	C	T	-0.2031
rs730280	11	1.2E+08	DNA meth	34463	Myocardia	171875	A	G	-0.4329
rs752223	1	6E+07	DNA meth	34463	Myocardia	171875	A	G	-0.5602
rs767763	4	1.9E+08	DNA meth	34463	Myocardia	171875	C	T	-0.2755
rs791391	10	3.1E+07	DNA meth	34463	Myocardia	171875	T	C	0.1874
rs798208	1	1.6E+08	DNA meth	34463	Myocardia	171875	C	T	0.9005
rs927121	20	5.1E+07	DNA meth	34463	Myocardia	171875	C	T	-0.2149
rs938625	6	1.5E+08	DNA meth	34463	Myocardia	171875	T	G	-0.372
rs940978	9	9.7E+07	DNA meth	34463	Myocardia	171875	A	G	0.2691
rs987068	3	7.2E+07	DNA meth	34463	Myocardia	171875	T	C	0.2221

EAF	SE	P-value	Proxy-SNI	R2	F
0.0931	0.0014	1.93E-06	-	0.00061	20.90
0.0345	0.0022	4.61E-06	-	0.00061	21.08
0.6782	8.00E-04	1.17E-07	-	0.00072	25.00
0.7253	8.00E-04	5.08E-07	-	0.00072	25.00
0.0366	0.0021	1.29E-06	-	0.00068	23.59
0.6225	7.00E-04	2.43E-07	-	0.00081	27.94
0.1576	0.001	4.00E-06	-	0.00059	20.25
0.6313	7.00E-04	3.59E-08	-	0.00099	34.30
0.0583	0.0017	4.06E-06	-	0.00061	21.05
0.416	7.00E-04	1.70E-06	-	0.00072	25.00
0.1345	0.0011	2.69E-06	-	0.00058	19.84
0.4057	7.00E-04	1.03E-06	-	0.00077	26.45
0.1136	0.0012	1.56E-06	-	0.00063	21.78
0.0151	0.0036	3.02E-06	-	0.00065	22.30
0.0683	0.0015	1.13E-06	rs111884	0.00065	22.40
0.3898	7.00E-04	2.46E-17	-	0.0022	75.93
0.7415	0.048	1.05E-06	-	0.00069	23.89
0.3786	0.0397	3.83E-10	rs108833	0.00113	39.15
0.0092	0.3094	1.64E-06	-	0.00067	22.97
0.0291	0.1557	1.37E-06	-	0.00068	23.31
0.184	0.0494	8.48E-09	-	0.00096	33.19
0.0546	0.0871	3.35E-21	-	0.00259	89.39
0.0425	0.0976	1.59E-08	-	0.00093	31.95
0.126	0.0588	5.57E-07	-	0.00073	25.07
0.2656	0.0436	1.34E-06	-	0.00068	23.40
0.4398	0.0384	3.22E-06	-	0.00063	21.73
0.3065	0.0423	2.11E-06	-	0.00065	22.51
0.4231	0.0384	2.05E-11	-	0.0013	44.89
0.4243	0.0381	1.54E-06	-	0.00067	23.14
0.4078	0.0378	3.28E-06	-	0.00063	21.68
0.4179	0.0388	9.22E-07	-	0.0007	24.08
0.1974	0.0482	3.51E-15	-	0.0018	62.02
0.2734	0.0422	4.92E-06	-	0.0006	20.85
0.4967	0.0375	3.51E-06	-	0.00063	21.55
0.8677	0.0764	3.04E-07	-	0.00076	26.26
0.7966	0.0472	1.90E-06	-	0.00066	22.74
0.7367	0.0424	2.07E-09	-	0.00104	35.94
0.3108	0.0412	2.27E-15	-	0.00182	62.80
0.2726	0.0435	3.07E-06	-	0.00063	21.80
0.0237	0.2048	2.80E-06	-	0.00064	21.94
0.0786	0.0768	1.74E-08	-	0.00092	31.77

0.0818	0.072	7.20E-15	-	0.00175	60.53
0.1455	0.0571	1.42E-06	-	0.00068	23.28
0.5625	0.0387	1.29E-06	-	0.00068	23.45
0.0205	0.1882	1.71E-06	-	0.00066	22.89
0.7573	0.0462	3.21E-06	rs602178(	0.00063	21.64
0.0857	0.0723	2.65E-07	-	0.00077	26.47
0.1267	0.0586	4.46E-06	-	0.00061	21.09
0.2457	0.0431	2.64E-07	-	0.00077	26.55
0.7018	0.033	8.92E-20	-	0.0024	82.97
0.7876	0.0362	2.61E-23	-	0.00286	98.89
0.3869	0.0305	1.44E-06	-	0.00067	23.23
0.0538	0.0677	1.54E-07	-	0.0008	27.56
0.2354	0.0362	9.98E-07	-	0.0007	23.99
0.7939	0.0371	1.44E-06	-	0.00068	23.28
0.0385	0.0963	1.64E-06	-	0.00067	22.96
0.0552	0.0765	1.95E-06	-	0.00066	22.64
0.4539	0.0304	6.21E-12	-	0.00137	47.40
0.0334	0.0952	2.64E-07	-	0.00077	26.49
0.5508	0.0296	3.63E-10	-	0.00114	39.40
0.3678	0.0314	1.09E-06	-	0.00069	23.83
0.7591	0.0347	2.07E-06	-	0.00065	22.53
0.1549	0.0414	7.44E-07	-	0.00071	24.45
0.3246	0.0315	7.23E-08	-	0.00084	28.99
0.6655	0.031	1.94E-07	-	0.00079	27.11
0.3504	0.0312	4.03E-06	-	0.00061	21.18
0.1915	0.0401	1.51E-08	-	0.00093	31.99
0.1858	0.0396	8.39E-10	-	0.00109	37.59
0.0132	0.1714	2.19E-06	-	0.00065	22.42
0.5494	0.0304	4.31E-07	-	0.00074	25.59
0.4888	0.0306	2.00E-07	-	0.00078	27.00
0.1217	0.0453	1.29E-08	-	0.00094	32.33
0.0779	0.0601	4.43E-06	-	0.00061	21.09
0.0718	0.0597	2.94E-06	-	0.00063	21.84
0.7966	0.0367	1.79E-07	-	0.00079	27.23
0.506	0.0303	1.43E-06	-	0.00067	23.22
0.3428	0.0338	8.52E-08	-	0.00083	28.71
0.0164	0.1641	3.79E-06	-	0.00062	21.36
0.1122	0.0491	2.60E-06	-	0.00064	22.13
0.1543	0.0424	7.92E-07	-	0.00071	24.41
0.0539	0.0684	2.51E-06	-	0.00064	22.16
0.0811	0.0562	1.59E-06	-	0.00067	22.99
0.1895	0.0385	7.49E-07	-	0.00071	24.43

0.04	0.0859	3.58E-07	-	0.00075	25.91
0.0381	58.1786	3.38E-24	-	0.00298	102.98
0.0294	78.3081	2.53E-06	-	0.00064	22.14
0.0322	63.7358	1.01E-07	-	0.00082	28.36
0.7352	0.0792	7.54E-07	-	0.00071	24.45
0.2731	0.0831	4.29E-06	-	0.00061	21.15
0.0111	125.806	3.12E-37	-	0.0047	162.55
0.0388	55.1122	1.95E-14	-	0.0017	58.58
0.0289	83.642	1.13E-07	-	0.00082	28.14
0.0417	0.2061	4.60E-06	-	0.00061	21.00
0.0182	85.1356	9.06E-14	-	0.00161	55.56
0.0358	67.4644	1.35E-07	-	0.00081	27.79
0.0212	82.8799	1.14E-07	-	0.00082	28.12
0.0259	76.0531	4.97E-06	-	0.0006	20.85
0.0286	71.6693	1.91E-07	-	0.00079	27.12
0.0265	0.1181	2.97E-06	-	0.00063	21.82
0.0976	0.0557	1.76E-06	-	0.00066	22.80
0.0375	0.0831	2.48E-06	rs134106	0.00064	22.19
0.0246	0.12	8.88E-07	-	0.0007	24.17
0.0118	0.2214	1.75E-06	-	0.00066	22.84
0.169	0.0399	1.71E-06	-	0.00066	22.87
0.2933	0.0323	2.55E-08	-	0.0009	31.05
0.2408	0.0348	5.87E-07	-	0.00072	24.91
0.1885	0.037	6.97E-07	-	0.00071	24.57
0.3541	0.032	2.96E-07	-	0.00076	26.30
0.6028	0.0309	2.51E-06	-	0.00064	22.20
0.1021	0.0498	2.46E-06	-	0.00064	22.21
0.5333	0.0296	8.78E-09	-	0.00096	33.10
0.4237	0.0302	5.57E-07	-	0.00072	25.00
0.0537	0.0806	3.19E-06	-	0.00063	21.67
0.0708	0.0631	2.68E-06	-	0.00064	22.03
0.0741	0.0598	2.78E-06	-	0.00064	21.95
0.6514	0.0359	1.22E-06	-	0.00068	23.49
0.1088	0.0497	4.46E-06	-	0.00061	21.10
0.2169	0.0355	2.34E-06	-	0.00065	22.29
0.2017	0.0386	3.75E-06	-	0.00062	21.43
0.3815	0.031	5.09E-10	-	0.00112	38.68
0.4597	0.0294	1.64E-11	-	0.00132	45.49
0.1647	0.0418	7.52E-08	rs979041	0.00084	28.87
0.7018	0.033	8.92E-20	-	0.0024	82.97
0.7876	0.0362	2.61E-23	-	0.00286	98.89
0.3869	0.0305	1.44E-06	-	0.00067	23.23

0.0538	0.0677	1.54E-07	-	0.0008	27.56
0.2354	0.0362	9.98E-07	-	0.0007	23.99
0.7939	0.0371	1.44E-06	-	0.00068	23.28
0.0385	0.0963	1.64E-06	-	0.00067	22.96
0.0552	0.0765	1.95E-06	-	0.00066	22.64
0.4539	0.0304	6.21E-12	-	0.00137	47.40
0.0334	0.0952	2.64E-07	-	0.00077	26.49
0.5508	0.0296	3.63E-10	-	0.00114	39.40
0.3678	0.0314	1.09E-06	-	0.00069	23.83
0.7591	0.0347	2.07E-06	-	0.00065	22.53
0.1549	0.0414	7.44E-07	-	0.00071	24.45
0.3246	0.0315	7.23E-08	-	0.00084	28.99
0.6655	0.031	1.94E-07	-	0.00079	27.11
0.3504	0.0312	4.03E-06	-	0.00061	21.18
0.1915	0.0401	1.51E-08	-	0.00093	31.99
0.1858	0.0396	8.39E-10	-	0.00109	37.59
0.0132	0.1714	2.19E-06	-	0.00065	22.42
0.5494	0.0304	4.31E-07	-	0.00074	25.59
0.4888	0.0306	2.00E-07	-	0.00078	27.00
0.1217	0.0453	1.29E-08	-	0.00094	32.33
0.0779	0.0601	4.43E-06	-	0.00061	21.09
0.0718	0.0597	2.94E-06	-	0.00063	21.84
0.7966	0.0367	1.79E-07	-	0.00079	27.23
0.506	0.0303	1.43E-06	-	0.00067	23.22
0.3428	0.0338	8.52E-08	-	0.00083	28.71
0.0164	0.1641	3.79E-06	-	0.00062	21.36
0.1122	0.0491	2.60E-06	-	0.00064	22.13
0.1543	0.0424	7.92E-07	-	0.00071	24.41
0.0539	0.0684	2.51E-06	-	0.00064	22.16
0.0811	0.0562	1.59E-06	-	0.00067	22.99
0.1895	0.0385	7.49E-07	-	0.00071	24.43
0.04	0.0859	3.58E-07	-	0.00075	25.91
0.0381	58.1786	3.38E-24	-	0.00298	102.98
0.0294	78.3081	2.53E-06	-	0.00064	22.14
0.0322	63.7358	1.01E-07	-	0.00082	28.36
0.7352	0.0792	7.54E-07	-	0.00071	24.45
0.2731	0.0831	4.29E-06	-	0.00061	21.15
0.0111	125.806	3.12E-37	-	0.0047	162.55
0.0388	55.1122	1.95E-14	-	0.0017	58.58
0.0289	83.642	1.13E-07	-	0.00082	28.14
0.0417	0.2061	4.60E-06	-	0.00061	21.00
0.0182	85.1356	9.06E-14	-	0.00161	55.56

0.0358	67.4644	1.35E-07	-	0.00081	27.79
0.0212	82.8799	1.14E-07	-	0.00082	28.12
0.0259	76.0531	4.97E-06	-	0.0006	20.85
0.0286	71.6693	1.91E-07	-	0.00079	27.12
0.7415	0.048	1.05E-06	-	0.00069	23.89
0.3786	0.0397	3.83E-10	rs108833	0.00113	39.15
0.0092	0.3094	1.64E-06	-	0.00067	22.97
0.0291	0.1557	1.37E-06	-	0.00068	23.31
0.184	0.0494	8.48E-09	-	0.00096	33.19
0.0546	0.0871	3.35E-21	-	0.00259	89.39
0.0425	0.0976	1.59E-08	-	0.00093	31.95
0.126	0.0588	5.57E-07	-	0.00073	25.07
0.2656	0.0436	1.34E-06	-	0.00068	23.40
0.4398	0.0384	3.22E-06	-	0.00063	21.73
0.3065	0.0423	2.11E-06	-	0.00065	22.51
0.4231	0.0384	2.05E-11	-	0.0013	44.89
0.4243	0.0381	1.54E-06	-	0.00067	23.14
0.4078	0.0378	3.28E-06	-	0.00063	21.68
0.4179	0.0388	9.22E-07	-	0.0007	24.08
0.1974	0.0482	3.51E-15	-	0.0018	62.02
0.2734	0.0422	4.92E-06	-	0.0006	20.85
0.4967	0.0375	3.51E-06	-	0.00063	21.55
0.8677	0.0764	3.04E-07	-	0.00076	26.26
0.7966	0.0472	1.90E-06	-	0.00066	22.74
0.7367	0.0424	2.07E-09	-	0.00104	35.94
0.3108	0.0412	2.27E-15	-	0.00182	62.80
0.2726	0.0435	3.07E-06	-	0.00063	21.80
0.0237	0.2048	2.80E-06	-	0.00064	21.94
0.0786	0.0768	1.74E-08	-	0.00092	31.77
0.0818	0.072	7.20E-15	-	0.00175	60.53
0.1455	0.0571	1.42E-06	-	0.00068	23.28
0.5625	0.0387	1.29E-06	-	0.00068	23.45
0.0205	0.1882	1.71E-06	-	0.00066	22.89
0.7573	0.0462	3.21E-06	rs602178	0.00063	21.64
0.0857	0.0723	2.65E-07	-	0.00077	26.47
0.1267	0.0586	4.46E-06	-	0.00061	21.09
0.2457	0.0431	2.64E-07	-	0.00077	26.55
0.0265	0.1181	2.97E-06	-	0.00063	21.82
0.0976	0.0557	1.76E-06	-	0.00066	22.80
0.0375	0.0831	2.48E-06	rs134106	0.00064	22.19
0.0246	0.12	8.88E-07	-	0.0007	24.17
0.0118	0.2214	1.75E-06	-	0.00066	22.84

0.169	0.0399	1.71E-06	-	0.00066	22.87
0.2933	0.0323	2.55E-08	-	0.0009	31.05
0.2408	0.0348	5.87E-07	-	0.00072	24.91
0.1885	0.037	6.97E-07	-	0.00071	24.57
0.3541	0.032	2.96E-07	-	0.00076	26.30
0.6028	0.0309	2.51E-06	-	0.00064	22.20
0.1021	0.0498	2.46E-06	-	0.00064	22.21
0.5333	0.0296	8.78E-09	-	0.00096	33.10
0.4237	0.0302	5.57E-07	-	0.00072	25.00
0.0537	0.0806	3.19E-06	-	0.00063	21.67
0.0708	0.0631	2.68E-06	-	0.00064	22.03
0.0741	0.0598	2.78E-06	-	0.00064	21.95
0.6514	0.0359	1.22E-06	-	0.00068	23.49
0.1088	0.0497	4.46E-06	-	0.00061	21.10
0.2169	0.0355	2.34E-06	-	0.00065	22.29
0.2017	0.0386	3.75E-06	-	0.00062	21.43
0.3815	0.031	5.09E-10	-	0.00112	38.68
0.4597	0.0294	1.64E-11	-	0.00132	45.49
0.1647	0.0418	7.52E-08	rs979041	0.00084	28.87
0.0931	0.0014	1.93E-06	-	0.00061	20.90
0.0345	0.0022	4.61E-06	-	0.00061	21.08
0.6782	8.00E-04	1.17E-07	-	0.00072	25.00
0.7253	8.00E-04	5.08E-07	-	0.00072	25.00
0.0366	0.0021	1.29E-06	-	0.00068	23.59
0.6225	7.00E-04	2.43E-07	-	0.00081	27.94
0.1576	0.001	4.00E-06	-	0.00059	20.25
0.6313	7.00E-04	3.59E-08	-	0.00099	34.30
0.0583	0.0017	4.06E-06	-	0.00061	21.05
0.416	7.00E-04	1.70E-06	-	0.00072	25.00
0.1345	0.0011	2.69E-06	-	0.00058	19.84
0.4057	7.00E-04	1.03E-06	-	0.00077	26.45
0.1136	0.0012	1.56E-06	-	0.00063	21.78
0.0151	0.0036	3.02E-06	-	0.00065	22.30
0.0683	0.0015	1.13E-06	rs111884	0.00065	22.40
0.3898	7.00E-04	2.46E-17	-	0.0022	75.93
0.0381	58.1786	3.38E-24	-	0.00298	102.98
0.0294	78.3081	2.53E-06	-	0.00064	22.14
0.0322	63.7358	1.01E-07	-	0.00082	28.36
0.7352	0.0792	7.54E-07	-	0.00071	24.45
0.2731	0.0831	4.29E-06	-	0.00061	21.15
0.0111	125.806	3.12E-37	-	0.0047	162.55
0.0388	55.1122	1.95E-14	-	0.0017	58.58

0.0289	83.642	1.13E-07	-	0.00082	28.14
0.0417	0.2061	4.60E-06	-	0.00061	21.00
0.0182	85.1356	9.06E-14	-	0.00161	55.56
0.0358	67.4644	1.35E-07	-	0.00081	27.79
0.0212	82.8799	1.14E-07	-	0.00082	28.12
0.0259	76.0531	4.97E-06	-	0.0006	20.85
0.0286	71.6693	1.91E-07	-	0.00079	27.12
0.0931	0.0014	1.93E-06	-	0.00061	20.90
0.0345	0.0022	4.61E-06	-	0.00061	21.08
0.6782	8.00E-04	1.17E-07	-	0.00072	25.00
0.7253	8.00E-04	5.08E-07	-	0.00072	25.00
0.0366	0.0021	1.29E-06	-	0.00068	23.59
0.6225	7.00E-04	2.43E-07	-	0.00081	27.94
0.1576	0.001	4.00E-06	-	0.00059	20.25
0.6313	7.00E-04	3.59E-08	-	0.00099	34.30
0.0583	0.0017	4.06E-06	-	0.00061	21.05
0.416	7.00E-04	1.70E-06	-	0.00072	25.00
0.1345	0.0011	2.69E-06	-	0.00058	19.84
0.4057	7.00E-04	1.03E-06	-	0.00077	26.45
0.1136	0.0012	1.56E-06	-	0.00063	21.78
0.0151	0.0036	3.02E-06	-	0.00065	22.30
0.0683	0.0015	1.13E-06	rs111884[illegible]	0.00065	22.40
0.3898	7.00E-04	2.46E-17	-	0.0022	75.93
0.7018	0.033	8.92E-20	-	0.0024	82.97
0.7876	0.0362	2.61E-23	-	0.00286	98.89
0.3869	0.0305	1.44E-06	-	0.00067	23.23
0.0538	0.0677	1.54E-07	-	0.0008	27.56
0.2354	0.0362	9.98E-07	-	0.0007	23.99
0.7939	0.0371	1.44E-06	-	0.00068	23.28
0.0385	0.0963	1.64E-06	-	0.00067	22.96
0.0552	0.0765	1.95E-06	-	0.00066	22.64
0.4539	0.0304	6.21E-12	-	0.00137	47.40
0.0334	0.0952	2.64E-07	-	0.00077	26.49
0.5508	0.0296	3.63E-10	-	0.00114	39.40
0.3678	0.0314	1.09E-06	-	0.00069	23.83
0.7591	0.0347	2.07E-06	-	0.00065	22.53
0.1549	0.0414	7.44E-07	-	0.00071	24.45
0.3246	0.0315	7.23E-08	-	0.00084	28.99
0.6655	0.031	1.94E-07	-	0.00079	27.11
0.3504	0.0312	4.03E-06	-	0.00061	21.18
0.1915	0.0401	1.51E-08	-	0.00093	31.99
0.1858	0.0396	8.39E-10	-	0.00109	37.59

0.0132	0.1714	2.19E-06	-	0.00065	22.42
0.5494	0.0304	4.31E-07	-	0.00074	25.59
0.4888	0.0306	2.00E-07	-	0.00078	27.00
0.1217	0.0453	1.29E-08	-	0.00094	32.33
0.0779	0.0601	4.43E-06	-	0.00061	21.09
0.0718	0.0597	2.94E-06	-	0.00063	21.84
0.7966	0.0367	1.79E-07	-	0.00079	27.23
0.506	0.0303	1.43E-06	-	0.00067	23.22
0.3428	0.0338	8.52E-08	-	0.00083	28.71
0.0164	0.1641	3.79E-06	-	0.00062	21.36
0.1122	0.0491	2.60E-06	-	0.00064	22.13
0.1543	0.0424	7.92E-07	-	0.00071	24.41
0.0539	0.0684	2.51E-06	-	0.00064	22.16
0.0811	0.0562	1.59E-06	-	0.00067	22.99
0.1895	0.0385	7.49E-07	-	0.00071	24.43
0.04	0.0859	3.58E-07	-	0.00075	25.91
0.7415	0.048	1.05E-06	-	0.00069	23.89
0.3786	0.0397	3.83E-10	rs108833!	0.00113	39.15
0.0092	0.3094	1.64E-06	-	0.00067	22.97
0.0291	0.1557	1.37E-06	-	0.00068	23.31
0.184	0.0494	8.48E-09	-	0.00096	33.19
0.0546	0.0871	3.35E-21	-	0.00259	89.39
0.0425	0.0976	1.59E-08	-	0.00093	31.95
0.126	0.0588	5.57E-07	-	0.00073	25.07
0.2656	0.0436	1.34E-06	-	0.00068	23.40
0.4398	0.0384	3.22E-06	-	0.00063	21.73
0.3065	0.0423	2.11E-06	-	0.00065	22.51
0.4231	0.0384	2.05E-11	-	0.0013	44.89
0.4243	0.0381	1.54E-06	-	0.00067	23.14
0.4078	0.0378	3.28E-06	-	0.00063	21.68
0.4179	0.0388	9.22E-07	-	0.0007	24.08
0.1974	0.0482	3.51E-15	-	0.0018	62.02
0.2734	0.0422	4.92E-06	-	0.0006	20.85
0.4967	0.0375	3.51E-06	-	0.00063	21.55
0.8677	0.0764	3.04E-07	-	0.00076	26.26
0.7966	0.0472	1.90E-06	-	0.00066	22.74
0.7367	0.0424	2.07E-09	-	0.00104	35.94
0.3108	0.0412	2.27E-15	-	0.00182	62.80
0.2726	0.0435	3.07E-06	-	0.00063	21.80
0.0237	0.2048	2.80E-06	-	0.00064	21.94
0.0786	0.0768	1.74E-08	-	0.00092	31.77
0.0818	0.072	7.20E-15	-	0.00175	60.53

0.1455	0.0571	1.42E-06	-	0.00068	23.28
0.5625	0.0387	1.29E-06	-	0.00068	23.45
0.0205	0.1882	1.71E-06	-	0.00066	22.89
0.7573	0.0462	3.21E-06	rs602178[illegible]	0.00063	21.64
0.0857	0.0723	2.65E-07	-	0.00077	26.47
0.1267	0.0586	4.46E-06	-	0.00061	21.09
0.2457	0.0431	2.64E-07	-	0.00077	26.55
0.0265	0.1181	2.97E-06	-	0.00063	21.82
0.0976	0.0557	1.76E-06	-	0.00066	22.80
0.0375	0.0831	2.48E-06	rs134106[illegible]	0.00064	22.19
0.0246	0.12	8.88E-07	-	0.0007	24.17
0.0118	0.2214	1.75E-06	-	0.00066	22.84
0.169	0.0399	1.71E-06	-	0.00066	22.87
0.2933	0.0323	2.55E-08	-	0.0009	31.05
0.2408	0.0348	5.87E-07	-	0.00072	24.91
0.1885	0.037	6.97E-07	-	0.00071	24.57
0.3541	0.032	2.96E-07	-	0.00076	26.30
0.6028	0.0309	2.51E-06	-	0.00064	22.20
0.1021	0.0498	2.46E-06	-	0.00064	22.21
0.5333	0.0296	8.78E-09	-	0.00096	33.10
0.4237	0.0302	5.57E-07	-	0.00072	25.00
0.0537	0.0806	3.19E-06	-	0.00063	21.67
0.0708	0.0631	2.68E-06	-	0.00064	22.03
0.0741	0.0598	2.78E-06	-	0.00064	21.95
0.6514	0.0359	1.22E-06	-	0.00068	23.49
0.1088	0.0497	4.46E-06	-	0.00061	21.10
0.2169	0.0355	2.34E-06	-	0.00065	22.29
0.2017	0.0386	3.75E-06	-	0.00062	21.43
0.3815	0.031	5.09E-10	-	0.00112	38.68
0.4597	0.0294	1.64E-11	-	0.00132	45.49
0.1647	0.0418	7.52E-08	rs979041[illegible]	0.00084	28.87
0.7415	0.048	1.05E-06	-	0.00069	23.89
0.3786	0.0397	3.83E-10	rs108833[illegible]	0.00113	39.15
0.0092	0.3094	1.64E-06	-	0.00067	22.97
0.0291	0.1557	1.37E-06	-	0.00068	23.31
0.184	0.0494	8.48E-09	-	0.00096	33.19
0.0546	0.0871	3.35E-21	-	0.00259	89.39
0.0425	0.0976	1.59E-08	-	0.00093	31.95
0.126	0.0588	5.57E-07	-	0.00073	25.07
0.2656	0.0436	1.34E-06	-	0.00068	23.40
0.4398	0.0384	3.22E-06	-	0.00063	21.73
0.3065	0.0423	2.11E-06	-	0.00065	22.51

0.4231	0.0384	2.05E-11	-	0.0013	44.89
0.4243	0.0381	1.54E-06	-	0.00067	23.14
0.4078	0.0378	3.28E-06	-	0.00063	21.68
0.4179	0.0388	9.22E-07	-	0.0007	24.08
0.1974	0.0482	3.51E-15	-	0.0018	62.02
0.2734	0.0422	4.92E-06	-	0.0006	20.85
0.4967	0.0375	3.51E-06	-	0.00063	21.55
0.8677	0.0764	3.04E-07	-	0.00076	26.26
0.7966	0.0472	1.90E-06	-	0.00066	22.74
0.7367	0.0424	2.07E-09	-	0.00104	35.94
0.3108	0.0412	2.27E-15	-	0.00182	62.80
0.2726	0.0435	3.07E-06	-	0.00063	21.80
0.0237	0.2048	2.80E-06	-	0.00064	21.94
0.0786	0.0768	1.74E-08	-	0.00092	31.77
0.0818	0.072	7.20E-15	-	0.00175	60.53
0.1455	0.0571	1.42E-06	-	0.00068	23.28
0.5625	0.0387	1.29E-06	-	0.00068	23.45
0.0205	0.1882	1.71E-06	-	0.00066	22.89
0.7573	0.0462	3.21E-06	rs602178(	0.00063	21.64
0.0857	0.0723	2.65E-07	-	0.00077	26.47
0.1267	0.0586	4.46E-06	-	0.00061	21.09
0.2457	0.0431	2.64E-07	-	0.00077	26.55
0.0931	0.0014	1.93E-06	-	0.00061	20.90
0.0345	0.0022	4.61E-06	-	0.00061	21.08
0.6782	8.00E-04	1.17E-07	-	0.00072	25.00
0.7253	8.00E-04	5.08E-07	-	0.00072	25.00
0.0366	0.0021	1.29E-06	-	0.00068	23.59
0.6225	7.00E-04	2.43E-07	-	0.00081	27.94
0.1576	0.001	4.00E-06	-	0.00059	20.25
0.6313	7.00E-04	3.59E-08	-	0.00099	34.30
0.0583	0.0017	4.06E-06	-	0.00061	21.05
0.416	7.00E-04	1.70E-06	-	0.00072	25.00
0.1345	0.0011	2.69E-06	-	0.00058	19.84
0.4057	7.00E-04	1.03E-06	-	0.00077	26.45
0.1136	0.0012	1.56E-06	-	0.00063	21.78
0.0151	0.0036	3.02E-06	-	0.00065	22.30
0.0683	0.0015	1.13E-06	rs111884?	0.00065	22.40
0.3898	7.00E-04	2.46E-17	-	0.0022	75.93
0.7018	0.033	8.92E-20	-	0.0024	82.97
0.7876	0.0362	2.61E-23	-	0.00286	98.89
0.3869	0.0305	1.44E-06	-	0.00067	23.23
0.0538	0.0677	1.54E-07	-	0.0008	27.56

0.2354	0.0362	9.98E-07	-	0.0007	23.99
0.7939	0.0371	1.44E-06	-	0.00068	23.28
0.0385	0.0963	1.64E-06	-	0.00067	22.96
0.0552	0.0765	1.95E-06	-	0.00066	22.64
0.4539	0.0304	6.21E-12	-	0.00137	47.40
0.0334	0.0952	2.64E-07	-	0.00077	26.49
0.5508	0.0296	3.63E-10	-	0.00114	39.40
0.3678	0.0314	1.09E-06	-	0.00069	23.83
0.7591	0.0347	2.07E-06	-	0.00065	22.53
0.1549	0.0414	7.44E-07	-	0.00071	24.45
0.3246	0.0315	7.23E-08	-	0.00084	28.99
0.6655	0.031	1.94E-07	-	0.00079	27.11
0.3504	0.0312	4.03E-06	-	0.00061	21.18
0.1915	0.0401	1.51E-08	-	0.00093	31.99
0.1858	0.0396	8.39E-10	-	0.00109	37.59
0.0132	0.1714	2.19E-06	-	0.00065	22.42
0.5494	0.0304	4.31E-07	-	0.00074	25.59
0.4888	0.0306	2.00E-07	-	0.00078	27.00
0.1217	0.0453	1.29E-08	-	0.00094	32.33
0.0779	0.0601	4.43E-06	-	0.00061	21.09
0.0718	0.0597	2.94E-06	-	0.00063	21.84
0.7966	0.0367	1.79E-07	-	0.00079	27.23
0.506	0.0303	1.43E-06	-	0.00067	23.22
0.3428	0.0338	8.52E-08	-	0.00083	28.71
0.0164	0.1641	3.79E-06	-	0.00062	21.36
0.1122	0.0491	2.60E-06	-	0.00064	22.13
0.1543	0.0424	7.92E-07	-	0.00071	24.41
0.0539	0.0684	2.51E-06	-	0.00064	22.16
0.0811	0.0562	1.59E-06	-	0.00067	22.99
0.1895	0.0385	7.49E-07	-	0.00071	24.43
0.04	0.0859	3.58E-07	-	0.00075	25.91
0.0265	0.1181	2.97E-06	-	0.00063	21.82
0.0976	0.0557	1.76E-06	-	0.00066	22.80
0.0375	0.0831	2.48E-06	rs1341068	0.00064	22.19
0.0246	0.12	8.88E-07	-	0.0007	24.17
0.0118	0.2214	1.75E-06	-	0.00066	22.84
0.169	0.0399	1.71E-06	-	0.00066	22.87
0.2933	0.0323	2.55E-08	-	0.0009	31.05
0.2408	0.0348	5.87E-07	-	0.00072	24.91
0.1885	0.037	6.97E-07	-	0.00071	24.57
0.3541	0.032	2.96E-07	-	0.00076	26.30
0.6028	0.0309	2.51E-06	-	0.00064	22.20

0.1021	0.0498	2.46E-06	-	0.00064	22.21
0.5333	0.0296	8.78E-09	-	0.00096	33.10
0.4237	0.0302	5.57E-07	-	0.00072	25.00
0.0537	0.0806	3.19E-06	-	0.00063	21.67
0.0708	0.0631	2.68E-06	-	0.00064	22.03
0.0741	0.0598	2.78E-06	-	0.00064	21.95
0.6514	0.0359	1.22E-06	-	0.00068	23.49
0.1088	0.0497	4.46E-06	-	0.00061	21.10
0.2169	0.0355	2.34E-06	-	0.00065	22.29
0.2017	0.0386	3.75E-06	-	0.00062	21.43
0.3815	0.031	5.09E-10	-	0.00112	38.68
0.4597	0.0294	1.64E-11	-	0.00132	45.49
0.1647	0.0418	7.52E-08	rs979041	0.00084	28.87
0.0381	58.1786	3.38E-24	-	0.00298	102.98
0.0294	78.3081	2.53E-06	-	0.00064	22.14
0.0322	63.7358	1.01E-07	-	0.00082	28.36
0.7352	0.0792	7.54E-07	-	0.00071	24.45
0.2731	0.0831	4.29E-06	-	0.00061	21.15
0.0111	125.806	3.12E-37	-	0.0047	162.55
0.0388	55.1122	1.95E-14	-	0.0017	58.58
0.0289	83.642	1.13E-07	-	0.00082	28.14
0.0417	0.2061	4.60E-06	-	0.00061	21.00
0.0182	85.1356	9.06E-14	-	0.00161	55.56
0.0358	67.4644	1.35E-07	-	0.00081	27.79
0.0212	82.8799	1.14E-07	-	0.00082	28.12
0.0259	76.0531	4.97E-06	-	0.0006	20.85
0.0286	71.6693	1.91E-07	-	0.00079	27.12
0.7018	0.033	8.92E-20	-	0.0024	82.97
0.7876	0.0362	2.61E-23	-	0.00286	98.89
0.3869	0.0305	1.44E-06	-	0.00067	23.23
0.0538	0.0677	1.54E-07	-	0.0008	27.56
0.2354	0.0362	9.98E-07	-	0.0007	23.99
0.7939	0.0371	1.44E-06	-	0.00068	23.28
0.0385	0.0963	1.64E-06	-	0.00067	22.96
0.0552	0.0765	1.95E-06	-	0.00066	22.64
0.4539	0.0304	6.21E-12	-	0.00137	47.40
0.0334	0.0952	2.64E-07	-	0.00077	26.49
0.5508	0.0296	3.63E-10	-	0.00114	39.40
0.3678	0.0314	1.09E-06	-	0.00069	23.83
0.7591	0.0347	2.07E-06	-	0.00065	22.53
0.1549	0.0414	7.44E-07	-	0.00071	24.45
0.3246	0.0315	7.23E-08	-	0.00084	28.99

0.6655	0.031	1.94E-07	-	0.00079	27.11
0.3504	0.0312	4.03E-06	-	0.00061	21.18
0.1915	0.0401	1.51E-08	-	0.00093	31.99
0.1858	0.0396	8.39E-10	-	0.00109	37.59
0.0132	0.1714	2.19E-06	-	0.00065	22.42
0.5494	0.0304	4.31E-07	-	0.00074	25.59
0.4888	0.0306	2.00E-07	-	0.00078	27.00
0.1217	0.0453	1.29E-08	-	0.00094	32.33
0.0779	0.0601	4.43E-06	-	0.00061	21.09
0.0718	0.0597	2.94E-06	-	0.00063	21.84
0.7966	0.0367	1.79E-07	-	0.00079	27.23
0.506	0.0303	1.43E-06	-	0.00067	23.22
0.3428	0.0338	8.52E-08	-	0.00083	28.71
0.0164	0.1641	3.79E-06	-	0.00062	21.36
0.1122	0.0491	2.60E-06	-	0.00064	22.13
0.1543	0.0424	7.92E-07	-	0.00071	24.41
0.0539	0.0684	2.51E-06	-	0.00064	22.16
0.0811	0.0562	1.59E-06	-	0.00067	22.99
0.1895	0.0385	7.49E-07	-	0.00071	24.43
0.04	0.0859	3.58E-07	-	0.00075	25.91
0.7415	0.048	1.05E-06	-	0.00069	23.89
0.3786	0.0397	3.83E-10	rs108833!	0.00113	39.15
0.0092	0.3094	1.64E-06	-	0.00067	22.97
0.0291	0.1557	1.37E-06	-	0.00068	23.31
0.184	0.0494	8.48E-09	-	0.00096	33.19
0.0546	0.0871	3.35E-21	-	0.00259	89.39
0.0425	0.0976	1.59E-08	-	0.00093	31.95
0.126	0.0588	5.57E-07	-	0.00073	25.07
0.2656	0.0436	1.34E-06	-	0.00068	23.40
0.4398	0.0384	3.22E-06	-	0.00063	21.73
0.3065	0.0423	2.11E-06	-	0.00065	22.51
0.4231	0.0384	2.05E-11	-	0.0013	44.89
0.4243	0.0381	1.54E-06	-	0.00067	23.14
0.4078	0.0378	3.28E-06	-	0.00063	21.68
0.4179	0.0388	9.22E-07	-	0.0007	24.08
0.1974	0.0482	3.51E-15	-	0.0018	62.02
0.2734	0.0422	4.92E-06	-	0.0006	20.85
0.4967	0.0375	3.51E-06	-	0.00063	21.55
0.8677	0.0764	3.04E-07	-	0.00076	26.26
0.7966	0.0472	1.90E-06	-	0.00066	22.74
0.7367	0.0424	2.07E-09	-	0.00104	35.94
0.3108	0.0412	2.27E-15	-	0.00182	62.80

0.2726	0.0435	3.07E-06	-	0.00063	21.80
0.0237	0.2048	2.80E-06	-	0.00064	21.94
0.0786	0.0768	1.74E-08	-	0.00092	31.77
0.0818	0.072	7.20E-15	-	0.00175	60.53
0.1455	0.0571	1.42E-06	-	0.00068	23.28
0.5625	0.0387	1.29E-06	-	0.00068	23.45
0.0205	0.1882	1.71E-06	-	0.00066	22.89
0.7573	0.0462	3.21E-06	rs602178	0.00063	21.64
0.0857	0.0723	2.65E-07	-	0.00077	26.47
0.1267	0.0586	4.46E-06	-	0.00061	21.09
0.2457	0.0431	2.64E-07	-	0.00077	26.55
0.0381	58.1786	3.38E-24	-	0.00298	102.98
0.0294	78.3081	2.53E-06	-	0.00064	22.14
0.0322	63.7358	1.01E-07	-	0.00082	28.36
0.7352	0.0792	7.54E-07	-	0.00071	24.45
0.2731	0.0831	4.29E-06	-	0.00061	21.15
0.0111	125.806	3.12E-37	-	0.0047	162.55
0.0388	55.1122	1.95E-14	-	0.0017	58.58
0.0289	83.642	1.13E-07	-	0.00082	28.14
0.0417	0.2061	4.60E-06	-	0.00061	21.00
0.0182	85.1356	9.06E-14	-	0.00161	55.56
0.0358	67.4644	1.35E-07	-	0.00081	27.79
0.0212	82.8799	1.14E-07	-	0.00082	28.12
0.0259	76.0531	4.97E-06	-	0.0006	20.85
0.0286	71.6693	1.91E-07	-	0.00079	27.12
0.0265	0.1181	2.97E-06	-	0.00063	21.82
0.0976	0.0557	1.76E-06	-	0.00066	22.80
0.0375	0.0831	2.48E-06	rs1341068	0.00064	22.19
0.0246	0.12	8.88E-07	-	0.0007	24.17
0.0118	0.2214	1.75E-06	-	0.00066	22.84
0.169	0.0399	1.71E-06	-	0.00066	22.87
0.2933	0.0323	2.55E-08	-	0.0009	31.05
0.2408	0.0348	5.87E-07	-	0.00072	24.91
0.1885	0.037	6.97E-07	-	0.00071	24.57
0.3541	0.032	2.96E-07	-	0.00076	26.30
0.6028	0.0309	2.51E-06	-	0.00064	22.20
0.1021	0.0498	2.46E-06	-	0.00064	22.21
0.5333	0.0296	8.78E-09	-	0.00096	33.10
0.4237	0.0302	5.57E-07	-	0.00072	25.00
0.0537	0.0806	3.19E-06	-	0.00063	21.67
0.0708	0.0631	2.68E-06	-	0.00064	22.03
0.0741	0.0598	2.78E-06	-	0.00064	21.95

0.6514	0.0359	1.22E-06	-	0.00068	23.49
0.1088	0.0497	4.46E-06	-	0.00061	21.10
0.2169	0.0355	2.34E-06	-	0.00065	22.29
0.2017	0.0386	3.75E-06	-	0.00062	21.43
0.3815	0.031	5.09E-10	-	0.00112	38.68
0.4597	0.0294	1.64E-11	-	0.00132	45.49
0.1647	0.0418	7.52E-08	rs979041[illegible]	0.00084	28.87
0.0931	0.0014	1.93E-06	-	0.00061	20.90
0.0345	0.0022	4.61E-06	-	0.00061	21.08
0.6782	8.00E-04	1.17E-07	-	0.00072	25.00
0.7253	8.00E-04	5.08E-07	-	0.00072	25.00
0.0366	0.0021	1.29E-06	-	0.00068	23.59
0.6225	7.00E-04	2.43E-07	-	0.00081	27.94
0.1576	0.001	4.00E-06	-	0.00059	20.25
0.6313	7.00E-04	3.59E-08	-	0.00099	34.30
0.0583	0.0017	4.06E-06	-	0.00061	21.05
0.416	7.00E-04	1.70E-06	-	0.00072	25.00
0.1345	0.0011	2.69E-06	-	0.00058	19.84
0.4057	7.00E-04	1.03E-06	-	0.00077	26.45
0.1136	0.0012	1.56E-06	-	0.00063	21.78
0.0151	0.0036	3.02E-06	-	0.00065	22.30
0.0683	0.0015	1.13E-06	rs111884[illegible]	0.00065	22.40
0.3898	7.00E-04	2.46E-17	-	0.0022	75.93
0.0381	58.1786	3.38E-24	-	0.00298	102.98
0.0294	78.3081	2.53E-06	-	0.00064	22.14
0.0322	63.7358	1.01E-07	-	0.00082	28.36
0.7352	0.0792	7.54E-07	-	0.00071	24.45
0.2731	0.0831	4.29E-06	-	0.00061	21.15
0.0111	125.806	3.12E-37	-	0.0047	162.55
0.0388	55.1122	1.95E-14	-	0.0017	58.58
0.0289	83.642	1.13E-07	-	0.00082	28.14
0.0417	0.2061	4.60E-06	-	0.00061	21.00
0.0182	85.1356	9.06E-14	-	0.00161	55.56
0.0358	67.4644	1.35E-07	-	0.00081	27.79
0.0212	82.8799	1.14E-07	-	0.00082	28.12
0.0259	76.0531	4.97E-06	-	0.0006	20.85
0.0286	71.6693	1.91E-07	-	0.00079	27.12
0.7018	0.033	8.92E-20	-	0.0024	82.97
0.7876	0.0362	2.61E-23	-	0.00286	98.89
0.3869	0.0305	1.44E-06	-	0.00067	23.23
0.0538	0.0677	1.54E-07	-	0.0008	27.56
0.2354	0.0362	9.98E-07	-	0.0007	23.99

0.7939	0.0371	1.44E-06	-	0.00068	23.28
0.0385	0.0963	1.64E-06	-	0.00067	22.96
0.0552	0.0765	1.95E-06	-	0.00066	22.64
0.4539	0.0304	6.21E-12	-	0.00137	47.40
0.0334	0.0952	2.64E-07	-	0.00077	26.49
0.5508	0.0296	3.63E-10	-	0.00114	39.40
0.3678	0.0314	1.09E-06	-	0.00069	23.83
0.7591	0.0347	2.07E-06	-	0.00065	22.53
0.1549	0.0414	7.44E-07	-	0.00071	24.45
0.3246	0.0315	7.23E-08	-	0.00084	28.99
0.6655	0.031	1.94E-07	-	0.00079	27.11
0.3504	0.0312	4.03E-06	-	0.00061	21.18
0.1915	0.0401	1.51E-08	-	0.00093	31.99
0.1858	0.0396	8.39E-10	-	0.00109	37.59
0.0132	0.1714	2.19E-06	-	0.00065	22.42
0.5494	0.0304	4.31E-07	-	0.00074	25.59
0.4888	0.0306	2.00E-07	-	0.00078	27.00
0.1217	0.0453	1.29E-08	-	0.00094	32.33
0.0779	0.0601	4.43E-06	-	0.00061	21.09
0.0718	0.0597	2.94E-06	-	0.00063	21.84
0.7966	0.0367	1.79E-07	-	0.00079	27.23
0.506	0.0303	1.43E-06	-	0.00067	23.22
0.3428	0.0338	8.52E-08	-	0.00083	28.71
0.0164	0.1641	3.79E-06	-	0.00062	21.36
0.1122	0.0491	2.60E-06	-	0.00064	22.13
0.1543	0.0424	7.92E-07	-	0.00071	24.41
0.0539	0.0684	2.51E-06	-	0.00064	22.16
0.0811	0.0562	1.59E-06	-	0.00067	22.99
0.1895	0.0385	7.49E-07	-	0.00071	24.43
0.04	0.0859	3.58E-07	-	0.00075	25.91
0.0931	0.0014	1.93E-06	-	0.00061	20.90
0.0345	0.0022	4.61E-06	-	0.00061	21.08
0.6782	8.00E-04	1.17E-07	-	0.00072	25.00
0.7253	8.00E-04	5.08E-07	-	0.00072	25.00
0.0366	0.0021	1.29E-06	-	0.00068	23.59
0.6225	7.00E-04	2.43E-07	-	0.00081	27.94
0.1576	0.001	4.00E-06	-	0.00059	20.25
0.6313	7.00E-04	3.59E-08	-	0.00099	34.30
0.0583	0.0017	4.06E-06	-	0.00061	21.05
0.416	7.00E-04	1.70E-06	-	0.00072	25.00
0.1345	0.0011	2.69E-06	-	0.00058	19.84
0.4057	7.00E-04	1.03E-06	-	0.00077	26.45

0.1136	0.0012	1.56E-06	-	0.00063	21.78
0.0151	0.0036	3.02E-06	-	0.00065	22.30
0.0683	0.0015	1.13E-06	rs111884	0.00065	22.40
0.3898	7.00E-04	2.46E-17	-	0.0022	75.93
0.0265	0.1181	2.97E-06	-	0.00063	21.82
0.0976	0.0557	1.76E-06	-	0.00066	22.80
0.0375	0.0831	2.48E-06	rs134106	0.00064	22.19
0.0246	0.12	8.88E-07	-	0.0007	24.17
0.0118	0.2214	1.75E-06	-	0.00066	22.84
0.169	0.0399	1.71E-06	-	0.00066	22.87
0.2933	0.0323	2.55E-08	-	0.0009	31.05
0.2408	0.0348	5.87E-07	-	0.00072	24.91
0.1885	0.037	6.97E-07	-	0.00071	24.57
0.3541	0.032	2.96E-07	-	0.00076	26.30
0.6028	0.0309	2.51E-06	-	0.00064	22.20
0.1021	0.0498	2.46E-06	-	0.00064	22.21
0.5333	0.0296	8.78E-09	-	0.00096	33.10
0.4237	0.0302	5.57E-07	-	0.00072	25.00
0.0537	0.0806	3.19E-06	-	0.00063	21.67
0.0708	0.0631	2.68E-06	-	0.00064	22.03
0.0741	0.0598	2.78E-06	-	0.00064	21.95
0.6514	0.0359	1.22E-06	-	0.00068	23.49
0.1088	0.0497	4.46E-06	-	0.00061	21.10
0.2169	0.0355	2.34E-06	-	0.00065	22.29
0.2017	0.0386	3.75E-06	-	0.00062	21.43
0.3815	0.031	5.09E-10	-	0.00112	38.68
0.4597	0.0294	1.64E-11	-	0.00132	45.49
0.1647	0.0418	7.52E-08	rs979041	0.00084	28.87
0.7415	0.048	1.05E-06	-	0.00069	23.89
0.3786	0.0397	3.83E-10	rs108833	0.00113	39.15
0.0092	0.3094	1.64E-06	-	0.00067	22.97
0.0291	0.1557	1.37E-06	-	0.00068	23.31
0.184	0.0494	8.48E-09	-	0.00096	33.19
0.0546	0.0871	3.35E-21	-	0.00259	89.39
0.0425	0.0976	1.59E-08	-	0.00093	31.95
0.126	0.0588	5.57E-07	-	0.00073	25.07
0.2656	0.0436	1.34E-06	-	0.00068	23.40
0.4398	0.0384	3.22E-06	-	0.00063	21.73
0.3065	0.0423	2.11E-06	-	0.00065	22.51
0.4231	0.0384	2.05E-11	-	0.0013	44.89
0.4243	0.0381	1.54E-06	-	0.00067	23.14
0.4078	0.0378	3.28E-06	-	0.00063	21.68

0.4179	0.0388	9.22E-07	-	0.0007	24.08
0.1974	0.0482	3.51E-15	-	0.0018	62.02
0.2734	0.0422	4.92E-06	-	0.0006	20.85
0.4967	0.0375	3.51E-06	-	0.00063	21.55
0.8677	0.0764	3.04E-07	-	0.00076	26.26
0.7966	0.0472	1.90E-06	-	0.00066	22.74
0.7367	0.0424	2.07E-09	-	0.00104	35.94
0.3108	0.0412	2.27E-15	-	0.00182	62.80
0.2726	0.0435	3.07E-06	-	0.00063	21.80
0.0237	0.2048	2.80E-06	-	0.00064	21.94
0.0786	0.0768	1.74E-08	-	0.00092	31.77
0.0818	0.072	7.20E-15	-	0.00175	60.53
0.1455	0.0571	1.42E-06	-	0.00068	23.28
0.5625	0.0387	1.29E-06	-	0.00068	23.45
0.0205	0.1882	1.71E-06	-	0.00066	22.89
0.7573	0.0462	3.21E-06	rs602178(	0.00063	21.64
0.0857	0.0723	2.65E-07	-	0.00077	26.47
0.1267	0.0586	4.46E-06	-	0.00061	21.09
0.2457	0.0431	2.64E-07	-	0.00077	26.55
0.7018	0.033	8.92E-20	-	0.0024	82.97
0.7876	0.0362	2.61E-23	-	0.00286	98.89
0.3869	0.0305	1.44E-06	-	0.00067	23.23
0.0538	0.0677	1.54E-07	-	0.0008	27.56
0.2354	0.0362	9.98E-07	-	0.0007	23.99
0.7939	0.0371	1.44E-06	-	0.00068	23.28
0.0385	0.0963	1.64E-06	-	0.00067	22.96
0.0552	0.0765	1.95E-06	-	0.00066	22.64
0.4539	0.0304	6.21E-12	-	0.00137	47.40
0.0334	0.0952	2.64E-07	-	0.00077	26.49
0.5508	0.0296	3.63E-10	-	0.00114	39.40
0.3678	0.0314	1.09E-06	-	0.00069	23.83
0.7591	0.0347	2.07E-06	-	0.00065	22.53
0.1549	0.0414	7.44E-07	-	0.00071	24.45
0.3246	0.0315	7.23E-08	-	0.00084	28.99
0.6655	0.031	1.94E-07	-	0.00079	27.11
0.3504	0.0312	4.03E-06	-	0.00061	21.18
0.1915	0.0401	1.51E-08	-	0.00093	31.99
0.1858	0.0396	8.39E-10	-	0.00109	37.59
0.0132	0.1714	2.19E-06	-	0.00065	22.42
0.5494	0.0304	4.31E-07	-	0.00074	25.59
0.4888	0.0306	2.00E-07	-	0.00078	27.00
0.1217	0.0453	1.29E-08	-	0.00094	32.33

0.0779	0.0601	4.43E-06	-	0.00061	21.09
0.0718	0.0597	2.94E-06	-	0.00063	21.84
0.7966	0.0367	1.79E-07	-	0.00079	27.23
0.506	0.0303	1.43E-06	-	0.00067	23.22
0.3428	0.0338	8.52E-08	-	0.00083	28.71
0.0164	0.1641	3.79E-06	-	0.00062	21.36
0.1122	0.0491	2.60E-06	-	0.00064	22.13
0.1543	0.0424	7.92E-07	-	0.00071	24.41
0.0539	0.0684	2.51E-06	-	0.00064	22.16
0.0811	0.0562	1.59E-06	-	0.00067	22.99
0.1895	0.0385	7.49E-07	-	0.00071	24.43
0.04	0.0859	3.58E-07	-	0.00075	25.91
0.7415	0.048	1.05E-06	-	0.00069	23.89
0.3786	0.0397	3.83E-10	rs108833	0.00113	39.15
0.0092	0.3094	1.64E-06	-	0.00067	22.97
0.0291	0.1557	1.37E-06	-	0.00068	23.31
0.184	0.0494	8.48E-09	-	0.00096	33.19
0.0546	0.0871	3.35E-21	-	0.00259	89.39
0.0425	0.0976	1.59E-08	-	0.00093	31.95
0.126	0.0588	5.57E-07	-	0.00073	25.07
0.2656	0.0436	1.34E-06	-	0.00068	23.40
0.4398	0.0384	3.22E-06	-	0.00063	21.73
0.3065	0.0423	2.11E-06	-	0.00065	22.51
0.4231	0.0384	2.05E-11	-	0.0013	44.89
0.4243	0.0381	1.54E-06	-	0.00067	23.14
0.4078	0.0378	3.28E-06	-	0.00063	21.68
0.4179	0.0388	9.22E-07	-	0.0007	24.08
0.1974	0.0482	3.51E-15	-	0.0018	62.02
0.2734	0.0422	4.92E-06	-	0.0006	20.85
0.4967	0.0375	3.51E-06	-	0.00063	21.55
0.8677	0.0764	3.04E-07	-	0.00076	26.26
0.7966	0.0472	1.90E-06	-	0.00066	22.74
0.7367	0.0424	2.07E-09	-	0.00104	35.94
0.3108	0.0412	2.27E-15	-	0.00182	62.80
0.2726	0.0435	3.07E-06	-	0.00063	21.80
0.0237	0.2048	2.80E-06	-	0.00064	21.94
0.0786	0.0768	1.74E-08	-	0.00092	31.77
0.0818	0.072	7.20E-15	-	0.00175	60.53
0.1455	0.0571	1.42E-06	-	0.00068	23.28
0.5625	0.0387	1.29E-06	-	0.00068	23.45
0.0205	0.1882	1.71E-06	-	0.00066	22.89
0.7573	0.0462	3.21E-06	rs602178	0.00063	21.64

0.0857	0.0723	2.65E-07	-	0.00077	26.47
0.1267	0.0586	4.46E-06	-	0.00061	21.09
0.2457	0.0431	2.64E-07	-	0.00077	26.55
0.0931	0.0014	1.93E-06	-	0.00061	20.90
0.0345	0.0022	4.61E-06	-	0.00061	21.08
0.6782	8.00E-04	1.17E-07	-	0.00072	25.00
0.7253	8.00E-04	5.08E-07	-	0.00072	25.00
0.0366	0.0021	1.29E-06	-	0.00068	23.59
0.6225	7.00E-04	2.43E-07	-	0.00081	27.94
0.1576	0.001	4.00E-06	-	0.00059	20.25
0.6313	7.00E-04	3.59E-08	-	0.00099	34.30
0.0583	0.0017	4.06E-06	-	0.00061	21.05
0.416	7.00E-04	1.70E-06	-	0.00072	25.00
0.1345	0.0011	2.69E-06	-	0.00058	19.84
0.4057	7.00E-04	1.03E-06	-	0.00077	26.45
0.1136	0.0012	1.56E-06	-	0.00063	21.78
0.0151	0.0036	3.02E-06	-	0.00065	22.30
0.0683	0.0015	1.13E-06	rs111884[illegible]	0.00065	22.40
0.3898	7.00E-04	2.46E-17	-	0.0022	75.93
0.0265	0.1181	2.97E-06	-	0.00063	21.82
0.0976	0.0557	1.76E-06	-	0.00066	22.80
0.0375	0.0831	2.48E-06	rs134106[illegible]	0.00064	22.19
0.0246	0.12	8.88E-07	-	0.0007	24.17
0.0118	0.2214	1.75E-06	-	0.00066	22.84
0.169	0.0399	1.71E-06	-	0.00066	22.87
0.2933	0.0323	2.55E-08	-	0.0009	31.05
0.2408	0.0348	5.87E-07	-	0.00072	24.91
0.1885	0.037	6.97E-07	-	0.00071	24.57
0.3541	0.032	2.96E-07	-	0.00076	26.30
0.6028	0.0309	2.51E-06	-	0.00064	22.20
0.1021	0.0498	2.46E-06	-	0.00064	22.21
0.5333	0.0296	8.78E-09	-	0.00096	33.10
0.4237	0.0302	5.57E-07	-	0.00072	25.00
0.0537	0.0806	3.19E-06	-	0.00063	21.67
0.0708	0.0631	2.68E-06	-	0.00064	22.03
0.0741	0.0598	2.78E-06	-	0.00064	21.95
0.6514	0.0359	1.22E-06	-	0.00068	23.49
0.1088	0.0497	4.46E-06	-	0.00061	21.10
0.2169	0.0355	2.34E-06	-	0.00065	22.29
0.2017	0.0386	3.75E-06	-	0.00062	21.43
0.3815	0.031	5.09E-10	-	0.00112	38.68
0.4597	0.0294	1.64E-11	-	0.00132	45.49

0.1647	0.0418	7.52E-08	rs979041	0.00084	28.87
0.0381	58.1786	3.38E-24	-	0.00298	102.98
0.0294	78.3081	2.53E-06	-	0.00064	22.14
0.0322	63.7358	1.01E-07	-	0.00082	28.36
0.7352	0.0792	7.54E-07	-	0.00071	24.45
0.2731	0.0831	4.29E-06	-	0.00061	21.15
0.0111	125.806	3.12E-37	-	0.0047	162.55
0.0388	55.1122	1.95E-14	-	0.0017	58.58
0.0289	83.642	1.13E-07	-	0.00082	28.14
0.0417	0.2061	4.60E-06	-	0.00061	21.00
0.0182	85.1356	9.06E-14	-	0.00161	55.56
0.0358	67.4644	1.35E-07	-	0.00081	27.79
0.0212	82.8799	1.14E-07	-	0.00082	28.12
0.0259	76.0531	4.97E-06	-	0.0006	20.85
0.0286	71.6693	1.91E-07	-	0.00079	27.12
0.0931	0.0014	1.93E-06	-	0.00061	20.90
0.0345	0.0022	4.61E-06	-	0.00061	21.08
0.6782	8.00E-04	1.17E-07	-	0.00072	25.00
0.7253	8.00E-04	5.08E-07	-	0.00072	25.00
0.0366	0.0021	1.29E-06	-	0.00068	23.59
0.6225	7.00E-04	2.43E-07	-	0.00081	27.94
0.1576	0.001	4.00E-06	-	0.00059	20.25
0.6313	7.00E-04	3.59E-08	-	0.00099	34.30
0.0583	0.0017	4.06E-06	-	0.00061	21.05
0.416	7.00E-04	1.70E-06	-	0.00072	25.00
0.1345	0.0011	2.69E-06	-	0.00058	19.84
0.4057	7.00E-04	1.03E-06	-	0.00077	26.45
0.1136	0.0012	1.56E-06	-	0.00063	21.78
0.0151	0.0036	3.02E-06	-	0.00065	22.30
0.0683	0.0015	1.13E-06	rs111884	0.00065	22.40
0.3898	7.00E-04	2.46E-17	-	0.0022	75.93
0.7415	0.048	1.05E-06	-	0.00069	23.89
0.3786	0.0397	3.83E-10	rs108833	0.00113	39.15
0.0092	0.3094	1.64E-06	-	0.00067	22.97
0.0291	0.1557	1.37E-06	-	0.00068	23.31
0.184	0.0494	8.48E-09	-	0.00096	33.19
0.0546	0.0871	3.35E-21	-	0.00259	89.39
0.0425	0.0976	1.59E-08	-	0.00093	31.95
0.126	0.0588	5.57E-07	-	0.00073	25.07
0.2656	0.0436	1.34E-06	-	0.00068	23.40
0.4398	0.0384	3.22E-06	-	0.00063	21.73
0.3065	0.0423	2.11E-06	-	0.00065	22.51

0.4231	0.0384	2.05E-11	-	0.0013	44.89
0.4243	0.0381	1.54E-06	-	0.00067	23.14
0.4078	0.0378	3.28E-06	-	0.00063	21.68
0.4179	0.0388	9.22E-07	-	0.0007	24.08
0.1974	0.0482	3.51E-15	-	0.0018	62.02
0.2734	0.0422	4.92E-06	-	0.0006	20.85
0.4967	0.0375	3.51E-06	-	0.00063	21.55
0.8677	0.0764	3.04E-07	-	0.00076	26.26
0.7966	0.0472	1.90E-06	-	0.00066	22.74
0.7367	0.0424	2.07E-09	-	0.00104	35.94
0.3108	0.0412	2.27E-15	-	0.00182	62.80
0.2726	0.0435	3.07E-06	-	0.00063	21.80
0.0237	0.2048	2.80E-06	-	0.00064	21.94
0.0786	0.0768	1.74E-08	-	0.00092	31.77
0.0818	0.072	7.20E-15	-	0.00175	60.53
0.1455	0.0571	1.42E-06	-	0.00068	23.28
0.5625	0.0387	1.29E-06	-	0.00068	23.45
0.0205	0.1882	1.71E-06	-	0.00066	22.89
0.7573	0.0462	3.21E-06	rs602178(	0.00063	21.64
0.0857	0.0723	2.65E-07	-	0.00077	26.47
0.1267	0.0586	4.46E-06	-	0.00061	21.09
0.2457	0.0431	2.64E-07	-	0.00077	26.55
0.7018	0.033	8.92E-20	-	0.0024	82.97
0.7876	0.0362	2.61E-23	-	0.00286	98.89
0.3869	0.0305	1.44E-06	-	0.00067	23.23
0.0538	0.0677	1.54E-07	-	0.0008	27.56
0.2354	0.0362	9.98E-07	-	0.0007	23.99
0.7939	0.0371	1.44E-06	-	0.00068	23.28
0.0385	0.0963	1.64E-06	-	0.00067	22.96
0.0552	0.0765	1.95E-06	-	0.00066	22.64
0.4539	0.0304	6.21E-12	-	0.00137	47.40
0.0334	0.0952	2.64E-07	-	0.00077	26.49
0.5508	0.0296	3.63E-10	-	0.00114	39.40
0.3678	0.0314	1.09E-06	-	0.00069	23.83
0.7591	0.0347	2.07E-06	-	0.00065	22.53
0.1549	0.0414	7.44E-07	-	0.00071	24.45
0.3246	0.0315	7.23E-08	-	0.00084	28.99
0.6655	0.031	1.94E-07	-	0.00079	27.11
0.3504	0.0312	4.03E-06	-	0.00061	21.18
0.1915	0.0401	1.51E-08	-	0.00093	31.99
0.1858	0.0396	8.39E-10	-	0.00109	37.59
0.0132	0.1714	2.19E-06	-	0.00065	22.42

0.5494	0.0304	4.31E-07	-	0.00074	25.59
0.4888	0.0306	2.00E-07	-	0.00078	27.00
0.1217	0.0453	1.29E-08	-	0.00094	32.33
0.0779	0.0601	4.43E-06	-	0.00061	21.09
0.0718	0.0597	2.94E-06	-	0.00063	21.84
0.7966	0.0367	1.79E-07	-	0.00079	27.23
0.506	0.0303	1.43E-06	-	0.00067	23.22
0.3428	0.0338	8.52E-08	-	0.00083	28.71
0.0164	0.1641	3.79E-06	-	0.00062	21.36
0.1122	0.0491	2.60E-06	-	0.00064	22.13
0.1543	0.0424	7.92E-07	-	0.00071	24.41
0.0539	0.0684	2.51E-06	-	0.00064	22.16
0.0811	0.0562	1.59E-06	-	0.00067	22.99
0.1895	0.0385	7.49E-07	-	0.00071	24.43
0.04	0.0859	3.58E-07	-	0.00075	25.91
0.0265	0.1181	2.97E-06	-	0.00063	21.82
0.0976	0.0557	1.76E-06	-	0.00066	22.80
0.0375	0.0831	2.48E-06	rs1341068	0.00064	22.19
0.0246	0.12	8.88E-07	-	0.0007	24.17
0.0118	0.2214	1.75E-06	-	0.00066	22.84
0.169	0.0399	1.71E-06	-	0.00066	22.87
0.2933	0.0323	2.55E-08	-	0.0009	31.05
0.2408	0.0348	5.87E-07	-	0.00072	24.91
0.1885	0.037	6.97E-07	-	0.00071	24.57
0.3541	0.032	2.96E-07	-	0.00076	26.30
0.6028	0.0309	2.51E-06	-	0.00064	22.20
0.1021	0.0498	2.46E-06	-	0.00064	22.21
0.5333	0.0296	8.78E-09	-	0.00096	33.10
0.4237	0.0302	5.57E-07	-	0.00072	25.00
0.0537	0.0806	3.19E-06	-	0.00063	21.67
0.0708	0.0631	2.68E-06	-	0.00064	22.03
0.0741	0.0598	2.78E-06	-	0.00064	21.95
0.6514	0.0359	1.22E-06	-	0.00068	23.49
0.1088	0.0497	4.46E-06	-	0.00061	21.10
0.2169	0.0355	2.34E-06	-	0.00065	22.29
0.2017	0.0386	3.75E-06	-	0.00062	21.43
0.3815	0.031	5.09E-10	-	0.00112	38.68
0.4597	0.0294	1.64E-11	-	0.00132	45.49
0.1647	0.0418	7.52E-08	rs979041	0.00084	28.87
0.0381	58.1786	3.38E-24	-	0.00298	102.98
0.0294	78.3081	2.53E-06	-	0.00064	22.14
0.0322	63.7358	1.01E-07	-	0.00082	28.36

0.7352	0.0792	7.54E-07	-	0.00071	24.45
0.2731	0.0831	4.29E-06	-	0.00061	21.15
0.0111	125.806	3.12E-37	-	0.0047	162.55
0.0388	55.1122	1.95E-14	-	0.0017	58.58
0.0289	83.642	1.13E-07	-	0.00082	28.14
0.0417	0.2061	4.60E-06	-	0.00061	21.00
0.0182	85.1356	9.06E-14	-	0.00161	55.56
0.0358	67.4644	1.35E-07	-	0.00081	27.79
0.0212	82.8799	1.14E-07	-	0.00082	28.12
0.0259	76.0531	4.97E-06	-	0.0006	20.85
0.0286	71.6693	1.91E-07	-	0.00079	27.12
0.7018	0.033	8.92E-20	-	0.0024	82.97
0.7876	0.0362	2.61E-23	-	0.00286	98.89
0.3869	0.0305	1.44E-06	-	0.00067	23.23
0.0538	0.0677	1.54E-07	-	0.0008	27.56
0.2354	0.0362	9.98E-07	-	0.0007	23.99
0.7939	0.0371	1.44E-06	-	0.00068	23.28
0.0385	0.0963	1.64E-06	-	0.00067	22.96
0.0552	0.0765	1.95E-06	-	0.00066	22.64
0.4539	0.0304	6.21E-12	-	0.00137	47.40
0.0334	0.0952	2.64E-07	-	0.00077	26.49
0.5508	0.0296	3.63E-10	-	0.00114	39.40
0.3678	0.0314	1.09E-06	-	0.00069	23.83
0.7591	0.0347	2.07E-06	-	0.00065	22.53
0.1549	0.0414	7.44E-07	-	0.00071	24.45
0.3246	0.0315	7.23E-08	-	0.00084	28.99
0.6655	0.031	1.94E-07	-	0.00079	27.11
0.3504	0.0312	4.03E-06	-	0.00061	21.18
0.1915	0.0401	1.51E-08	-	0.00093	31.99
0.1858	0.0396	8.39E-10	-	0.00109	37.59
0.0132	0.1714	2.19E-06	-	0.00065	22.42
0.5494	0.0304	4.31E-07	-	0.00074	25.59
0.4888	0.0306	2.00E-07	-	0.00078	27.00
0.1217	0.0453	1.29E-08	-	0.00094	32.33
0.0779	0.0601	4.43E-06	-	0.00061	21.09
0.0718	0.0597	2.94E-06	-	0.00063	21.84
0.7966	0.0367	1.79E-07	-	0.00079	27.23
0.506	0.0303	1.43E-06	-	0.00067	23.22
0.3428	0.0338	8.52E-08	-	0.00083	28.71
0.1122	0.0491	2.60E-06	-	0.00064	22.13
0.1543	0.0424	7.92E-07	-	0.00071	24.41
0.0539	0.0684	2.51E-06	-	0.00064	22.16

0.0811	0.0562	1.59E-06	-	0.00067	22.99
0.1895	0.0385	7.49E-07	-	0.00071	24.43
0.04	0.0859	3.58E-07	-	0.00075	25.91
0.7415	0.048	1.05E-06	-	0.00069	23.89
0.3786	0.0397	3.83E-10	-	0.00113	39.15
0.0291	0.1557	1.37E-06	-	0.00068	23.31
0.184	0.0494	8.48E-09	-	0.00096	33.19
0.0546	0.0871	3.35E-21	-	0.00259	89.39
0.0425	0.0976	1.59E-08	-	0.00093	31.95
0.126	0.0588	5.57E-07	-	0.00073	25.07
0.2656	0.0436	1.34E-06	-	0.00068	23.40
0.4398	0.0384	3.22E-06	-	0.00063	21.73
0.3065	0.0423	2.11E-06	-	0.00065	22.51
0.4231	0.0384	2.05E-11	-	0.0013	44.89
0.4243	0.0381	1.54E-06	-	0.00067	23.14
0.4078	0.0378	3.28E-06	-	0.00063	21.68
0.4179	0.0388	9.22E-07	-	0.0007	24.08
0.1974	0.0482	3.51E-15	-	0.0018	62.02
0.2734	0.0422	4.92E-06	-	0.0006	20.85
0.4967	0.0375	3.51E-06	-	0.00063	21.55
0.8677	0.0764	3.04E-07	-	0.00076	26.26
0.7966	0.0472	1.90E-06	-	0.00066	22.74
0.7367	0.0424	2.07E-09	-	0.00104	35.94
0.3108	0.0412	2.27E-15	-	0.00182	62.80
0.2726	0.0435	3.07E-06	-	0.00063	21.80
0.0237	0.2048	2.80E-06	-	0.00064	21.94
0.0786	0.0768	1.74E-08	-	0.00092	31.77
0.0818	0.072	7.20E-15	-	0.00175	60.53
0.1455	0.0571	1.42E-06	-	0.00068	23.28
0.5625	0.0387	1.29E-06	-	0.00068	23.45
0.0205	0.1882	1.71E-06	-	0.00066	22.89
0.7573	0.0462	3.21E-06	-	0.00063	21.64
0.0857	0.0723	2.65E-07	-	0.00077	26.47
0.1267	0.0586	4.46E-06	-	0.00061	21.09
0.2457	0.0431	2.64E-07	-	0.00077	26.55
0.0265	0.1181	2.97E-06	-	0.00063	21.82
0.0976	0.0557	1.76E-06	-	0.00066	22.80
0.0375	0.0831	2.48E-06	-	0.00064	22.19
0.0246	0.12	8.88E-07	-	0.0007	24.17
0.169	0.0399	1.71E-06	-	0.00066	22.87
0.2933	0.0323	2.55E-08	-	0.0009	31.05
0.2408	0.0348	5.87E-07	-	0.00072	24.91

0.1885	0.037	6.97E-07	-	0.00071	24.57
0.3541	0.032	2.96E-07	-	0.00076	26.30
0.6028	0.0309	2.51E-06	-	0.00064	22.20
0.1021	0.0498	2.46E-06	-	0.00064	22.21
0.5333	0.0296	8.78E-09	-	0.00096	33.10
0.4237	0.0302	5.57E-07	-	0.00072	25.00
0.0537	0.0806	3.19E-06	-	0.00063	21.67
0.0708	0.0631	2.68E-06	-	0.00064	22.03
0.0741	0.0598	2.78E-06	-	0.00064	21.95
0.6514	0.0359	1.22E-06	-	0.00068	23.49
0.1088	0.0497	4.46E-06	-	0.00061	21.10
0.2169	0.0355	2.34E-06	-	0.00065	22.29
0.2017	0.0386	3.75E-06	-	0.00062	21.43
0.3815	0.031	5.09E-10	-	0.00112	38.68
0.4597	0.0294	1.64E-11	-	0.00132	45.49
0.1647	0.0418	7.52E-08	-	0.00084	28.87
0.0931	0.0014	1.93E-06	-	0.00061	20.90
0.0345	0.0022	4.61E-06	-	0.00061	21.08
0.6782	8.00E-04	1.17E-07	-	0.00072	25.00
0.7253	8.00E-04	5.08E-07	-	0.00072	25.00
0.0366	0.0021	1.29E-06	-	0.00068	23.59
0.6225	7.00E-04	2.43E-07	-	0.00081	27.94
0.1576	0.001	4.00E-06	-	0.00059	20.25
0.6313	7.00E-04	3.59E-08	-	0.00099	34.30
0.0583	0.0017	4.06E-06	-	0.00061	21.05
0.416	7.00E-04	1.70E-06	-	0.00072	25.00
0.1345	0.0011	2.69E-06	-	0.00058	19.84
0.4057	7.00E-04	1.03E-06	-	0.00077	26.45
0.1136	0.0012	1.56E-06	-	0.00063	21.78
0.5862	8.00E-04	2.13E-06	-	0.00059	20.25
0.0151	0.0036	3.02E-06	-	0.00065	22.30
0.0683	0.0015	1.13E-06	-	0.00065	22.40
0.3898	7.00E-04	2.46E-17	-	0.0022	75.93
0.0381	58.1786	3.38E-24	-	0.00298	102.98
0.0294	78.3081	2.53E-06	-	0.00064	22.14
0.0322	63.7358	1.01E-07	-	0.00082	28.36
0.7352	0.0792	7.54E-07	-	0.00071	24.45
0.2731	0.0831	4.29E-06	-	0.00061	21.15
0.0111	125.806	3.12E-37	-	0.0047	162.55
0.0388	55.1122	1.95E-14	-	0.0017	58.58
0.0289	83.642	1.13E-07	-	0.00082	28.14
0.0417	0.2061	4.60E-06	-	0.00061	21.00

0.0182	85.1356	9.06E-14	-	0.00161	55.56
0.0358	67.4644	1.35E-07	-	0.00081	27.79
0.0212	82.8799	1.14E-07	-	0.00082	28.12
0.0259	76.0531	4.97E-06	-	0.0006	20.85
0.0286	71.6693	1.91E-07	-	0.00079	27.12
0.7018	0.033	8.92E-20	-	0.0024	82.97
0.7876	0.0362	2.61E-23	-	0.00286	98.89
0.3869	0.0305	1.44E-06	-	0.00067	23.23
0.0538	0.0677	1.54E-07	-	0.0008	27.56
0.2354	0.0362	9.98E-07	-	0.0007	23.99
0.7939	0.0371	1.44E-06	-	0.00068	23.28
0.0385	0.0963	1.64E-06	-	0.00067	22.96
0.0552	0.0765	1.95E-06	-	0.00066	22.64
0.4539	0.0304	6.21E-12	-	0.00137	47.40
0.0334	0.0952	2.64E-07	-	0.00077	26.49
0.5508	0.0296	3.63E-10	-	0.00114	39.40
0.3678	0.0314	1.09E-06	-	0.00069	23.83
0.7591	0.0347	2.07E-06	-	0.00065	22.53
0.1549	0.0414	7.44E-07	-	0.00071	24.45
0.3246	0.0315	7.23E-08	-	0.00084	28.99
0.6655	0.031	1.94E-07	-	0.00079	27.11
0.3504	0.0312	4.03E-06	-	0.00061	21.18
0.1858	0.0396	8.39E-10	-	0.00109	37.59
0.0132	0.1714	2.19E-06	-	0.00065	22.42
0.5494	0.0304	4.31E-07	-	0.00074	25.59
0.4888	0.0306	2.00E-07	-	0.00078	27.00
0.1217	0.0453	1.29E-08	-	0.00094	32.33
0.0779	0.0601	4.43E-06	-	0.00061	21.09
0.0718	0.0597	2.94E-06	-	0.00063	21.84
0.7966	0.0367	1.79E-07	-	0.00079	27.23
0.506	0.0303	1.43E-06	-	0.00067	23.22
0.3428	0.0338	8.52E-08	-	0.00083	28.71
0.0164	0.1641	3.79E-06	-	0.00062	21.36
0.1122	0.0491	2.60E-06	-	0.00064	22.13
0.1543	0.0424	7.92E-07	-	0.00071	24.41
0.0539	0.0684	2.51E-06	-	0.00064	22.16
0.0811	0.0562	1.59E-06	-	0.00067	22.99
0.1895	0.0385	7.49E-07	-	0.00071	24.43
0.04	0.0859	3.58E-07	-	0.00075	25.91
0.0931	0.0014	1.93E-06	rs140581	0.00061	20.90
0.0345	0.0022	4.61E-06	-	0.00061	21.08
0.6782	8.00E-04	1.17E-07	-	0.00072	25.00

0.7253	8.00E-04	5.08E-07	-	0.00072	25.00
0.0366	0.0021	1.29E-06	-	0.00068	23.59
0.6225	7.00E-04	2.43E-07	-	0.00081	27.94
0.1576	0.001	4.00E-06	-	0.00059	20.25
0.6313	7.00E-04	3.59E-08	-	0.00099	34.30
0.0583	0.0017	4.06E-06	-	0.00061	21.05
0.416	7.00E-04	1.70E-06	-	0.00072	25.00
0.1345	0.0011	2.69E-06	-	0.00058	19.84
0.4057	7.00E-04	1.03E-06	-	0.00077	26.45
0.1136	0.0012	1.56E-06	-	0.00063	21.78
0.5862	8.00E-04	2.13E-06	-	0.00059	20.25
0.0151	0.0036	3.02E-06	-	0.00065	22.30
0.0683	0.0015	1.13E-06	-	0.00065	22.40
0.3898	7.00E-04	2.46E-17	-	0.0022	75.93
0.0265	0.1181	2.97E-06	-	0.00063	21.82
0.0976	0.0557	1.76E-06	-	0.00066	22.80
0.0375	0.0831	2.48E-06	-	0.00064	22.19
0.0246	0.12	8.88E-07	-	0.0007	24.17
0.0118	0.2214	1.75E-06	-	0.00066	22.84
0.169	0.0399	1.71E-06	-	0.00066	22.87
0.2933	0.0323	2.55E-08	-	0.0009	31.05
0.2408	0.0348	5.87E-07	-	0.00072	24.91
0.1885	0.037	6.97E-07	-	0.00071	24.57
0.3541	0.032	2.96E-07	-	0.00076	26.30
0.6028	0.0309	2.51E-06	-	0.00064	22.20
0.1021	0.0498	2.46E-06	-	0.00064	22.21
0.5333	0.0296	8.78E-09	-	0.00096	33.10
0.4237	0.0302	5.57E-07	-	0.00072	25.00
0.0537	0.0806	3.19E-06	-	0.00063	21.67
0.0708	0.0631	2.68E-06	-	0.00064	22.03
0.0741	0.0598	2.78E-06	-	0.00064	21.95
0.6514	0.0359	1.22E-06	-	0.00068	23.49
0.1088	0.0497	4.46E-06	-	0.00061	21.10
0.2169	0.0355	2.34E-06	-	0.00065	22.29
0.2017	0.0386	3.75E-06	-	0.00062	21.43
0.3815	0.031	5.09E-10	-	0.00112	38.68
0.4597	0.0294	1.64E-11	-	0.00132	45.49
0.1647	0.0418	7.52E-08	-	0.00084	28.87
0.0265	0.1181	2.97E-06	-	0.00063	21.82
0.0976	0.0557	1.76E-06	-	0.00066	22.80
0.0375	0.0831	2.48E-06	-	0.00064	22.19
0.0246	0.12	8.88E-07	-	0.0007	24.17

0.0118	0.2214	1.75E-06	-	0.00066	22.84
0.169	0.0399	1.71E-06	-	0.00066	22.87
0.2933	0.0323	2.55E-08	-	0.0009	31.05
0.2408	0.0348	5.87E-07	-	0.00072	24.91
0.1885	0.037	6.97E-07	-	0.00071	24.57
0.3541	0.032	2.96E-07	-	0.00076	26.30
0.6028	0.0309	2.51E-06	-	0.00064	22.20
0.1021	0.0498	2.46E-06	-	0.00064	22.21
0.5333	0.0296	8.78E-09	-	0.00096	33.10
0.4237	0.0302	5.57E-07	-	0.00072	25.00
0.0537	0.0806	3.19E-06	-	0.00063	21.67
0.0708	0.0631	2.68E-06	-	0.00064	22.03
0.0741	0.0598	2.78E-06	-	0.00064	21.95
0.6514	0.0359	1.22E-06	-	0.00068	23.49
0.1088	0.0497	4.46E-06	-	0.00061	21.10
0.2169	0.0355	2.34E-06	-	0.00065	22.29
0.2017	0.0386	3.75E-06	-	0.00062	21.43
0.4597	0.0294	1.64E-11	-	0.00132	45.49
0.1647	0.0418	7.52E-08	-	0.00084	28.87
0.0931	0.0014	1.93E-06	-	0.00061	20.90
0.0345	0.0022	4.61E-06	-	0.00061	21.08
0.6782	8.00E-04	1.17E-07	-	0.00072	25.00
0.7253	8.00E-04	5.08E-07	-	0.00072	25.00
0.0366	0.0021	1.29E-06	-	0.00068	23.59
0.6225	7.00E-04	2.43E-07	-	0.00081	27.94
0.1576	0.001	4.00E-06	-	0.00059	20.25
0.6313	7.00E-04	3.59E-08	-	0.00099	34.30
0.416	7.00E-04	1.70E-06	-	0.00072	25.00
0.1345	0.0011	2.69E-06	-	0.00058	19.84
0.4057	7.00E-04	1.03E-06	-	0.00077	26.45
0.1136	0.0012	1.56E-06	-	0.00063	21.78
0.0151	0.0036	3.02E-06	-	0.00065	22.30
0.0683	0.0015	1.13E-06	-	0.00065	22.40
0.3898	7.00E-04	2.46E-17	-	0.0022	75.93
0.7018	0.033	8.92E-20	-	0.0024	82.97
0.7876	0.0362	2.61E-23	-	0.00286	98.89
0.3869	0.0305	1.44E-06	-	0.00067	23.23
0.0538	0.0677	1.54E-07	-	0.0008	27.56
0.2354	0.0362	9.98E-07	-	0.0007	23.99
0.7939	0.0371	1.44E-06	-	0.00068	23.28
0.0385	0.0963	1.64E-06	-	0.00067	22.96
0.0552	0.0765	1.95E-06	-	0.00066	22.64

0.4539	0.0304	6.21E-12	-	0.00137	47.40
0.0334	0.0952	2.64E-07	-	0.00077	26.49
0.5508	0.0296	3.63E-10	-	0.00114	39.40
0.3678	0.0314	1.09E-06	-	0.00069	23.83
0.7591	0.0347	2.07E-06	-	0.00065	22.53
0.1549	0.0414	7.44E-07	-	0.00071	24.45
0.3246	0.0315	7.23E-08	-	0.00084	28.99
0.6655	0.031	1.94E-07	-	0.00079	27.11
0.3504	0.0312	4.03E-06	-	0.00061	21.18
0.1915	0.0401	1.51E-08	-	0.00093	31.99
0.0132	0.1714	2.19E-06	-	0.00065	22.42
0.5494	0.0304	4.31E-07	-	0.00074	25.59
0.4888	0.0306	2.00E-07	-	0.00078	27.00
0.1217	0.0453	1.29E-08	-	0.00094	32.33
0.0779	0.0601	4.43E-06	-	0.00061	21.09
0.0718	0.0597	2.94E-06	-	0.00063	21.84
0.7966	0.0367	1.79E-07	-	0.00079	27.23
0.506	0.0303	1.43E-06	-	0.00067	23.22
0.3428	0.0338	8.52E-08	-	0.00083	28.71
0.1122	0.0491	2.60E-06	-	0.00064	22.13
0.1543	0.0424	7.92E-07	-	0.00071	24.41
0.0539	0.0684	2.51E-06	-	0.00064	22.16
0.0811	0.0562	1.59E-06	-	0.00067	22.99
0.1895	0.0385	7.49E-07	-	0.00071	24.43
0.04	0.0859	3.58E-07	-	0.00075	25.91
0.0265	0.1181	2.97E-06	-	0.00063	21.82
0.0976	0.0557	1.76E-06	-	0.00066	22.80
0.0375	0.0831	2.48E-06	-	0.00064	22.19
0.0246	0.12	8.88E-07	-	0.0007	24.17
0.0118	0.2214	1.75E-06	-	0.00066	22.84
0.169	0.0399	1.71E-06	-	0.00066	22.87
0.2933	0.0323	2.55E-08	-	0.0009	31.05
0.2408	0.0348	5.87E-07	-	0.00072	24.91
0.1885	0.037	6.97E-07	-	0.00071	24.57
0.3541	0.032	2.96E-07	-	0.00076	26.30
0.6028	0.0309	2.51E-06	-	0.00064	22.20
0.1021	0.0498	2.46E-06	-	0.00064	22.21
0.5333	0.0296	8.78E-09	-	0.00096	33.10
0.4237	0.0302	5.57E-07	-	0.00072	25.00
0.0537	0.0806	3.19E-06	-	0.00063	21.67
0.0708	0.0631	2.68E-06	-	0.00064	22.03
0.0741	0.0598	2.78E-06	-	0.00064	21.95

0.6514	0.0359	1.22E-06	-	0.00068	23.49
0.1088	0.0497	4.46E-06	-	0.00061	21.10
0.2169	0.0355	2.34E-06	-	0.00065	22.29
0.2017	0.0386	3.75E-06	-	0.00062	21.43
0.4597	0.0294	1.64E-11	-	0.00132	45.49
0.1647	0.0418	7.52E-08	-	0.00084	28.87
0.7018	0.033	8.92E-20	-	0.0024	82.97
0.7876	0.0362	2.61E-23	-	0.00286	98.89
0.3869	0.0305	1.44E-06	-	0.00067	23.23
0.0538	0.0677	1.54E-07	-	0.0008	27.56
0.2354	0.0362	9.98E-07	-	0.0007	23.99
0.7939	0.0371	1.44E-06	-	0.00068	23.28
0.0385	0.0963	1.64E-06	-	0.00067	22.96
0.0552	0.0765	1.95E-06	-	0.00066	22.64
0.4539	0.0304	6.21E-12	-	0.00137	47.40
0.0334	0.0952	2.64E-07	-	0.00077	26.49
0.5508	0.0296	3.63E-10	-	0.00114	39.40
0.3678	0.0314	1.09E-06	-	0.00069	23.83
0.7591	0.0347	2.07E-06	-	0.00065	22.53
0.1549	0.0414	7.44E-07	-	0.00071	24.45
0.3246	0.0315	7.23E-08	-	0.00084	28.99
0.6655	0.031	1.94E-07	-	0.00079	27.11
0.3504	0.0312	4.03E-06	-	0.00061	21.18
0.1915	0.0401	1.51E-08	-	0.00093	31.99
0.0132	0.1714	2.19E-06	-	0.00065	22.42
0.5494	0.0304	4.31E-07	-	0.00074	25.59
0.4888	0.0306	2.00E-07	-	0.00078	27.00
0.1217	0.0453	1.29E-08	-	0.00094	32.33
0.0779	0.0601	4.43E-06	-	0.00061	21.09
0.0718	0.0597	2.94E-06	-	0.00063	21.84
0.7966	0.0367	1.79E-07	-	0.00079	27.23
0.506	0.0303	1.43E-06	-	0.00067	23.22
0.3428	0.0338	8.52E-08	-	0.00083	28.71
0.1122	0.0491	2.60E-06	-	0.00064	22.13
0.1543	0.0424	7.92E-07	-	0.00071	24.41
0.0539	0.0684	2.51E-06	-	0.00064	22.16
0.0811	0.0562	1.59E-06	-	0.00067	22.99
0.1895	0.0385	7.49E-07	-	0.00071	24.43
0.04	0.0859	3.58E-07	-	0.00075	25.91
0.0931	0.0014	1.93E-06	-	0.00061	20.90
0.0345	0.0022	4.61E-06	-	0.00061	21.08
0.6782	8.00E-04	1.17E-07	-	0.00072	25.00

0.7253	8.00E-04	5.08E-07	-	0.00072	25.00
0.0366	0.0021	1.29E-06	-	0.00068	23.59
0.6225	7.00E-04	2.43E-07	-	0.00081	27.94
0.1576	0.001	4.00E-06	-	0.00059	20.25
0.6313	7.00E-04	3.59E-08	-	0.00099	34.30
0.416	7.00E-04	1.70E-06	-	0.00072	25.00
0.1345	0.0011	2.69E-06	-	0.00058	19.84
0.4057	7.00E-04	1.03E-06	-	0.00077	26.45
0.1136	0.0012	1.56E-06	-	0.00063	21.78
0.0151	0.0036	3.02E-06	-	0.00065	22.30
0.0683	0.0015	1.13E-06	-	0.00065	22.40
0.3898	7.00E-04	2.46E-17	-	0.0022	75.93
0.7415	0.048	1.05E-06	-	0.00069	23.89
0.3786	0.0397	3.83E-10	-	0.00113	39.15
0.0092	0.3094	1.64E-06	-	0.00067	22.97
0.0291	0.1557	1.37E-06	-	0.00068	23.31
0.184	0.0494	8.48E-09	-	0.00096	33.19
0.0546	0.0871	3.35E-21	-	0.00259	89.39
0.0425	0.0976	1.59E-08	-	0.00093	31.95
0.126	0.0588	5.57E-07	-	0.00073	25.07
0.2656	0.0436	1.34E-06	-	0.00068	23.40
0.4398	0.0384	3.22E-06	-	0.00063	21.73
0.3065	0.0423	2.11E-06	-	0.00065	22.51
0.4231	0.0384	2.05E-11	-	0.0013	44.89
0.4243	0.0381	1.54E-06	-	0.00067	23.14
0.4078	0.0378	3.28E-06	-	0.00063	21.68
0.4179	0.0388	9.22E-07	-	0.0007	24.08
0.1974	0.0482	3.51E-15	-	0.0018	62.02
0.2734	0.0422	4.92E-06	-	0.0006	20.85
0.4967	0.0375	3.51E-06	-	0.00063	21.55
0.7966	0.0472	1.90E-06	-	0.00066	22.74
0.7367	0.0424	2.07E-09	-	0.00104	35.94
0.3108	0.0412	2.27E-15	-	0.00182	62.80
0.2726	0.0435	3.07E-06	-	0.00063	21.80
0.0237	0.2048	2.80E-06	-	0.00064	21.94
0.0786	0.0768	1.74E-08	-	0.00092	31.77
0.0818	0.072	7.20E-15	-	0.00175	60.53
0.1455	0.0571	1.42E-06	-	0.00068	23.28
0.5625	0.0387	1.29E-06	-	0.00068	23.45
0.0205	0.1882	1.71E-06	-	0.00066	22.89
0.7573	0.0462	3.21E-06	-	0.00063	21.64
0.0857	0.0723	2.65E-07	-	0.00077	26.47

0.1267	0.0586	4.46E-06	-	0.00061	21.09
0.2457	0.0431	2.64E-07	-	0.00077	26.55
0.0381	58.1786	3.38E-24	-	0.00298	102.98
0.0294	78.3081	2.53E-06	-	0.00064	22.14
0.0322	63.7358	1.01E-07	-	0.00082	28.36
0.7352	0.0792	7.54E-07	-	0.00071	24.45
0.2731	0.0831	4.29E-06	-	0.00061	21.15
0.0111	125.806	3.12E-37	-	0.0047	162.55
0.0388	55.1122	1.95E-14	-	0.0017	58.58
0.0289	83.642	1.13E-07	-	0.00082	28.14
0.0417	0.2061	4.60E-06	-	0.00061	21.00
0.0182	85.1356	9.06E-14	-	0.00161	55.56
0.0358	67.4644	1.35E-07	-	0.00081	27.79
0.0212	82.8799	1.14E-07	-	0.00082	28.12
0.0259	76.0531	4.97E-06	-	0.0006	20.85
0.0286	71.6693	1.91E-07	-	0.00079	27.12
0.7415	0.048	1.05E-06	-	0.00069	23.89
0.3786	0.0397	3.83E-10	-	0.00113	39.15
0.0092	0.3094	1.64E-06	-	0.00067	22.97
0.0291	0.1557	1.37E-06	-	0.00068	23.31
0.184	0.0494	8.48E-09	-	0.00096	33.19
0.0546	0.0871	3.35E-21	-	0.00259	89.39
0.0425	0.0976	1.59E-08	-	0.00093	31.95
0.126	0.0588	5.57E-07	-	0.00073	25.07
0.2656	0.0436	1.34E-06	-	0.00068	23.40
0.4398	0.0384	3.22E-06	-	0.00063	21.73
0.3065	0.0423	2.11E-06	-	0.00065	22.51
0.4231	0.0384	2.05E-11	-	0.0013	44.89
0.4243	0.0381	1.54E-06	-	0.00067	23.14
0.4078	0.0378	3.28E-06	-	0.00063	21.68
0.4179	0.0388	9.22E-07	-	0.0007	24.08
0.1974	0.0482	3.51E-15	-	0.0018	62.02
0.2734	0.0422	4.92E-06	-	0.0006	20.85
0.4967	0.0375	3.51E-06	-	0.00063	21.55
0.8677	0.0764	3.04E-07	-	0.00076	26.26
0.7966	0.0472	1.90E-06	-	0.00066	22.74
0.7367	0.0424	2.07E-09	-	0.00104	35.94
0.3108	0.0412	2.27E-15	-	0.00182	62.80
0.2726	0.0435	3.07E-06	-	0.00063	21.80
0.0237	0.2048	2.80E-06	-	0.00064	21.94
0.0786	0.0768	1.74E-08	-	0.00092	31.77
0.0818	0.072	7.20E-15	-	0.00175	60.53

0.1455	0.0571	1.42E-06	-	0.00068	23.28
0.5625	0.0387	1.29E-06	-	0.00068	23.45
0.0205	0.1882	1.71E-06	-	0.00066	22.89
0.7573	0.0462	3.21E-06	-	0.00063	21.64
0.0857	0.0723	2.65E-07	-	0.00077	26.47
0.1267	0.0586	4.46E-06	-	0.00061	21.09
0.2457	0.0431	2.64E-07	-	0.00077	26.55
0.0381	58.1786	3.38E-24	-	0.00298	102.98
0.0294	78.3081	2.53E-06	-	0.00064	22.14
0.0322	63.7358	1.01E-07	-	0.00082	28.36
0.7352	0.0792	7.54E-07	-	0.00071	24.45
0.2731	0.0831	4.29E-06	-	0.00061	21.15
0.0111	125.806	3.12E-37	-	0.0047	162.55
0.0388	55.1122	1.95E-14	-	0.0017	58.58
0.0289	83.642	1.13E-07	-	0.00082	28.14
0.0417	0.2061	4.60E-06	-	0.00061	21.00
0.0182	85.1356	9.06E-14	-	0.00161	55.56
0.0358	67.4644	1.35E-07	-	0.00081	27.79
0.0212	82.8799	1.14E-07	-	0.00082	28.12
0.0259	76.0531	4.97E-06	-	0.0006	20.85
0.0286	71.6693	1.91E-07	-	0.00079	27.12
0.0381	58.1786	3.38E-24	-	0.00298	102.98
0.0294	78.3081	2.53E-06	-	0.00064	22.14
0.0322	63.7358	1.01E-07	-	0.00082	28.36
0.7352	0.0792	7.54E-07	-	0.00071	24.45
0.2731	0.0831	4.29E-06	-	0.00061	21.15
0.0111	125.806	3.12E-37	-	0.0047	162.55
0.0388	55.1122	1.95E-14	-	0.0017	58.58
0.0417	0.2061	4.60E-06	-	0.00061	21.00
0.0182	85.1356	9.06E-14	-	0.00161	55.56
0.0358	67.4644	1.35E-07	-	0.00081	27.79
0.0212	82.8799	1.14E-07	-	0.00082	28.12
0.0259	76.0531	4.97E-06	-	0.0006	20.85
0.0286	71.6693	1.91E-07	-	0.00079	27.12
0.7415	0.048	1.05E-06	-	0.00069	23.89
0.3786	0.0397	3.83E-10	-	0.00113	39.15
0.0092	0.3094	1.64E-06	-	0.00067	22.97
0.0291	0.1557	1.37E-06	-	0.00068	23.31
0.184	0.0494	8.48E-09	-	0.00096	33.19
0.0546	0.0871	3.35E-21	-	0.00259	89.39
0.0425	0.0976	1.59E-08	-	0.00093	31.95
0.126	0.0588	5.57E-07	-	0.00073	25.07

0.2656	0.0436	1.34E-06	-	0.00068	23.40
0.4398	0.0384	3.22E-06	-	0.00063	21.73
0.3065	0.0423	2.11E-06	-	0.00065	22.51
0.4231	0.0384	2.05E-11	-	0.0013	44.89
0.4243	0.0381	1.54E-06	-	0.00067	23.14
0.4078	0.0378	3.28E-06	-	0.00063	21.68
0.4179	0.0388	9.22E-07	-	0.0007	24.08
0.1974	0.0482	3.51E-15	-	0.0018	62.02
0.2734	0.0422	4.92E-06	-	0.0006	20.85
0.4967	0.0375	3.51E-06	-	0.00063	21.55
0.8677	0.0764	3.04E-07	-	0.00076	26.26
0.7966	0.0472	1.90E-06	-	0.00066	22.74
0.7367	0.0424	2.07E-09	-	0.00104	35.94
0.3108	0.0412	2.27E-15	-	0.00182	62.80
0.2726	0.0435	3.07E-06	-	0.00063	21.80
0.0786	0.0768	1.74E-08	-	0.00092	31.77
0.0818	0.072	7.20E-15	-	0.00175	60.53
0.1455	0.0571	1.42E-06	-	0.00068	23.28
0.5625	0.0387	1.29E-06	-	0.00068	23.45
0.0205	0.1882	1.71E-06	-	0.00066	22.89
0.7573	0.0462	3.21E-06	-	0.00063	21.64
0.0857	0.0723	2.65E-07	-	0.00077	26.47
0.1267	0.0586	4.46E-06	-	0.00061	21.09
0.2457	0.0431	2.64E-07	-	0.00077	26.55
